# Synthesis and Activity against *Mycobacterium tuberculosis* of Olivacine and Oxygenated Derivatives

**DOI:** 10.3390/molecules23061402

**Published:** 2018-06-09

**Authors:** Ulrike Schmidt, Gabriele Theumer, Anne Jäger, Olga Kataeva, Baojie Wan, Scott G. Franzblau, Hans-Joachim Knölker

**Affiliations:** 1 Faculty of Chemistry, Technische Universität Dresden, Bergstraße 66, 01069 Dresden, Germany; ulrike.schmidt@chemie.tu-dresden.de (U.S.); Gabriele.Theumer@tu-dresden.de (G.T.); anne.jaeger@chemie.tu-dresden.de (A.J.); 2 A. M. Butlerov Chemistry Institute, Kazan Federal University, Kremlevskaya Str. 18, Kazan 420008, Russia; olga-kataeva@yandex.ru; 3 Institute for Tuberculosis Research, College of Pharmacy, University of Illinois at Chicago, 833 S. Wood St., MC 964, Chicago, IL 60612-7231, USA; baojie@uic.edu (B.W.); sgf@uic.edu (S.G.F.)

**Keywords:** inhibitory activity, catalysis, cyclization, olivacine, palladium, pyrido[4,3-*b*]carbazoles

## Abstract

The tetracyclic pyrido[4,3-*b*]carbazole olivacine and four of its oxygenated derivatives have been synthesized by a late-stage palladium-catalyzed Heck-type cyclization of the pyrrole ring as a key step. In a test for the inhibition of the growth of *Mycobacterium tuberculosis*, 9-methoxyolivacine showed the most significant inhibitory activity against *Mycobacterium tuberculosis,* with an MIC_90_ value of 1.5 μM.

## 1. Introduction

**Figure 1:**
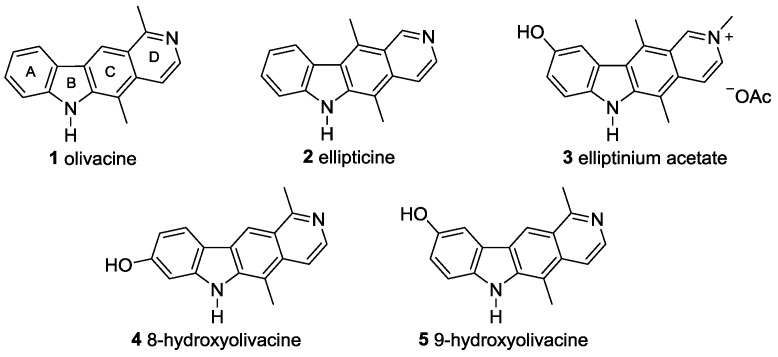
Pyrido[4,3-*b*]carbazole alkaloids and oxygenated derivatives.

The pyrido[4,3-*b*]carbazole alkaloid olivacine (**1**, Figure 1) was first isolated in 1958 by Schmutz et al. [1], and its structural assignment was confirmed by total synthesis only two years later [2]. The tetracyclic alkaloid **1** and many structurally related compounds, for example the isomeric natural product ellipticine (**2**), show useful biological activities, such as anti-tumor activity, based on DNA intercalation, topoisomerase II inhibition, and antimalarial activity [3,4,5,6,7]. Since the 1980s, A-ring oxygenated derivatives of ellipticine (**2**) have attracted much attention because of their anti-tumor activity [8]. Elliptinium acetate (**3**) has reached the status of a licensed drug for the treatment of advanced breast cancer [9]. Diverse total syntheses of olivacine (**1**) have been reported [10,11,12,13,14,15,16,17,18]. Surprisingly, the pharmacological potential of olivacine (**1**) and its oxygenated derivatives (for example **4** and **5**) has been much less investigated [19].

Although 9-hydroxyolivacine (**5**) is the main derivative produced by the metabolic conversion of olivacine (**1**) [3], derivatives of olivacine (**1**) with A-ring substitution have been not described extensively in the literature [3,11,13,20]. This may be because the syntheses of pyrido[4,3-*b*]carbazoles usually involve the annulation of an isoquinoline or a pyridine at an indole or carbazole framework [8,10,11]. Thus, a facile variation of the substitution pattern at ring A is not easy to accomplish. Herein, we present a novel route for the synthesis of the tetracyclic pyrido[4,3-*b*]carbazole framework [21].

## 2. Results and Discussion

**Scheme 1:**
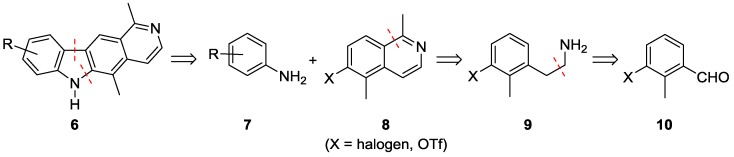
Retrosynthetic analysis for the pyrido[4,3-*b*]carbazole olivacine and its A-ring derivatives.

**Scheme 2:**
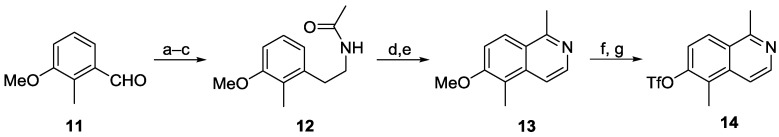
Synthesis of the triflate **14**. *Reagents and conditions*: (**a**) MeNO_2_, NH_4_OAc, AcOH, 80 °C, 110 min, 77%; (**b**) LiAlH_4_, THF, 0 °C to reflux, 19.5 h, 92%; (**c**) Ac_2_O, cat. DMAP, pyridine, 0 °C, 4 h, 99%; (**d**) POCl_3_, reflux, 1 h, 99%; (**e**) Pd/C (10%), cyclohexene, PhMe, reflux, 1.5 h, 100%; (**f**) pyridinium chloride, microwave (300 W), 155 °C, 30 min, 96%; (**g**) Tf_2_O, pyridine, MeCN, 0 °C, 20 h, 87%.

For a convergent access to various A-ring substituted derivatives, we envisaged a late-stage B-ring construction of the pyrido[4,3-*b*]carbazole framework. Therefore, we applied the two-step sequence of palladium-catalyzed reactions developed by our group for carbazole assembly: synthesis of a diarylamine via Buchwald–Hartwig coupling of appropriate anilines **7** with a substituted isoquinoline **8** followed by oxidative cyclization to the pyrido[4,3-*b*]carbazoles **6** (Scheme 1) [11]. The isoquinoline **8** would be available by Bischler–Napieralski cyclization of the arylethylamine **9** via the corresponding acetamide. Henry reaction of an appropriately substituted benzaldehyde **10** and subsequent reduction should afford the arylethylamine **9**. As the Bischler–Napieralski reaction works best on electron rich aromatic systems, we decided to start from the commercially available methoxy-substituted benzaldehyde **11** (Scheme 2) and to transform the methoxy group into a suitable leaving group at a later stage of our synthesis.

### 2.1. Total Synthesis

Starting from the commercial benzaldehyde **11**, which can also be obtained in one step and 87% yield from the much cheaper *m*-anisaldehyde [22], amide **12** is prepared by a three-step sequence of Henry reaction, lithium aluminum hydride reduction, and N-acetylation (Scheme 2) [23]. Bischler–Napieralski cyclization using phosphorus oxychloride led to the corresponding dihydroisoquinoline, which was fully aromatized to 6-methoxy-1,5-dimethylisoquinoline (**13**) by dehydrogenation with palladium on charcoal in the presence of cyclohexene as additive. Cleavage of the methyl ether afforded the isoquinolinol, which on reaction with trifluoromethanesulfonic anhydride provided the known isoquinolinyl triflate **14** [24] in 58% yield over seven steps.

**Scheme 3:**
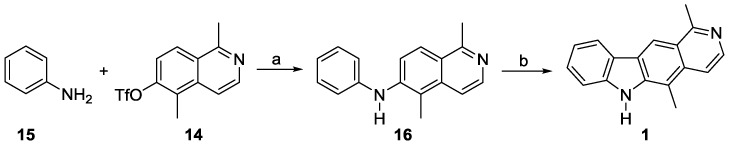
Synthesis of olivacine (**1**) via oxidative cyclization. *Reagents and conditions*: (**a**) cat. Pd(OAc)_2_, cat. XPhos, Cs_2_CO_3_, PhMe, reflux, 48 h, 100%; (**b**) 1.1 equiv. Pd(OAc)_2_, AcOH, 80–100 °C, 24 h, argon, 9–49%.

Buchwald–Hartwig coupling [25] of the triflate **14** and aniline (**15**) provided the diarylamine **16** (Scheme 3). However, the oxidative cyclization to afford the pyrido[4,3-*b*]carbazole framework proved to be very difficult [26]. Several attempts to optimize this reaction failed, which included using different reaction temperatures, different solvents (HOAc, HOPiv, dioxane, toluene), catalytic amounts of palladium(II) acetate in the presence of different re-oxidants, as well as stoichiometric amounts of palladium(II) acetate [27,28,29]. All of these experiments resulted to a large extent in decomposition, and led to olivacine (**1**) in only low to moderate yields with poor reproducibility.

**Scheme 4:**
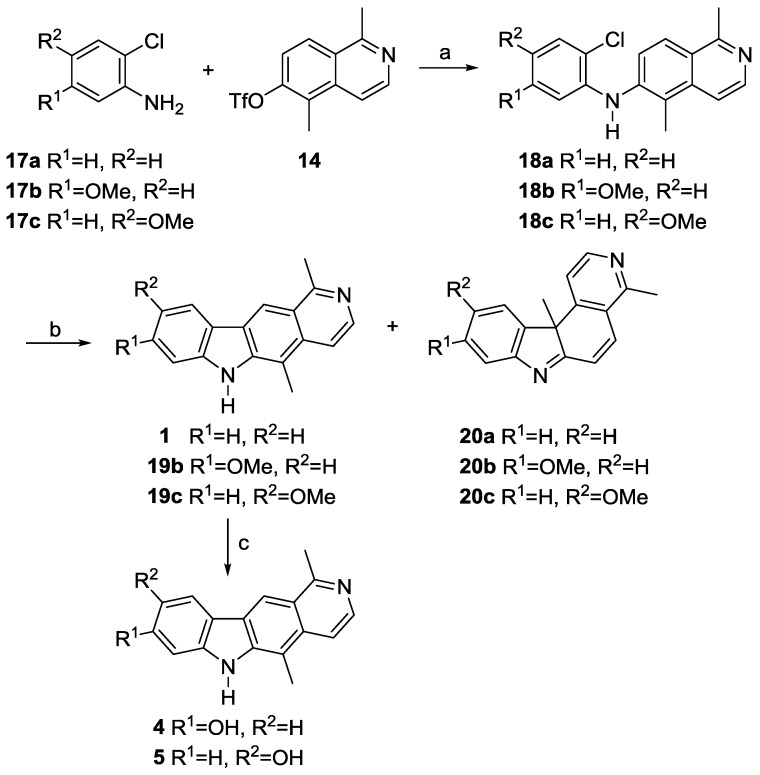
Synthesis of the pyrido[4,3-*b*]carbazoles **1**, **4** and **5**. *Reagents and conditions*: (**a**) cat. Pd(OAc)_2_, cat. XPhos, Cs_2_CO_3_, PhMe, reflux, 1–5 h, 83–94% (**18a**–**c**); (**b**) cat. Pd(OAc)_2_, P(*t*Bu)_3_·HBF_4_, K_2_CO_3_, DMF, 140 °C, 20–35 min, 62–71% (**1**, **19b**, **19c**), 3–12% (**20a**–**c**); (**c**) HBr_(aq)_, reflux, 24 h, 70–84% (**4**, **5**).

**Figure 2:**
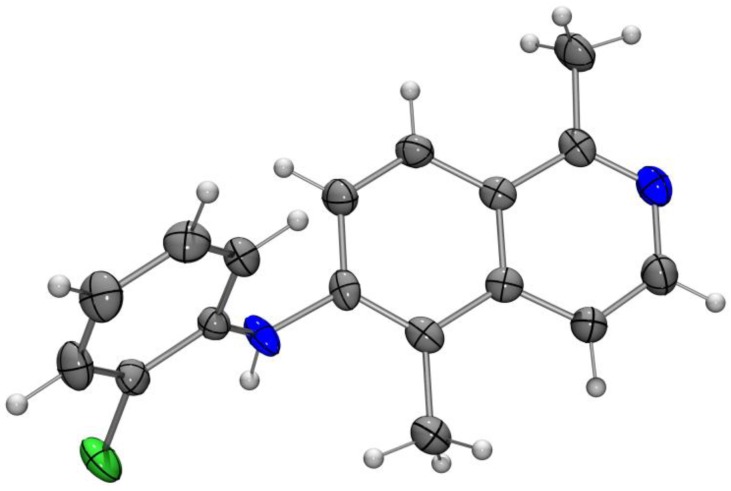
Molecular structure of the diarylamine **18a** in the crystal (ORTEP plot showing thermal ellipsoids at the 50% probability level).

Therefore, we decided to apply a Heck-type cyclization for the formation of the crucial carbon–carbon bond of the central pyrrole ring. This approach was already described by Sakamoto et al. in 1999 [30]. Buchwald–Hartwig coupling of the triflate **14** with the commercially available *o*-chloroanilines **17a**–**c** led to the corresponding diarylamines **18a**–**c** in 83–94% yield (Scheme 4). Compound **18a** was structurally confirmed by an X-ray analysis (Figure 2).

**Figure 3:**
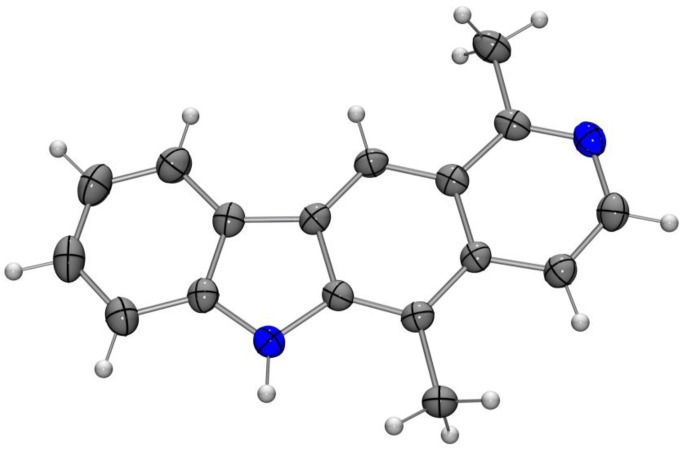
Molecular structure of olivacine (**1**) in the crystal (ORTEP plot showing thermal ellipsoids at the 50% probability level).

The cyclization reaction of the diarylamine **18a** with catalytic amounts of palladium(II) acetate in the presence of P(*t*Bu)_3_·HBF_4_ and K_2_CO_3_ in DMA at 110 °C or in DMF at 120 °C [31,32] proceeded very slowly and gave the product in only moderate yields after 1–2 days (Table 1, entries 1 and 4). Hydrodehalogenation leading to compound **16** was the major side reaction. Using only slightly higher temperatures (130–140 °C), the reaction proceeded much faster, and the yields for olivacine (**1**) were significantly better (Table 1, entries 2, 5, and 6). Finally, using larger amounts of the catalyst combined with shorter reaction times, olivacine (**1**) was obtained in 71% yield. The structure of **1** was confirmed by an X-ray crystal structure determination (Figure 3).

**Figure 4:**
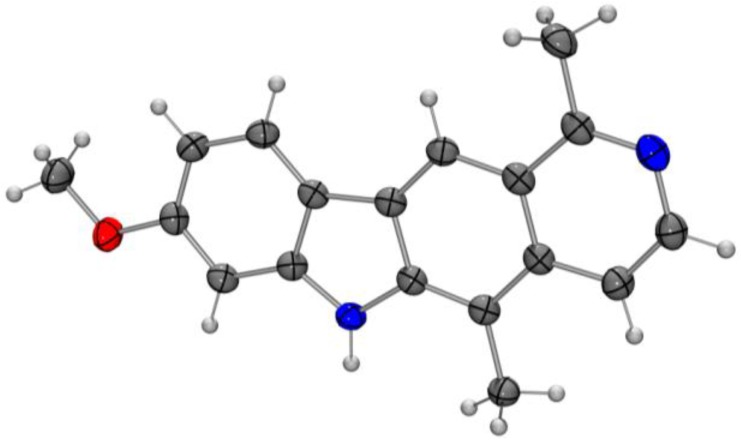
Molecular structure of 8-methoxyolivacine (**19b**) in the crystal (ORTEP plot showing thermal ellipsoids at the 50% probability level).

Application of these conditions to the cyclization of the diarylamines **18b** and **18c** provided 8-methoxyolivacine (**19b**) and 9-methoxyolivacine (**19c**) in 65% and 62% yield, respectively (Scheme 4). The structure of 8-methoxyolivacine (**19b**) was additionally confirmed by an X-ray analysis of single crystals (Figure 4). 9-Methoxyolivacine (**19c**) is a natural product that was isolated in 1967 from the bark of the coastal Venezuelan tree *Aspidosperma vargasii* A. DC. [33], and has been synthesized previously [3,13,20]. Interestingly, the 11b*H*-pyrido[3,4-*c*]carbazoles **20a**–**c** containing a quaternary carbon atom were obtained as byproducts of the cyclization reactions of the diarylamines **18a**–**c** in up to 12% yield. The structural assignments for the 11b*H*-pyrido[3,4-*c*]carbazoles **20a**–**c** were supported by two-dimensional (2D) NMR (COSY, HMBC, HSQC, NOESY) spectroscopic studies (see Supplementary Materials). The compounds **20a**–**c** result from an attack at the C5 carbon atom of the isoquinoline moiety. Cleavage of the methyl ether of **19b** and **19c** provided 8-hydroxyolivacine (**4**) and 9-hydroxyolivacine (**5**) [3] in 84% and 70% yield, respectively. For biological testing, the products were additionally purified by HPLC.

### 2.2. Biological Activity

A weak inhibitory activity against *Mycobacterium tuberculosis* was described in early reports for some simple tricyclic carbazole alkaloids [34,35,36]. Based on that work, we investigated the inhibitory activity of a range of oxygenated carbazole alkaloids and their derivatives, and found very promising results for several compounds [37,38,39]. Therefore, we also tested olivacine (**1**) and its oxygenated derivatives **4**, **5**, **19b**, and **19c** for their inhibition of *M. tuberculosis* (Table 2).

In a preliminary activity test against *M. tuberculosis*, only two of the five pyrido[4,3-*b*]carbazoles, namely olivacine (**1**) and 9-methoxyolivacine (**19c**), showed significant effects and have been studied further. The minimum concentrations effecting a 90% inhibition of growth (MIC_90_) of the *M. tuberculosis* strain H_37_Rv were determined by the microplate Alamar blue assay (MABA) [40,41]. The in vitro cytotoxicity towards mammalian (vero) cells was determined as described previously [40,42].

The MIC_90_ value for 3-methoxy-2-methylcarbazole-1,4-quinone served as a benchmark for comparison with the inhibitory activities of carbazoles that were found in our previous studies [39]. Although olivacine (**1**) shows an activity comparable to our benchmark compound, the SI value is considerably lower (SI = 3.8) due to its toxicity. However, 9-methoxyolivacine (**19c**) exhibits a strong inhibition of *M. tuberculosis* (MIC_90_ = 1.5 μM) combined with a lower cytotoxicity towards mammalian cells, which leads to a very good selectivity index (SI = 16.3).

## 3. Materials and Methods

### 3.1. General

All of the reactions were carried out in oven-dried glassware using anhydrous solvents under an argon atmosphere, unless stated otherwise. CH_2_Cl_2_, THF, and toluene were dried using a solvent purification system (MB-SBS, M. Braun Inertgas-Systeme GmbH, Garching, Germany). The petroleum ether that was used refers to the hydrocarbon mixture with a boiling range of 40–65 °C. Pd(OAc)_2_ was recrystallized from glacial AcOH. All of the other chemicals were used as received from commercial sources. A microwave reactor (CEM Discover, CEM GmbH, Kamp-Lintfort, Germany) was utilized for reactions taking place under microwave irradiation. Flash chromatography was performed using silica gel (0.035–0.070 mm) from Acros Organics (Thermo Fisher Scientific, Waltham, MA, USA). Alox N (aluminum oxide 90 active neutral) was obtained from Merck (Merck KGaA, Darmstadt, Germany). TLC was performed with TLC plates (silica gel 60 F_254_) from Merck (Merck KGaA, Darmstadt, Germany) using UV light for visualization. Melting points were measured on a Gallenkamp MPD 350 melting point apparatus (A. Gallenkamp & Co. Ltd, London, UK). Ultraviolet spectra were recorded on a PerkinElmer 25 UV/Vis spectrometer (Perkin Elmer, Waltham, MA, USA). Fluorescence spectra were obtained using a Varian Cary Eclipse spectrometer (Agilent, Santa Clara, CA, USA). IR spectra were recorded on a Thermo Nicolet Avatar 360 FT-IR spectrometer (Thermo Fisher Scientific, Waltham, MA, USA) using the ATR method (Attenuated Total Reflectance). NMR spectra were recorded on Bruker DRX 500 (Bruker Corp., Billerica, MA, USA) and Avance III 600 (Bruker Corp., Billerica, MA, USA) spectrometers. Chemical shifts *δ* are reported in parts per million (ppm) with the solvent signal as an internal standard. Standard abbreviations were used to denote the multiplicities of the signals. MS and HRMS (EI) were recorded on a Finnigan MAT-95 spectrometer (electron impact, 70 eV, Thermo Fisher Scientific, Waltham, MA, USA) or by GC/MS-coupling using an Agilent Technologies 6890 N GC System equipped with a 5973 Mass Selective Detector (electron impact, 70 eV, Agilent Technologies, Santa Clara, CA, USA). ESI-MS spectra were recorded on an Esquire LC with an ion trap detector from Bruker (Bruker Corp., Billerica, MA, USA). Positive and negative ions were detected. ESI-HRMS were recorded using a Q-TOF 6538 (Agilent Technologies, Santa Clara, CA, USA). Elemental analyses were measured on an EuroVector EuroEA3000 elemental analyzer (Eurovector Srl, Pavia, Italy). X-ray crystal structure analyses were performed with a Bruker-Nonius Kappa CCD (Bruker Corp., Billerica, MA, USA) that was equipped with a 700 series Cryostream low-temperature device from Oxford Cryosystems (Oxford Cryosystems Ltd, Long Hanborough, UK). SHELXS-97 [43], SADABS version 2.10 [44], SHELXL-97 [45], POV-Ray for Windows version 3.7.0.msvc10.win64 (Persistence of Vision Raytracer Pty. Ltd, Williamstown, Australia), and ORTEP-3 for Windows [46] (University of Glasgow, Glasgow, UK) were used as software.

### 3.2. Procedures

*1-Methoxy-2-methyl-3-(2-nitrovinyl)benzene.* Nitromethane (427 mg, 6.99 mmol) and freshly sublimated ammonium acetate (433 mg, 5.62 mmol) were added to a solution of 3-methoxy-2-methylbenzaldehyde (**11**, 800 mg, 5.33 mmol) in acetic acid (645 mg, 10.74 mmol), and the mixture was stirred at 80 °C for 1 h 50 min. After cooling to room temperature, the precipitate was dissolved by adding ethyl acetate. The mixture was transferred to a separatory funnel, and then washed twice with water and brine. The aqueous layer was extracted with ethyl acetate, the combined organic layers were dried (magnesium sulfate), and the solvent was evaporated. Purification of the residue by column chromatography (silica gel, petroleum ether/ethyl acetate, 1% to 15% ethyl acetate) provided 1-methoxy-2-methyl-3-(2-nitrovinyl)benzene (791 mg, 4.09 mmol, 77%) as yellow crystals. M.p. 97–98 °C; UV (MeOH): *λ* = 205, 228, 251, 317 nm; IR (ATR): *ν* = 3116, 2959, 2920, 2838, 1901, 1820, 1697, 1653, 1627, 1594, 1573, 1541, 1498, 1477, 1450, 1331, 1260, 1244, 1102, 1080, 1007, 968, 893, 873, 844, 806, 777, 725, 693 cm^–1^; ^1^H-NMR (500 MHz, CDCl_3_): *δ* = 2.33 (s, 3H), 3.86 (s, 3H), 6.96 (d, *J* = 8.2 Hz, 1H), 7.10 (d, *J* = 7.8 Hz, 1H), 7.22 (t, *J* = 8.0 Hz, 1H), 7.48 (d, *J* = 13.5 Hz, 1H), 8.33 (d, *J* = 13.5 Hz, 1H); ^13^C-NMR (125 MHz, CDCl_3_): *δ* = 11.96 (CH_3_), 55.84 (CH_3_), 113.12 (CH), 119.25 (CH), 127.11 (CH), 128.40 (C), 130.13 (C), 137.25 (CH), 138.20 (CH), 158.29 (C); MS (EI): *m*/*z* (%) = 193 (100, [M]^+^), 178 (6), 161 (7), 146 (70), 131 (52), 115 (54), 103 (67), 91 (37), 77 (47), 63 (18), 51 (18); HRMS: calcd. for C_10_H_11_NO_3_: 193.0738, found: 193.0733; elemental analysis: calcd. for C_10_H_11_NO_3_: C: 62.17, H: 5.74, N: 7.25; found: C: 62.16, H: 5.77, N: 7.50.

*2-(3-Methoxy-2-methylphenyl)ethanamine.* Over a period of 1 h, a solution of 1-methoxy-2-methyl-3(2-nitrovinyl)benzene (6.79 g, 35.2 mmol) in THF (95 mL) was added to a suspension of lithium aluminum hydride (6.83 g, 180 mmol) in THF (360 mL) at 0 °C. The cooling bath was removed, and the mixture was heated for 30 min at room temperature and 18 h at reflux. A second portion of lithium aluminum hydride (0.35 g, 9.1 mmol) was added to the slightly reddish-colored solution, and the mixture was heated at reflux for an additional hour. After cooling to room temperature, the reaction mixture was carefully quenched with saturated aqueous ammonium chloride, and the pH value was adjusted to nine. Diethyl ether was added, and the mixture was transferred into a separatory funnel. Still under argon, the layers were separated, and the aqueous layer was extracted three times with diethyl ether. The combined organic layers were washed with water and brine, dried (magnesium sulfate), and the solvent was evaporated to provide 2-(3-methoxy-2-methylphenyl)ethanamine (5.33 g, 32.3 mmol, 92%) as a yellow oil. UV (MeOH): *λ* = 204, 218, 273, 280 nm; IR (ATR): *ν* = 3402, 2989, 2923, 2848, 2659, 2480, 2065, 1658, 1604, 1581, 1510, 1463, 1395, 1293, 1256, 1194, 1171, 1149, 1122, 1096, 1006, 953, 875, 789, 776, 763, 719 cm^–1^; ^1^H-NMR (600 MHz, CDCl_3_): *δ* = 2.22 (s, 3H), 3.10–3.19 (m, 4H), 3.82 (s, 3H), 6.77 (d, *J* = 8.2 Hz, 1H), 6.83 (d, *J* = 7.6 Hz, 1H), 7.13 (t, *J* = 7.9 Hz, 1H); ^13^C-NMR (150 MHz, methanol-*d*_4_): *δ* = 11.47 (CH_3_), 32.26 (CH_2_), 40.35 (CH_2_), 55.49 (CH_3_), 109.10 (CH), 121.76 (CH), 125.07 (C), 126.59 (CH), 135.84 (C), 158.03 (C); MS (ESI, +10 V): *m*/*z* = 149.0 [M − NH_3_ + H]^+^, 166.0 [M + H]^+^, 331.2 [2M + H]^+^; HRMS: calcd. for C_10_H_15_NO: 165.1153, found: 165.1144.

*2-(3-Methoxy-2-methylphenyl)ethyl acetamide* (**12**). DMAP (14 mg, 0.11 mmol) was added to a solution of 2-(3-methoxy-2-methylphenyl)ethanamine (230 mg, 1.14 mmol) in pyridine (4.5 mL), and the mixture was cooled to 0 °C. Acetic anhydride (140 µL, 15 mmol) was added dropwise over a period of five minutes, and the reaction mixture was stirred for 4 h. The solvent was evaporated, and the raw material was purified by chromatography (Alox N, 5% H_2_O; ethyl acetate) to provide 2-(3-methoxy-2-methylphenyl)ethyl acetamide (**12**, 235 mg, 1.13 mmol, 99%) as a light yellow solid. M.p. 84–85 °C; UV (MeOH): *λ* = 205, 219, 229, 271, 279 nm; IR (ATR): *ν* = 3267, 3085, 2932, 2836, 2030, 2009, 1976, 1716, 1659, 1630, 1564, 1508, 1489, 1472, 1459, 1435, 1396, 1370, 1298, 1285, 1247, 1201, 1180, 1110, 1092, 1037, 1013, 896, 812, 776, 748, 723, 701, 651, 606 cm^–1^; ^1^H-NMR (500 MHz, CDCl_3_): *δ* = 1.95 (s, 3H), 2.19 (s, 3H), 2.84 (t, *J* = 6.9 Hz, 2H), 3.46 (q, *J* = 6.9 Hz, 2H), 3.82 (s, 3H), 5.46 (br s, 1H), 6.76 (d, *J* = 7.9 Hz, 2H), 7.12 (t, *J* = 7.9 Hz, 1H); ^13^C-NMR (125 MHz, CDCl_3_): *δ* = 11.53 (CH_3_), 23.51 (CH_3_), 33.38 (CH_2_), 39.87 (CH_2_), 55.64 (CH_3_), 108.61 (CH), 121.89 (CH), 125.24 (C), 126.36 (CH), 138.38 (C), 158.09 (C), 170.21 (C=O); MS (ESI, +10 V): *m*/*z* = 208.0 [M + H]^+^, 415.1 [2M + H]^+^, 437.1 [2M + Na]^+^; HRMS: calcd. for C_12_H_17_NO_2_: 207.1259, found: 207.1248; elemental analysis: calcd. for C_12_H_17_NO_2_: C: 69.54, H: 8.27, N: 6.76; found: C: 69.04, H: 8.73, N: 6.78.

*6-Methoxy-1,5-dimethyl-3,4-dihydroisoquinoline.* Phosphorus oxychloride (1.9 mL, 21 mmol) was added to a refluxing solution of acetamide **12** (433 mg, 2.09 mmol) in freshly distilled chloroform (23 mL), and the mixture was stirred for one hour. Subsequently, solvent and excess phosphorus oxychloride were removed under vigorous stirring by a nitrogen stream through a pair of soda lye-filled gas washing bottles. The remaining oily raw product was dissolved in ethyl acetate. Soda lye (10%) was added, and the pH value was adjusted to 8–9 using saturated aqueous ammonium chloride. The layers were separated, and the aqueous layer was extracted with ethyl acetate. The combined organic layers were washed with water and brine, and then dried (magnesium sulfate), and the solvent was evaporated. Purification of the crude product by chromatography (Alox N, 5% H_2_O; ethyl acetate + 3% triethylamine) afforded 6-methoxy-1,5-dimethyl-3,4-dihydroisoquinoline (393 mg, 2.08 mmol, 99%) as a yellow solid. M.p. 57–58 °C (subl.); UV (MeOH): *λ* = 229, 274, 319 nm; IR (ATR): *ν* = 3002, 2939, 2838, 1735, 1699, 1629, 1594, 1576, 1539, 1507, 1482, 1435, 1368, 1291, 1258, 1184, 1149, 1101, 1015, 922, 901, 873, 805, 751, 700, 666, 637 cm^–1^; ^1^H-NMR (500 MHz, CDCl_3_): *δ* = 2.15 (s, 3H), 2.35 (t, *J* = 1.4 Hz, 3H), 2.64 (t, *J* = 7.4 Hz, 2H), 3.63 (tq, *J* = 7.4, 1.4 Hz, 2H), 3.85 (s, 3H), 6.75 (d, *J* = 8.5 Hz, 1H), 7.37 (d, *J* = 8.5 Hz, 1H); ^13^C-NMR (125 MHz, CDCl_3_): *δ* = 11.08 (CH_3_), 23.32 (CH_2_), 23.62 (CH_3_), 46.91 (CH_2_), 55.61 (CH_3_), 107.50 (CH), 123.06 (C), 123.40 (C), 124.76 (CH), 137.70 (C), 159.47 (C), 164.80 (C); MS (EI): *m*/*z* (%) = 189 (95, [M]^+^), 174 (100), 158 (16), 144 (23), 131 (22), 115 (31), 105 (23), 91 (22), 77 (29), 63 (17), 51 (20); HRMS: calcd. for C_12_H_15_NO: 189.1154, found: 189.1147; elemental analysis: calcd. for C_12_H_15_NO: C: 76.16, H: 7.99, N: 7.40; found: C: 76.25, H: 7.98, N: 7.46.

*6-Methoxy-1,5-dimethylisoquinoline* (**13**). A flask filled with 6-methoxy-1,5-dimethyl-3,4-dihydroisoquinoline (90.3 mg, 0.48 mmol) and palladium on charcoal (10%, 93.6 mg) was evacuated under vigorous stirring for 15 min, and then filled with argon. Toluene (3.6 mL) and cyclohexene (1.3 mL, 13 mmol) were added, and the mixture was heated at reflux until full conversion was detected (TLC: Alox N; ethyl acetate/isohexane, 2:1 + 1 drop of ethanol). The catalyst was removed by filtration (ethyl acetate), and the crude product was purified by chromatography (Alox N, 5% H_2_O; petroleum ether/ethyl acetate, 5:1) to provide 6-methoxy-1,5-dimethylisoquinoline (**13**, 90 mg, 0.48 mmol, 100%) as a beige solid. M.p. 99–102 °C; UV (MeOH): *λ* = 203, 236, 301 nm; IR (ATR): *ν* = 3058, 3015, 2965, 2940, 2847, 1608, 1563, 1542, 1495, 1457, 1401, 1344, 1324, 1267, 1179, 1153, 1116, 1078, 1009, 984, 913, 848, 814, 774, 698, 673, 648, 581, 528 cm^–1^; ^1^H-NMR (600 MHz, CDCl_3_): *δ* = 2.50 (s, 3H), 2.97 (s, 3H), 4.00 (s, 3H), 7.35 (d, *J* = 9.2 Hz, 1H), 7.63 (d, *J* = 6.2 Hz, 1H), 8.05 (d, *J* = 9.2 Hz, 1H), 8.32 (d, *J* = 6.2 Hz, 1H); ^13^C-NMR (150 MHz, CDCl_3_): *δ* = 10.49 (CH_3_), 22.17 (CH_3_), 56.39 (CH_3_), 113.71 (CH), 115.83 (CH), 118.97 (C), 123.04 (C), 125.66 (CH), 127.47 (C), 136.96 (C), 140.72 (CH), 158.57 (C); MS (EI): *m*/*z* (%) = 187 (100, [M]^+^), 172 (26), 156 (16), 144 (80), 128 (19), 115 (43), 103 (21), 89 (11), 77 (30), 63 (24), 51 (22); HRMS: calcd. for C_12_H_13_NO: 187.0997, found: 187.0986; elemental analysis: calcd. for C_12_H_13_NO: C: 76.98, H: 7.00, N: 7.48; found: C: 76.43, H: 7.04, N: 7.53.

*1,5-Dimethylisoquinolin-6-ol. For small amounts:* In a microwave tube, a mixture of 6-methoxy-1,5-dimethylisoquinoline (**13**, 45 mg, 0.24 mmol) and pyridinium chloride (1 g, 8 mmol) was irradiated at 155 °C (300 W) for 30 min. After cooling to room temperature, the mixture was dissolved in water and ethyl acetate, and neutralized with a saturated aqueous solution of sodium bicarbonate. The layers were separated, and the aqueous layer was carefully extracted with ethyl acetate. The combined organic layers were dried (magnesium sulfate), and the solvent was evaporated to give 1,5-dimethylisoquinolin-6-ol (40 mg, 0.23 mmol, 96%) as a brownish solid.

*For larger amounts:* Freshly distilled hydrobromic acid (22 mL, 0.19 mol) was carefully added at 0 °C to 6-methoxy-1,5-dimethylisoquinoline (**13**, 3.01 g, 16.1 mmol). After the addition was completed, the cooling bath was removed, and the mixture was heated at reflux for five hours. Then, the excess of hydrobromic acid was removed under vacuo. The brownish raw material was completely dissolved in water (115 mL, ultrasound), filtered, and neutralized by the dropwise addition of a saturated aqueous solution of sodium bicarbonate. The resulting solid was carefully washed with water and dried in vacuo to provide 1,5-dimethylisoquinolin-6-ol (2.49 g, 14.4 mmol, 89%) as a brownish solid. M.p. 248–250 °C (sublimation); UV (MeOH): *λ* = 234, 279, 301, 328, 382 nm; IR (ATR): *ν* = 2920, 2850, 2475 (br), 1808 (br), 1617, 1599, 1564, 1479, 1423, 1385, 1356, 1337, 1279, 1202, 1057, 1006, 939, 813, 774, 718, 672, 660 cm^–1^; ^1^H-NMR (500 MHz, methanol-*d*_4_): *δ* = 2.43 (s, 3H), 2.84 (s, 3H), 7.22 (d, *J* = 9.1 Hz, 1H), 7.64 (d, *J* = 6.2 Hz, 1H), 7.95 (d, *J* = 9.1 Hz, 1H), 8.12 (d, *J* = 6.2 Hz, 1H); ^13^C-NMR (125 MHz, methanol-*d*_4_): *δ* = 10.13 (CH_3_), 21.33 (CH_3_), 116.26 (C), 116.72 (CH), 119.91 (CH), 123.50 (C), 126.19 (CH), 138.79 (C), 140.60 (CH), 157.91 (C), 158.92 (C); MS (ESI, +10 V): *m*/*z* = 174.0 [M + H]^+^; HRMS: calcd. for C_11_H_11_NO: 173.0841, found: 173.0851; elemental analysis: calcd. for C_11_H_11_NO: C: 76.28, H: 6.40, N: 8.09; found: C: 76.00, H: 6.47, N: 8.21.

*1,5-Dimethylisoquinolin-6-yl trifluoromethanesulfonate* (**14**). Pyridine (1.1 mL, 12 mmol) was added to a suspension of 1,5-dimethylisoquinolin-6-ol (0.60 g, 3.5 mmol) in acetonitrile (66 mL) at 0 °C. Subsequently, trifluoromethanesulfonic anhydride (0.87 mL, 5.2 mmol) was added dropwise, and the reaction mixture was stirred at this temperature for 20 h. Ethyl acetate and water were added, and the layers were separated. The aqueous layer was extracted three times with ethyl acetate. The combined organic layers were washed with water and brine, and then dried (sodium sulfate). The solvent was evaporated, and the residue was purified by column chromatography (silica gel, pentane/ethyl acetate, 1:1) to provide 1,5-dimethylisoquinolin-6-yl trifluoromethanesulfonate (**14**, 0.92 g, 3.0 mmol, 87%) as a beige solid. M.p. 67–67.5 °C; UV (MeOH): *λ* = 198, 219, 274, 308, 321 nm; IR (ATR): *ν* = 3088, 3031, 2995, 2927, 2856, 1612, 1564, 1522, 1473, 1459, 1414, 1375, 1350, 1245, 1207, 1170, 1132, 1038, 994, 933, 861, 826, 815, 767, 663, 621 cm^–1^; ^1^H-NMR (500 MHz, CDCl_3_): *δ* = 2.68 (s, 3H), 3.00 (s, 3H), 7.48 (d, *J* = 9.3 Hz, 1H), 7.71 (d, *J* = 6.1 Hz, 1H), 8.10 (d, *J* = 9.3 Hz, 1H), 8.52 (d, *J* = 6.1 Hz, 1H); ^13^C-NMR (125 MHz, CDCl_3_): *δ* = 12.24 (CH_3_), 22.88 (CH_3_), 116.23 (CH), 118.77 (q, *J_C,F_* = 321 Hz, CF_3_), 120.75 (CH), 126.42 (CH and C), 126.64 (C), 136.97 (C), 143.40 (CH), 147.91 (C), 159.50 (C); ^19^F-NMR (282 MHz, CDCl_3_): *δ* = −73.58 (s, 3F); MS (EI): *m*/*z* (%) = 305 (1, [M]^+^), 172 (8), 144 (48), 128 (7), 115 (19), 103 (13), 89 (5), 77 (18), 69 (100), 63 (10), 51 (12); MS (ESI, +10 V): *m*/*z* = 306.0 [M + H]^+^; elemental analysis: calcd. for C_12_H_10_F_3_NO_3_S: C: 47.21, H: 3.30, N: 4.59, S: 10.50; found: C: 47.09, H: 3.02, N: 4.58, S: 10.45.

*1,5-Dimethyl-N-phenylisoquinolin-6-amine* (**16**). Aniline (**15**, 0.1 mL, 1.2 mmol) was added dropwise to a solution of 1,5-dimethylisoquinolin-6-yl trifluoromethanesulfonate (**14**, 0.235 g, 0.770 mmol), palladium(II) acetate (13 mg, 58 µmol), XPhos (55 mg, 0.12 mmol), and cesium carbonate (0.35 g, 1.1 mmol) in toluene (20 mL). The mixture was heated at reflux for 48 h. After cooling to room temperature, the reaction mixture was filtered over a short pad of Hyflo (ethyl acetate), and the solvent was evaporated. Purification of the residue by column chromatography (silica gel, dichloromethane/ethyl acetate 1:3 + 1% methanol) provided 1,5-dimethyl-*N*-phenylisoquinolin-6-amine (**16**, 0.19 g, 0.77 mmol, 100%) as a yellow solid. M.p. 175 °C (decomp.); UV (MeOH): *λ* = 223, 250, 280, 325, 358 (sh) nm; IR (ATR): *ν* = 3207, 3163, 3090, 3012, 2985, 2919, 2860, 1632, 1615, 1594, 1562, 1526, 1492, 1439, 1397, 1380, 1310, 1286, 1174, 1151, 1060, 990, 938, 864, 844, 819, 788, 748, 695, 678 cm^–1^; ^1^H-NMR (500 MHz, CDCl_3_): *δ* = 2.50 (s, 3H), 2.91 (s, 3H), 5.83 (br s, 1H), 7.01 (t, *J* = 7.4 Hz, 1H), 7.05 (d, *J* = 7.7 Hz, 2H), 7.29–7.33 (m, 2H), 7.53 (d, *J* = 9.1 Hz, 1H), 7.60 (d, *J* = 6.1 Hz, 1H), 7.92 (d, *J* = 9.1 Hz, 1H), 8.35 (d, *J* = 6.1 Hz, 1H); ^13^C-NMR (125 MHz, CDCl_3_): *δ* = 12.38 (CH_3_), 22.66 (CH_3_), 115.32 (CH), 118.78 (C), 118.88 (2 CH), 119.96 (CH), 121.99 (CH), 123.77 (C), 124.80 (CH), 129.63 (2 CH), 136.93 (C), 141.95 (C), 142.27 (CH), 142.91 (C), 158.59 (C); MS (EI): *m*/*z* (%) = 248 (100, [M]^+^), 233 (16), 171 (17); MS (ESI, +10 V): *m*/*z* = 249.1 [M + H]^+^; HRMS (ESI): calcd. for C_17_H_16_N_2_: 248.1313, found: 248.1310.

*N-(2-Chlorophenyl)-1,5-dimethylisoquinolin-6-amine* (**18a**). 2-Chloroaniline (**17a**, 78 µL, 0.74 mmol) was added dropwise to a solution of 1,5-dimethylisoquinolin-6-yl trifluoromethanesulfonate (**14**, 0.15 g, 0.49 mmol), palladium(II) acetate (8.3 mg, 37 µmol), XPhos (35 mg, 74 µmol), and cesium carbonate (224 mg, 0.688 mmol) in toluene (12 mL). The mixture was heated at reflux for 1.5 h. After cooling to room temperature, the reaction mixture was filtered over a short pad of Hyflo (ethyl acetate), and the solvent was evaporated. Purification of the residue by column chromatography (silica gel, dichloromethane/ethyl acetate, 9:1 to 0:1, each + 1% ethanol) provided *N*-(2-chlorophenyl)-1,5-dimethylisoquinolin-6-amine (**18a**, 0.130 g, 0.460 mmol, 94%) as brownish crystals. M.p. 194–198 °C; UV (MeOH): *λ* = 221, 249, 278, 320 nm; fluorescence (MeOH): *λ*_ex_ = 221, *λ*_em_ = 229 (sh), 334 nm; IR (ATR): *ν* = 3189, 3078, 2955, 2919, 2850, 1589, 1567, 1542, 1501, 1474, 1452, 1396, 1367, 1307, 1294, 1267, 1225, 1198, 1129, 1058, 1033, 996, 933, 862, 843, 822, 793, 751, 706 cm^–1^; ^1^H-NMR (500 MHz, CDCl_3_): *δ* = 2.35 (s, 3H), 2.93 (s, 3H), 6.15 (br s, 1H), 6.85 (td, *J* = 7.7, 3.0 Hz, 1H), 6.95 (dd, *J* = 8.2, 1.4 Hz, 1H), 7.10–7.14 (m, 1H), 7.40 (dd, *J* = 8.0, 1.4 Hz, 1H), 7.51 (d, *J* = 9.0 Hz, 1H), 7.63 (d, *J* = 6.1 Hz, 1H), 7.96 (d, *J* = 9.0 Hz, 1H), 8.39 (d, *J* = 6.1 Hz, 1H); ^13^C-NMR (125 MHz, CDCl_3_): *δ* = 12.58 (CH_3_), 22.58 (CH_3_), 115.47 (CH), 116.05 (CH), 120.90 (CH), 121.87 (C), 122.07 (CH), 122.83 (C), 124.52 (C), 124.73 (CH), 127.51 (CH), 129.80 (CH), 136.78 (C), 140.07 (C), 140.19 (C), 142.35 (CH), 158.61 (C); MS (EI): *m*/*z* (%) = 282 (100, [M]^+^), 247 (74), 232 (29), 204 (15), 171 (12), 115 (10), 75 (11); MS (ESI, +25 V): *m*/*z* = 283.2 [M + H]^+^.

Crystal data: C_17_H_15_ClN_2_, crystal size 0.22 × 0.20 × 0.06 mm^3^, *M* = 282.76 g mol^−1^, monoclinic, space group: *Cc*, *a* = 11.700(2), *b* = 9.117(2), *c* = 14.024(3) Å, *β* = 110.73(3)°, *V* = 1399.1(5) Å^3^, *Z* = 4, *ρ_calcd._* = 1.342 g cm^−3^, *µ* = 0.264 mm^−1^, *T* = 198(2) K, *λ* = 0.71073 Å, *θ* range: 3.11–27.00°, 20817 reflections collected, 3047 independent (*R*_int_ = 0.0534), 187 parameters. The structure was solved by direct methods and refined by the full-matrix least-squares method on *F*^2^; 2296 reflections observed, *R*_1_ = 0.0407, *wR*_2_ = 0.0805 [*I* > 2 *σ*(*I*)]; maximal residual electron density: 0.276 e Å^−3^. CCDC 1838728.

*N-(2-Chloro-5-methoxyphenyl)-1,5-dimethylisoquinolin-6-amine* (**18b**). 2-Chloro-5-methoxyaniline (**17b**, 92 µL, 0.74 mmol) was added dropwise to a solution of 1,5-dimethylisoquinolin-6-yl trifluoromethanesulfonate (**14**, 0.15 g, 0.49 mmol), palladium(II) acetate (8.3 mg, 37 µmol), XPhos (35 mg, 74 µmol), and cesium carbonate (224 mg, 0.688 mmol) in toluene (12 mL). The mixture was heated at reflux for five hours. After cooling to room temperature, the reaction mixture was filtered over a short pad of Hyflo (ethyl acetate), and the solvent was evaporated. Purification of the residue by column chromatography (silica gel, dichloromethane/ethyl acetate, 1:1 to 0:1, each + 1% ethanol) provided *N*-(2-chloro-5-methoxyphenyl)-1,5-dimethylisoquinolin-6-amine (**18b**, 0.141 g, 0.451 mmol, 92%) as a beige solid. M.p. 135–138 °C; UV (MeOH): *λ* = 224, 277, 322 nm; fluorescence (MeOH): *λ*_ex_ = 224, *λ*_em_ = 301 (sh), 336 nm; IR (ATR): *ν* = 3416, 3068, 2998, 2929, 2853, 1596, 1508, 1447, 1421, 1383, 1343, 1312, 1287, 1230, 1207, 1170, 1138, 1069, 1027, 924, 820, 732, 671, 640 cm^–1^; ^1^H-NMR (500 MHz, CDCl_3_): *δ* = 2.53 (s, 3H), 2.94 (s, 3H), 3.68 (s, 3H), 6.13 (br s, 1H), 6.40 (dd, *J* = 8.8, 2.8 Hz, 1H), 6.47 (d, *J* = 2.8 Hz, 1H), 7.28 (d, *J* = 8.8 Hz, 1H), 7.53 (d, *J* = 9.0 Hz, 1H), 7.63 (d, *J* = 6.1 Hz, 1H), 7.97 (d, *J* = 9.0 Hz, 1H), 8.39 (d, *J* = 6.1 Hz, 1H); ^13^C-NMR (125 MHz, CDCl_3_): *δ* = 12.66 (CH_3_), 22.61 (CH_3_), 55.46 (CH_3_), 101.82 (CH), 105.94 (CH), 113.40 (C), 115.53 (CH), 122.55 (CH), 123.53 (C), 124.67 (C), 124.76 (CH), 130.02 (CH), 136.78 (C), 139.77 (C), 141.06 (CH), 142.38 (C), 158.64 (C), 159.20 (C); MS (EI): *m*/*z* (%) = 312 (100, [M]^+^), 277 (80), 262 (76), 247 (13), 233 (18), 219 (12), 139 (10), 117 (16), 63 (10); MS (ESI, +10 V): *m*/*z* = 313.3 [M + H]^+^.

*N-(2-Chloro-4-methoxyphenyl)-1,5-dimethylisoquinolin-6-amine* (**18c**). A solution of 2-chloro-4-methoxyaniline (**17c**, 127 mg, 0.806 mmol) in toluene (4 mL) was added dropwise to a solution of 1,5-dimethylisoquinolin-6-yl trifluoromethanesulfonate (**14**, 164 mg, 0.537 mmol), palladium(II) acetate (9 mg, 0.04 mmol), XPhos (38 mg, 81 µmol), and cesium carbonate (245 mg, 0.752 mmol) in toluene (10 mL). The mixture was heated at reflux for one hour. After cooling to room temperature, the reaction mixture was filtered over a short pad of Hyflo (ethyl acetate), and the solvent was evaporated. Purification of the residue by column chromatography (silica gel, dichloromethane/ethyl acetate, 9:1 to 0:1, each + 1% ethanol) provided *N*-(2-chloro-4-methoxyphenyl)-1,5-dimethylisoquinolin-6-amine (**18c**, 139 mg, 0.444 mmol, 83%) as a beige solid. M.p. 104–107 °C; UV (MeOH): *λ* = 226, 255, 318 nm; fluorescence (MeOH): *λ*_ex_ = 255, *λ*_em_ = 422 nm; IR (ATR): *ν* = 3229, 3074, 2993, 2948, 2832, 1731, 1633, 1606, 1562, 1485, 1451, 1436, 1387, 1341, 1308, 1283, 1211, 1182, 1112, 1046, 936, 894, 864, 822, 789, 773, 689, 664 cm^–1^; ^1^H-NMR (500 MHz, CDCl_3_): *δ* = 2.51 (s, 3H), 2.93 (s, 3H), 3.80 (s, 3H), 5.87 (br s, 1H), 6.79 (dd, *J* = 8.7, 2.8 Hz, 1H), 7.02 (d, *J* = 2.8 Hz, 1H), 7.09 (d, *J* = 8.9 Hz, 1H), 7.29 (d, *J* = 9.1 Hz, 1H), 7.62 (d, *J* = 6.2 Hz, 1H), 7.90 (d, *J* = 9.1 Hz, 1H), 8.31 (d, *J* = 6.2 Hz, 1H); ^13^C-NMR (125 MHz, CDCl_3_): *δ* = 12.01 (CH_3_), 21.92 (CH_3_), 55.84 (CH_3_), 113.76 (CH), 115.29 (CH), 115.33 (CH), 117.69 (C), 118.89 (CH), 122.01 (CH), 123.18 (C), 124.98 (CH), 126.33 (C), 132.26 (C), 136.87 (C), 140.79 (CH, HSQC), 142.90 (C, HMBC), 155.61 (C), 158.06 (C); MS (EI): *m*/*z* (%) = 312 (100, [M]^+^), 297 (44), 277 (12), 262 (14), 233 (17), 169 (12), 155 (11), 128 (14), 116 (15); MS (ESI, +10 V): *m*/*z* = 313.2 [M + H]^+^; elemental analysis: calcd. for C_18_H_17_ClN_2_O: C: 69.12, H: 5.48, N: 8.96; found: C: 68.62, H: 5.72, N: 9.30.

*Olivacine* (**1**). *N*-(2-Chlorophenyl)-1,5-dimethylisoquinolin-6-amine (**18a**, 20 mg, 71 µmol), palladium(II) acetate (4.8 mg, 21 µmol), tri-*tert*-butylphosphonium tetrafluoroborate (8.1 mg, 42 µmol), and potassium carbonate (39.1 mg, 0.283 mmol) were dissolved in DMF (0.5 mL). The reaction mixture was placed in a preheated oil bath at 140 °C and stirred for 30 min. After filtration over a short pad of Celite (CH_2_Cl_2_), the halogenated solvent was evaporated, and the residue was dissolved in ethyl acetate, and washed three times with water, and then with brine. The aqueous layer was extracted with ethyl acetate, and the combined organic layers were dried (sodium sulfate). The solvent was evaporated, and the residue was purified by column chromatography (silica gel, dichloromethane/ethyl acetate, 9:1 to 0:1, each + 5% ethanol) to provide olivacine (**1**, 12.4 mg, 50.3 µmol, 71%) as brown crystals. M.p. 320–324 °C; UV (MeOH): *λ* = 223, 237, 275, 285, 292, 327, 342, 374, 391 nm; fluorescence (MeOH): *λ*_ex_ = 285, *λ*_em_ = 431 nm; IR (ATR): *ν* = 3058, 2965, 2909, 2765, 1674, 1597, 1479, 1467, 1407, 1334, 1311, 1280, 1252, 1222, 1196, 1150, 1108, 1064, 942, 862, 813, 765, 739, 695, 640 cm^–1^; ^1^H-NMR (500 MHz, methanol-*d*_4_): *δ* = 2.85 (s, 3H), 3.07 (t, *J*_HD_ = 1.1 Hz) and 3.09 (s, 3H), 7.24–7.27 (m, 1H), 7.49–7.54 (m, 2H), 7.89 (d, *J* = 6.3 Hz, 1H), 8.18 (d, *J* = 6.3 Hz, 1H), 8.27–8.29 (m, 1H), 8.87 (s, 1H); ^13^C-NMR (125 MHz, methanol-*d*_4_): *δ* = 12.42 (CH_3_), 22.35 (CH_3_), 111.86 (CH), 112.42 (C), 116.05 (CH), 116.64 (CH), 120.58 (CH), 122.14 (CH), 123.57 (C), 124.41 (C), 127.25 (C), 128.93 (CH), 134.41 (C), 138.84 (CH), 142.80 (C), 144.34 (C), 160.29 (C); MS (EI): *m*/*z* (%) = 246 (100, [M]^+^), 229 (7), 217 (7), 204 (9), 123 (7); MS (ESI, +10 V): *m*/*z* = 247.1 [M + H]^+^; HRMS (ESI): calcd. for C_17_H_14_N_2_: 246.1157, found: 246.11537; elemental analysis: calcd. for C_17_H_14_N_2_: C: 82.90, H: 5.73, N: 11.37; found: C: 83.20, H: 5.81, N: 11.42.

Crystal data: C_17_H_14_N_2_·CH_3_OH, crystal size 0.45 × 0.12 × 0.07 mm^3^, *M* = 278.34 g mol^−1^, orthorhombic, space group: *Pbca*, *a* = 4.860(1), *b* = 21.337(5), *c* = 28.048(6) Å, *V* = 2908.5(11) Å^3^, *Z* = 8, *ρ_calcd._* = 1.271 g cm^−3^, *µ* = 0.080 mm^−1^, *T* = 198(2) K, *λ* = 0.71073 Å, *θ* range: 3.48–25.40°, 60682 reflections collected, 2656 independent (*R*_int_ = 0.0501), 198 parameters. The structure was solved by direct methods and refined by the full-matrix least-squares method on *F*^2^; 1934 reflections observed, *R*_1_ = 0.0463, *wR*_2_ = 0.1044 [*I* > 2 *σ*(*I*)]; maximal residual electron density: 0.204 e Å^−3^. CCDC 1838729.

*4,11b-Dimethyl-11bH-pyrido[3,4-c]carbazole* (**20a**, 2.1 mg, 8.5 µmol, 12%), dark brown oil, less polar side product. UV (MeOH): *λ* = 250, 282 (sh), 359 nm; fluorescence (MeOH): *λ*_ex_ = 250, *λ*_em_ = 417 nm; IR (ATR): *ν* = 3348, 2924, 2853, 2487, 1630, 1594, 1545, 1446, 1200, 1116, 950, 811, 772, 749, 679 cm^–1^; ^1^H-NMR (600 MHz, methanol-*d*_4_): *δ* = 1.65 (s, 3H), 2.76 (s, 3H), 7.01 (d, *J* = 10.0 Hz, 1H), 7.49 (t, *J* = 7.4 Hz, 1H), 7.55 (t, *J* = 7.5 Hz, 1H), 7.63 (d, *J* = 10.0 Hz, 1H), 7.70 (d, *J* = 7.6 Hz, 1H), 7.84 (d, *J* = 5.1 Hz, 1H), 8.09 (d, *J* = 7.3 Hz, 1H), 8.40 (d, *J* = 5.1 Hz, 1H); ^13^C-NMR (150 MHz, methanol-*d*_4_): *δ* = 21.70 (CH_3_), 33.28 (CH_3_), 59.3 (C, HMBC), 119.63 (CH), 122.46 (CH), 123.20 (CH), 125.25 (CH), 127.3 (C, HMBC), 127.89 (CH), 129.93 (CH), 136.11 (CH), 140.89 (C), 149.15 (CH), 153.38 (C), 155.0 (C, HMBC), 158.0 (C, HMBC), 184.5 (C, HMBC); MS (EI): *m*/*z* (%) = 246 (100, [M]^+^), 231 (24), 204 (12), 176 (7); MS (ESI, +50 V): *m*/*z* = 247.1 [M + H]^+^, 493.5 [2M + H]^+^.

*8-Methoxyolivacine* (**19b**). *N*-(2-Chloro-5-methoxyphenyl)-1,5-dimethylisoquinolin-6-amine (**18b**, 14 mg, 45 µmol), palladium(II) acetate (3.0 mg, 13 µmol), tri-*tert*-butylphosphonium tetrafluoroborate (5.1 mg, 27 µmol) and potassium carbonate (24.7 mg, 0.179 mmol) were dissolved in DMF (0.5 mL). The reaction mixture was placed in a preheated oil bath at 140 °C and stirred for 20 min. After filtration over a short pad of Celite (CH_2_Cl_2_), the halogenated solvent was evaporated, and the residue was dissolved in ethyl acetate, washed three times with water, and then with brine. The aqueous layer was extracted with ethyl acetate, and the combined organic layers were dried (sodium sulfate). The solvent was evaporated and the residue was purified by column chromatography (silica gel, dichloromethane/ethyl acetate, 9:1 to 0:1, each + 5% ethanol) to provide 8-methoxyolivacine (**19b**, 8.0 mg, 29 µmol, 65%) as yellow crystals. M.p. 280–283 °C; UV (MeOH): *λ* = 227, 271, 281, 300, 316, 351 nm; fluorescence (MeOH): *λ*_ex_ = 300, *λ*_em_ = 430, 515 nm; IR (ATR): *ν* = 3141, 3046, 2993, 2886, 2821, 2713, 1622, 1595, 1563, 1493, 1472, 1460, 1412, 1388, 1335, 1315, 1297, 1267, 1216, 1197, 1160, 1137, 1099, 1068, 1030, 996, 942, 916, 870, 810, 753 cm^–1^; ^1^H-NMR (500 MHz, DMSO-*d*_6_): *δ* = 2.79 (s, 3H), 3.01 (s, 3H), 3.89 (s, 3H), 6.85 (dd, *J* = 8.6, 2.2 Hz, 1H), 7.00 (d, *J* = 2.2 Hz, 1 H), 7.78 (d, *J* = 6.1 Hz, 1H), 8.23 (d, *J* = 6.1 Hz, 1H), 8.24 (d, *J* = 8.6 Hz, 1 H), 8.77 (s, 1H), 11.26 (s, 1H); ^13^C-NMR (125 MHz, DMSO-*d*_6_): *δ* = 12.36 (CH_3_), 22.97 (CH_3_), 55.32 (CH_3_), 94.84 (CH), 107.55 (CH), 110.68 (C), 113.55 (CH), 114.78 (CH), 116.19 (C), 122.00 (C), 122.28 (CH), 125.00 (C), 131.74 (C), 139.10 (CH), 140.79 (C), 144.13 (C), 158.26 (C), 159.96 (C); MS (EI): *m*/*z* (%) = 276 (100, [M]^+^), 261 (14), 233 (49), 138 (8), 116 (10); MS (ESI, +10 V): *m*/*z* = 277.1 [M + H]^+^; HRMS (ESI): calcd. for C_18_H_16_N_2_O: 276.1263, found: 276.1261.

Crystal data: C_18_H_16_N_2_O·CH_3_OH, crystal size 0.160 × 0.080 × 0.060 mm^3^, *M* = 308.37 g mol^−1^, orthorhombic, space group: *Pbca*, *a* = 4.9253(3), *b* = 21.4925(15), *c* = 29.523(2) Å, *V* = 3125.3(4) Å^3^, *Z* = 8, *ρ_calcd._* = 1.311 g cm^−3^, *µ* = 0.685 mm^−1^, *T* = 150(2) K, *λ* = 1.54178 Å, *θ* range: 2.993–68.188°, 31382 reflections collected, 2811 independent (*R*_int_ = 0.0544), 230 parameters. The structure was solved by direct methods and refined by the full-matrix least-squares method on *F*^2^; 2283 reflections observed, *R*_1_ = 0.0387, *wR*_2_ = 0.1001 [*I* > 2 *σ*(*I*)]; maximal residual electron density: 0.221 e Å^−3^. CCDC 1838730.

*9-Methoxy-4,11b-dimethyl-11bH-pyrido[3,4-c]carbazole* (**20b**, 1.1 mg, 4.0 µmol, 9%), brown oil, less polar side product. UV (MeOH): *λ* = 221, 300, 325 nm; fluorescence (MeOH): *λ*_ex_ = 221, *λ*_em_ = 296, 339 nm; IR (ATR): *ν* = 3414, 3058, 2924, 2855, 1734, 1655, 1632, 1593, 1535, 1484, 1459, 1437, 1377, 1334, 1276, 1231, 1182, 1149, 1129, 1074, 935, 826, 740, 683 cm^–1^; ^1^H-NMR (600 MHz, methanol-*d*_4_): *δ* = 1.62 (s, 3H), 2.74 (s, 3H), 3.94 (s, 3H), 6.98 (d, *J* = 10.0 Hz, 1H), 7.04 (dd, *J* = 8.2, 1.7 Hz, 1H), 7.26 (s, 1H), 7.61 (d, *J* = 10.0 Hz, 1H), 7.78 (d, *J* = 4.9 Hz, 1H), 7.94 (d, *J* = 8.2 Hz, 1H), 8.38 (d, *J* = 4.9 Hz, 1H); ^13^C-NMR (150 MHz, methanol-*d*_4_): *δ* = 21.67 (CH_3_), 33.43 (CH_3_), 56.12 (CH_3_), 58.80 (C), 108.13 (CH), 113.67 (CH), 119.73 (CH), 123.16 (CH), 125.46 (CH), 127.23 (C), 132.78 (C, HMBC), 136.16 (CH), 149.15 (CH), 153.81 (C), 156.57 (C, HMBC), 158.04 (C), 162.22 (C), 186.18 (C); MS (EI): *m*/*z* (%) = 276 (85, [M]^+^), 261 (100), 233 (25), 218 (52), 190 (16); MS (ESI, +50 V): *m*/*z* = 277.2 [M + H]^+^.

*9-Methoxyolivacine* (**19c**). *N*-(2-Chloro-4-methoxyphenyl)-1,5-dimethylisoquinolin-6-amine (**18c**, 55.0 mg, 176 µmol), palladium(II) acetate (11.8 mg, 53 µmol), tri-*tert*-butylphosphonium tetrafluoroborate (20.1 mg, 106 µmol), and potassium carbonate (97.2 mg, 0.703 mmol) were dissolved in DMF (1.4 mL). The reaction mixture was placed in a preheated oil bath at 140 °C and stirred for 35 min. After filtration over a short pad of Celite (CH_2_Cl_2_), the halogenated solvent was evaporated, and the residue was dissolved in ethyl acetate, and then washed three times with water, and then with brine. The aqueous layer was extracted with ethyl acetate, and the combined organic layers were dried (sodium sulfate). The solvent was evaporated, and the residue was purified by column chromatography (silica gel, dichloromethane/ethyl acetate, 9:1 to 0:1, each + 5% ethanol) to provide 9-methoxyolivacine (**19c**, 30.1 mg, 109 µmol, 62%) as a yellow solid. M.p. 273–274 °C; UV (MeOH): *λ* = 224, 242, 272, 296, 332, 394 nm; fluorescence (MeOH): *λ*_ex_ = 296, *λ*_em_ = 471 nm; IR (ATR): *ν* = 3143, 2914, 1632, 1600, 1485, 1436, 1405, 1380, 1330, 1306, 1265, 1206, 1175, 1104, 1030, 935, 879, 862, 838, 809, 767, 735, 698 cm^–1^; ^1^H-NMR (500 MHz, DMSO-*d*_6_): *δ* = 2.80 (s, 3H), 3.04 (s, 3H), 3.90 (s, 3H), 7.14 (dd, *J* = 10.0, 2.5 Hz, 1H), 7.44 (d, *J* = 8.7 Hz, 1H), 7.79 (d, *J* = 6.1 Hz, 1H), 8.01 (d, *J* = 2.5 Hz, 1H), 8.24 (d, *J* = 6.1 Hz, 1H), 8.96 (s, 1H), 11.16 (s, 1H); ^13^C-NMR (125 MHz, DMSO-*d*_6_): *δ* = 12.80 (CH_3_), 23.45 (CH_3_), 56.11 (CH_3_), 104.87 (CH), 111.36 (C), 112.00 (CH), 115.18 (CH), 115.71 (CH), 117.04 (CH), 122.00 (C), 123.65 (C), 125.36 (C), 132.64 (C), 137.53 (C), 139.69 (CH), 141.61 (C), 153.78 (C), 159.21 (C); MS (EI): *m*/*z* (%) = 276 (100, [M]^+^), 261 (90), 233 (27), 116 (10); MS (ESI, +10 V): *m*/*z* = 277.1 [M + H]^+^; HRMS (ESI): calcd. for C_18_H_16_N_2_O: 276.1263, found: 276.1269.

*10-Methoxy-4,11b-dimethyl-11bH-pyrido[3,4-c]carbazole* (**20c**, 1.4 mg, 5.0 µmol, 3%), brown oil, less polar side product. UV (MeOH): *λ* = 260, 291 (sh), 381 nm; fluorescence (MeOH): *λ*_ex_ = 260, *λ*_em_ = 349 (sh), 434 nm; IR (ATR): *ν* = 3389, 2924, 2854, 1733, 1655, 1624, 1590, 1536, 1466, 1434, 1380, 1335, 1295, 1275, 1240, 1218, 1165, 1065, 1030, 952, 865, 822, 744, 677 cm^–1^; ^1^H-NMR (600 MHz, methanol-*d*_4_): *δ* = 1.63 (s, 3H), 2.74 (s, 3H), 4.00 (s, 3H), 6.95 (d, *J* = 10.0 Hz, 1H), 7.10 (dd, *J* = 8.5, 2.1 Hz, 1H), 7.55 (d, *J* = 10.0 Hz, 1H), 7.60 (d, *J* = 8.5 Hz, 1H), 7.66 (d, *J* = 2.1 Hz, 1H), 7.83 (d, *J* = 5.1 Hz, 1H), 8.38 (d, *J* = 5.1 Hz, 1H); ^13^C-NMR (150 MHz, methanol-*d*_4_): *δ* = 21.69 (CH_3_), 33.33 (CH_3_), 56.44 (CH_3_), 59.25 (C), 112.13 (CH), 114.60 (CH), 119.52 (CH), 122.93 (CH), 123.26 (CH), 127.5 (C, HMBC), 134.87 (CH), 142.7 (C, HMBC), 147.3 (C, HMBC), 148.89 (CH), 153.2 (C, HMBC), 157.8 (C, HMBC), 160.73 (C), 182.4 (C, HMBC); MS (EI): *m*/*z* (%) = 276 (100, [M]^+^), 261 (42), 246 (24), 233 (46), 218 (31), 190 (13); MS (ESI, +50 V): *m*/*z* = 277.2 [M + H]^+^.

*8-Hydroxyolivacine* (**4**). 8-Methoxyolivacine (**19b**, 17.0 mg, 61.5 µmol) was dissolved in 48% aqueous HBr (1.1 mL), and the mixture was heated at reflux for 24 h. After cooling to room temperature, the mixture was carefully neutralized using a 25% aqueous solution of ammonia. The mixture was extracted with ethyl acetate until the aqueous layer was completely colorless. Evaporation of the organic solvent led to a yellow solid, which was purified by chromatography (Alox N, 5% H_2_O, CH_2_Cl_2_/methanol, 1:1) to provide 8-hydroxyolivacine (**4**, 13.5 mg, 51.5 µmol, 84%) as a yellow solid. An additional purification by preparative HPLC provided very pure **4** (8.5 mg, 32 µmol) for biological testing. M.p. 239 °C; UV (MeOH): *λ* = 239, 301, 317 nm; fluorescence (MeOH): *λ*_ex_ = 301, *λ*_em_ = 434, 520 nm; IR (ATR): *ν* = 3505, 3279, 3198, 2827, 1660, 1619, 1474, 1433, 1407, 1341, 1190, 1166, 1138, 1102, 840, 800, 722, 633 cm^–1^; ^1^H-NMR (500 MHz, methanol-*d*_4_): *δ* = 2.94 (s, 3H), 3.34 (s, 3H), 6.90 (dd, *J* = 8.5, 2.1 Hz, 1H), 7.01 (d, *J* = 2.1 Hz, 1H), 8.18 (d, *J* = 7.0 Hz, 1H), 8.20 (d, *J* = 8.5 Hz, 1H), 8.37 (d, *J* = 7.0 Hz, 1H), 9.00 (s, 1H); ^13^C-NMR (125 MHz, methanol-*d*_4_): *δ* = 12.41 (CH_3_), 18.63 (CH_3_), 98.31 (CH), 111.38 (CH), 113.55 (C), 115.93 (C), 116.93 (CH), 119.58 (CH), 121.73 (C), 123.95 (CH), 127.12 (CH), 130.23 (C), 134.44 (C), 146.42 (2C), 157.30 (C), 161.11 (C); MS (EI): *m*/*z* (%) = 262 (100, [M]^+^), 180 (10); MS (ESI, +10 V): *m*/*z* = 263.1 [M + H]^+^, 547 [2M + Na]^+^; HRMS (ESI): calcd. for C_17_H_14_N_2_O: 262.1106, found: 262.1104.

*9-Hydroxyolivacine* (**5**). 9-Methoxyolivacine (**19c**, 38.0 mg, 138 µmol) was dissolved in 48% aqueous HBr (2.3 mL), and the mixture was heated at reflux for 24 h. After cooling to room temperature, the mixture was carefully neutralized using a 25% aqueous solution of ammonia. The mixture was extracted with ethyl acetate until the aqueous layer was colorless. The combined organic layers were washed with water and brine, and then dried (sodium sulfate), and the solvent was evaporated. The residue was purified by column chromatography (silica gel, CH_2_Cl_2_/THF, 4:1 to 2:3) to provide 9-hydroxyolivacine (**5**, 25.2 mg, 96.1 µmol, 70%) as a yellow solid. An additional purification by preparative HPLC provided very pure **5** (6.1 mg, 23 µmol) for biological testing. M.p. 249 °C; UV (MeOH): *λ* = 245, 274, 311, 355, 375 nm; fluorescence (MeOH): *λ*_ex_ = 311, *λ*_em_ = 482 nm; IR (ATR): *ν* = 3220, 2921, 2853, 1734, 1666, 1611, 1425, 1328, 1288, 1185, 1127, 975, 840, 799, 721 cm^–1^; ^1^H-NMR (500 MHz, methanol-*d*_4_): *δ* = 2.96 (s, 3H), 3.36 (s, 3H, HSQC), 7.22 (dd, *J* = 8.6, 2.3 Hz, 1H), 7.51 (d, *J* = 8.6 Hz, 1H), 7.82 (d, *J* = 2.3 Hz, 1H), 8.19 (d, *J* = 7.1 Hz, 1H), 8.38 (d, *J* = 7.1 Hz, 1H), 9.17 (s, 1H); ^13^C-NMR (125 MHz, methanol-*d*_4_): *δ* = 12.42 (CH_3_), 18.67 (CH_3_), 107.97 (CH), 113.13 (CH), 113.96 (C), 119.43 (CH), 119.48 (CH), 119.54 (CH), 121.00 (C), 124.35 (C), 127.40 (CH), 129.67 (C), 134.68 (C), 138.24 (C), 146.56 (C), 153.52 (C), 158.18 (C); MS (EI): *m*/*z* (%) = 262 (100, [M]^+^), 131 (12); MS (ESI, +10 V): *m*/*z* = 263.1 [M + H]^+^; HRMS (ESI): calcd. for C_17_H_14_N_2_O: 262.1106, found: 262.1107.

## 4. Conclusions

In conclusion, we have developed a straightforward synthesis of olivacine (**1**) and four of its oxygenated pyrido[4,3-*b*]carbazole derivatives via Buchwald–Hartwig coupling of the isoquinolinyl triflate **14** and the *ortho*-chloroarylamine **18a-c** followed by a Heck-type cyclization. In a test for the inhibition of the growth of *M. tuberculosis* (strain H_37_Rv), 9-methoxyolivacine (**19c**) proved to be the most active compound, with an MIC_90_ value of 1.5 μM and a relatively low toxicity for a mammalian cell line. These initial results indicate that the pyrido[4,3-*b*]carbazoles are a promising class of compounds for our ongoing search for a carbazole-based tuberculosis drug candidate.

## Figures and Tables

**Table 1:** Optimization of the Heck-type cyclization of **18a** to olivacine (**1**).

	Pd(OAc)_2_ (Equiv.)	Ligand ^1^ (Equiv.)	K_2_CO_3_ (Equiv.)	Solvent	Temp. (°C)	Time (h)	Yield (%)	RSM ^2^ (%)
1	0.1	0.2	2	DMA	110	24	11	60
2	0.1	0.2	2	DMA	130	1.5	46	18
3	0.2	0.4	4	DMA	120	3.0	35	35
4 ^3^	0.5	1.0	10	DMF	120	45	46	31
5	0.2	0.4	4	DMF	140	3.0	62	7
6	0.3	0.6	4	DMF	140	0.5	71	–

^1^ P(*t*Bu)_3_·HBF_4_ was used as ligand; ^2^ RSM = reisolated starting material; ^3^ reagents added in portions of 0.1 equiv. Pd(OAc)_2_, 0.2 equiv. ligand, 2 equiv. K_2_CO_3_ after 0, 1, 3, 6, 30 h of reaction time.

**Table 2:** Inhibitory activity against *M. tuberculosis* of olivacine (**1**) and its oxygenated derivatives **4**, **5**, **19b**, and **19c**.

Compound	MIC_90_ (µM) ^1^	IC_50_ (µM) ^2^	SI ^3^
Olivacine (**1**)	4.7	18.05	3.8
8-Hydroxyolivacine (**4**)	n.d.^4^	n.d.	–
9-Hydroxyolivacine (**5**)	n.d.^4^	n.d.	–
8-Methoxyolivacine (**19b**)	n.d.^4^	n.d.	–
9-Methoxyolivacine (**19c**)	1.5	24.5	16.3
3-Methoxy-2-methyl-carbazole-1,4-quinone ^5^	4.0	>50	>12.5
Isoniazid ^5^	0.24	>50	>208
Rifampicin ^5^	0.02	>50	>2500

^1^ Minimum inhibitory concentrations (µM) against *M. tuberculosis* H_37_Rv in the microplate Alamar blue assay (MABA); values are the mean of three replicate experiments; n.d. = not determined. ^2^ Cytotoxicity corresponding to the concentration (µM) effecting a 50% decrease in tetrazolium dye reduction by Vero cells (African green monkey kidney cells); values are the mean of three replicate experiments; for experiments giving a value higher than the max. concentration used, >50 µM is denoted. ^3^ Selectivity index: SI = IC_50_/MIC_90_. ^4^ These compounds showed no significant inhibition in a preliminary assay. ^5^ 3-Methoxy-2-methylcarbazole-1,4-quinone, isoniazid, and rifampicin (rifampin) were used as positive control; solvent was used as negative control.

## Supplementary Materials

The following data are available online. Copies of the ^1^H-NMR, ^13^C-NMR and 2D NMR spectra.
