# Supplementary material for: Synthesis and Activity against Mycobacterium tuberculosis of Olivacine and Oxygenated Derivatives
Source: Molecules. 2018 Jun 9;23(6):1402. doi: 10.3390/molecules23061402 (PMC6100493; doi:10.3390/molecules23061402)
Supplement: Supplementary file 1 [file molecules-23-01402-s001.zip › molecules-304783-SI.pdf]

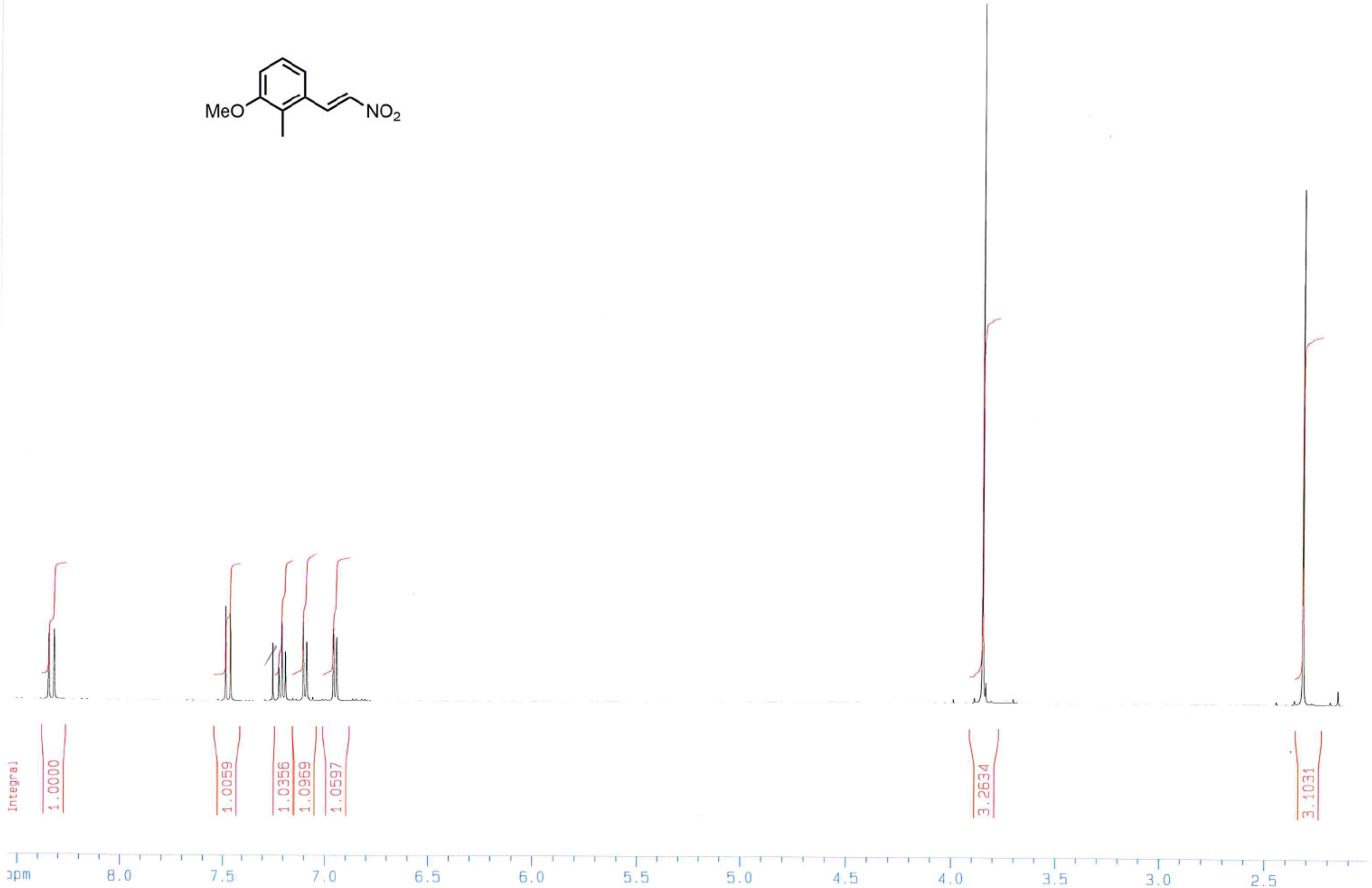
MeO
NO2
Integral
1.0000
1.0059
1.0356
1.0969
1.0597
3.2634
3.1031
ppm
8.0
7.5
7.0
6.5
6.0
5.5
5.0
4.5
4.0
3.5
3.0
2.5

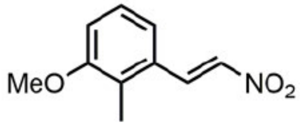
MeO
NO2

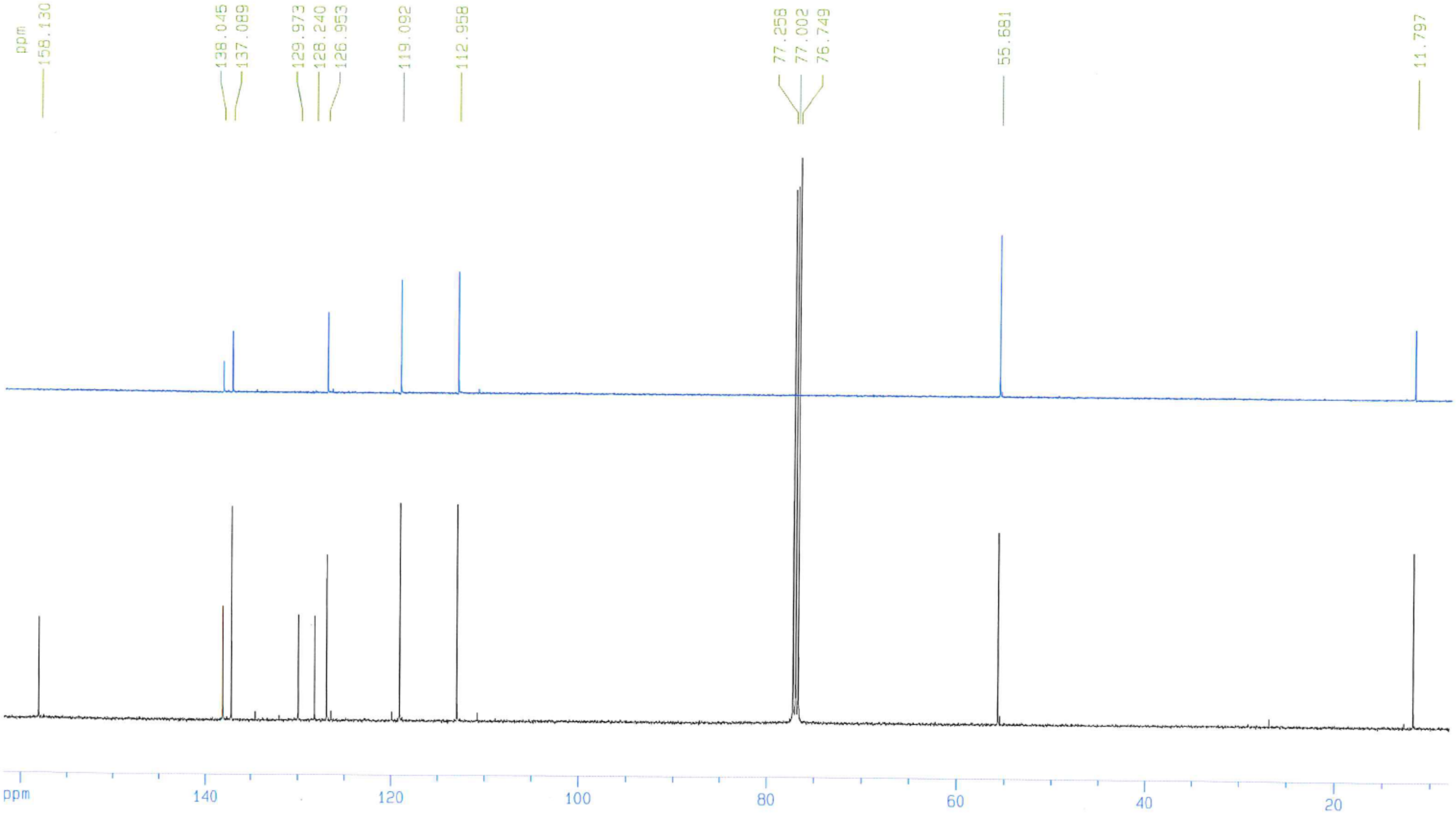
ppm
158.130
138.045
137.089
129.973
128.240
126.953
119.092
112.958
77.258
77.002
76.749
55.681
11.797
ppm
140
120
100
80
60
40
20

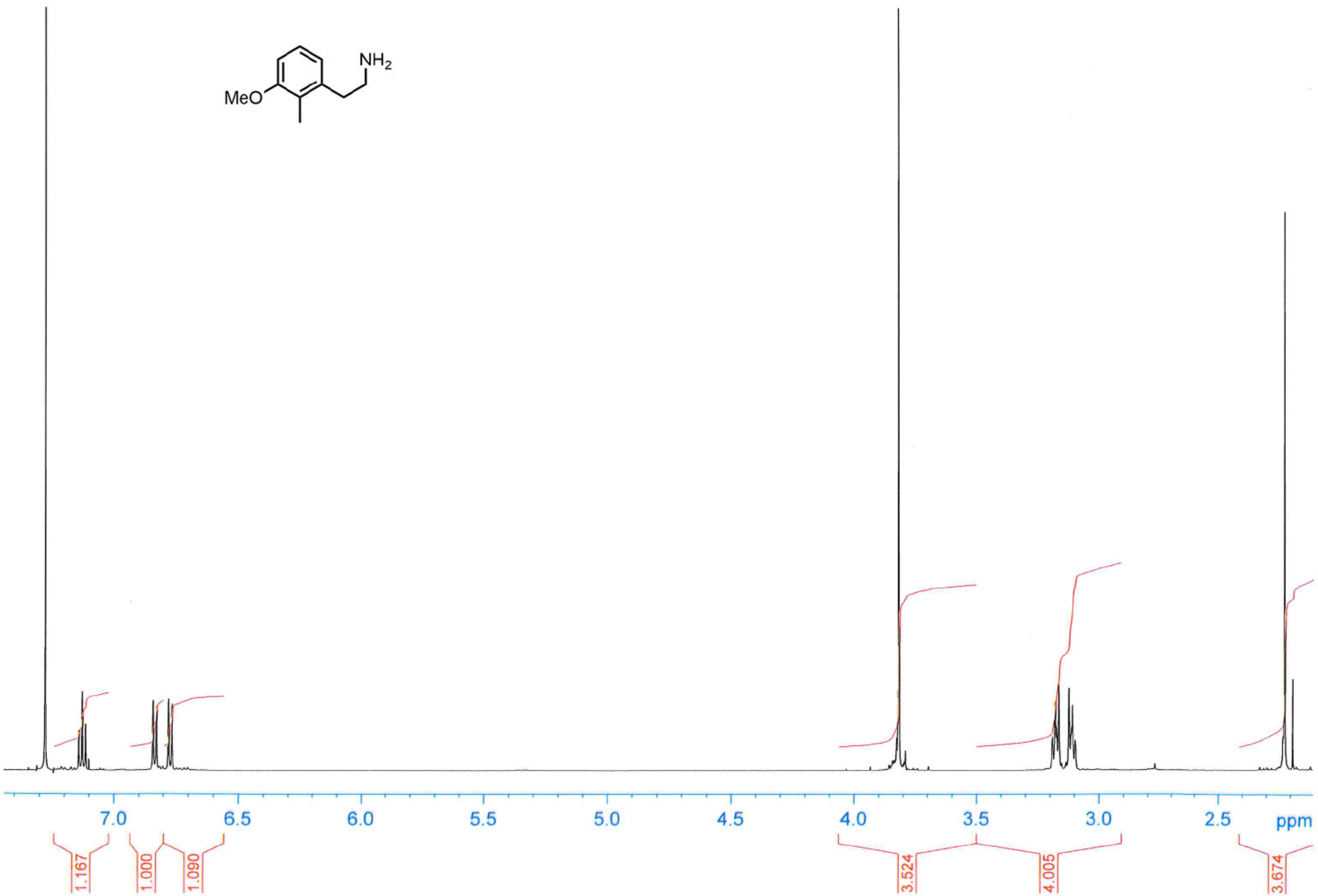
NH2
MeO
7.0
6.5
6.0
5.5
5.0
4.5
4.0
3.5
3.0
2.5
ppm
1.167
1.000
1.090
3.524
4.005
3.674

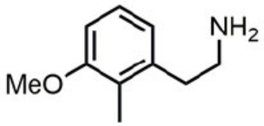
NH2
MeO

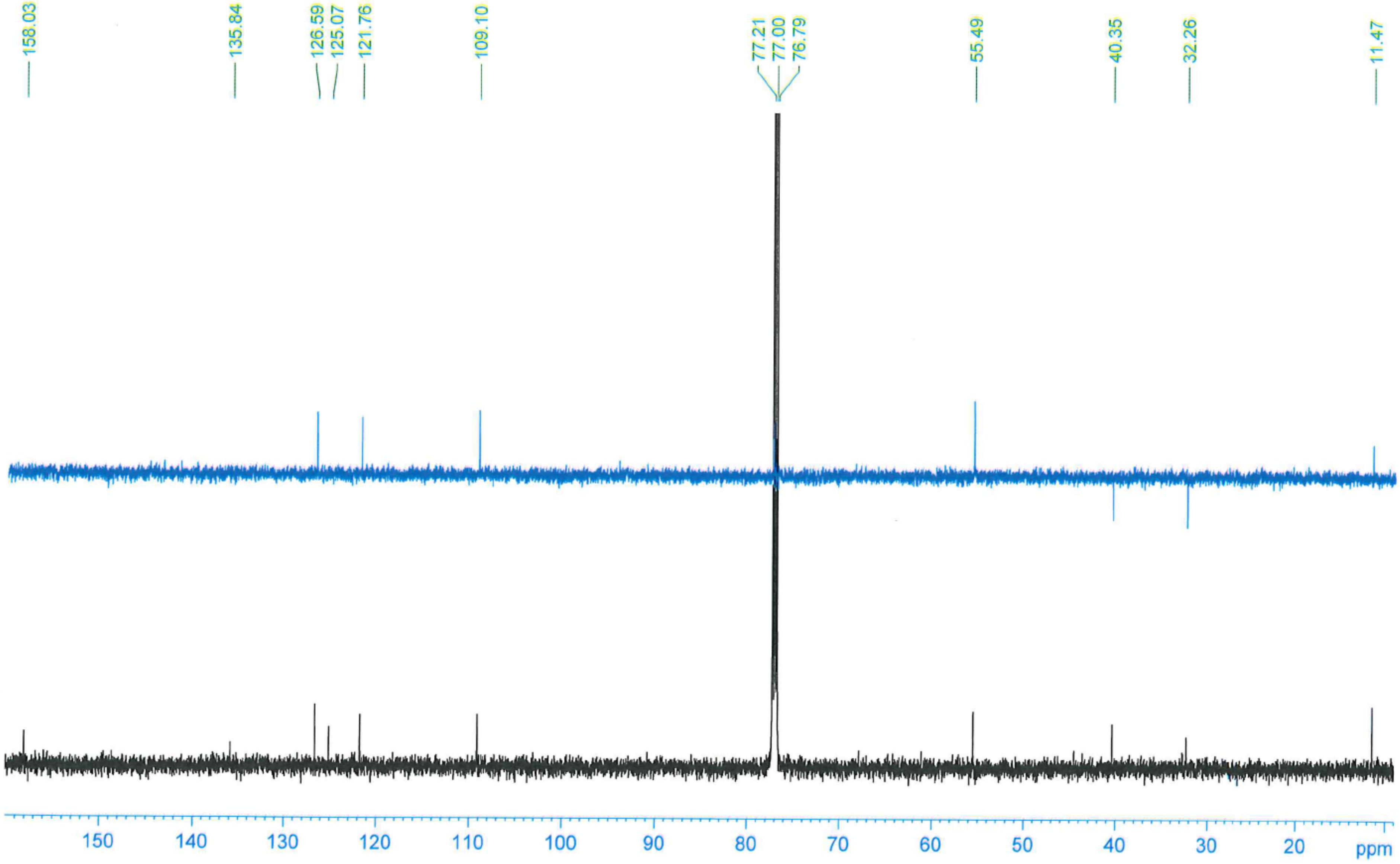
158.03
135.84
126.59
125.07
121.76
109.10
77.21
77.00
76.79
55.49
40.35
32.26
11.47
150
140
130
120
110
100
90
80
70
60
50
40
30
20
ppm

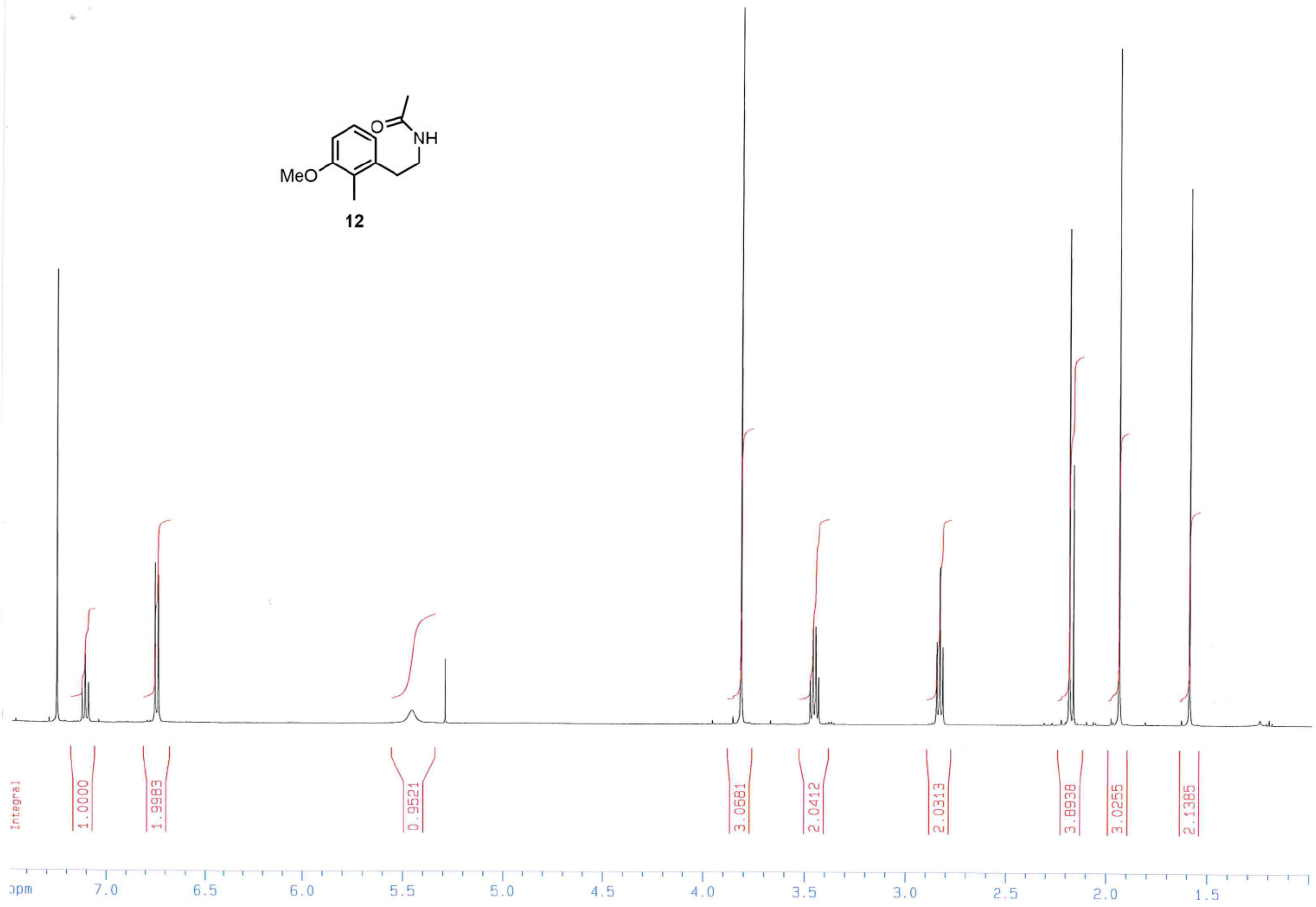

MeO
O
NH
12
Integral
1.0000
1.9983
0.9521
3.0681
2.0412
2.0313
3.8938
3.0255
2.1385
ppm
7.0
6.5
6.0
5.5
5.0
4.5
4.0
3.5
3.0
2.5
2.0
1.5

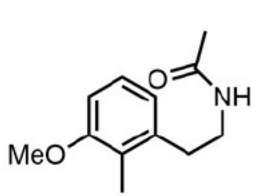
MeO
O
NH

12

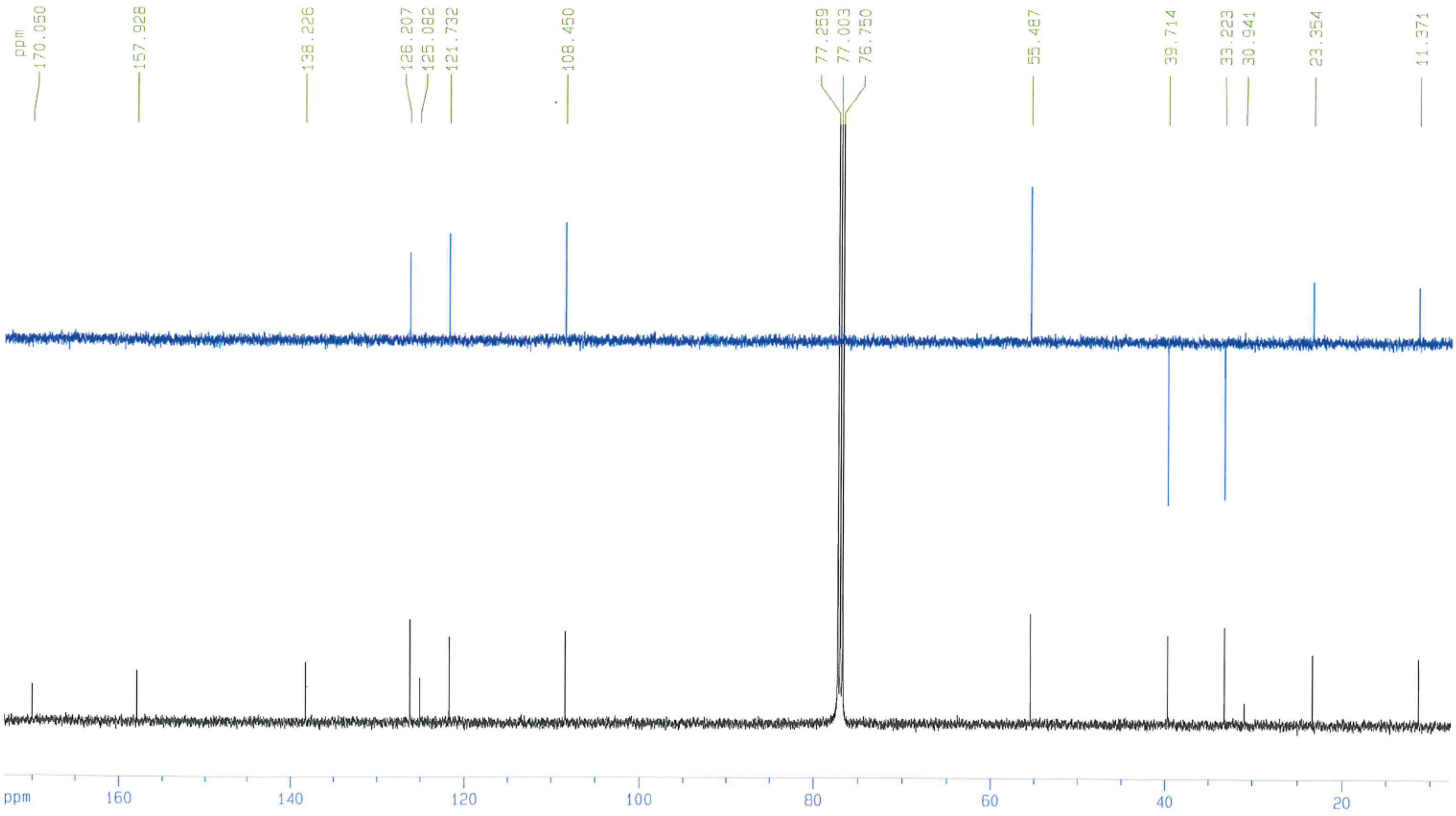
ppm
170.050
157.928
138.226
126.207
125.082
121.732
108.450
77.259
77.003
76.750
55.487
39.714
33.223
30.941
23.354
11.371
ppm
160
140
120
100
80
60
40
20

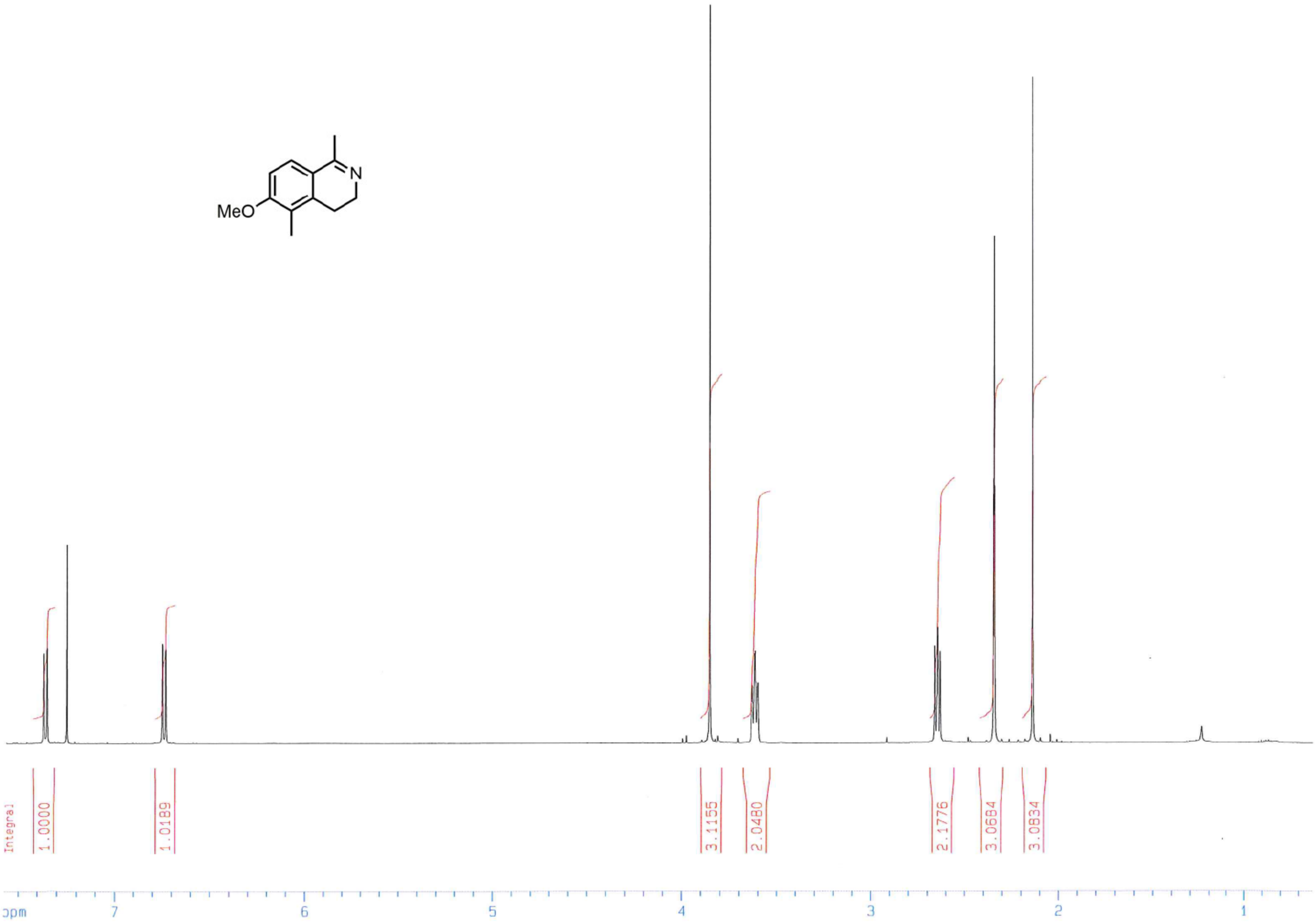
MeO
N
Integral
1.0000
1.0189
3.1155
2.0480
2.1776
3.0684
3.0834
ppm
7
6
5
4
3
2
1

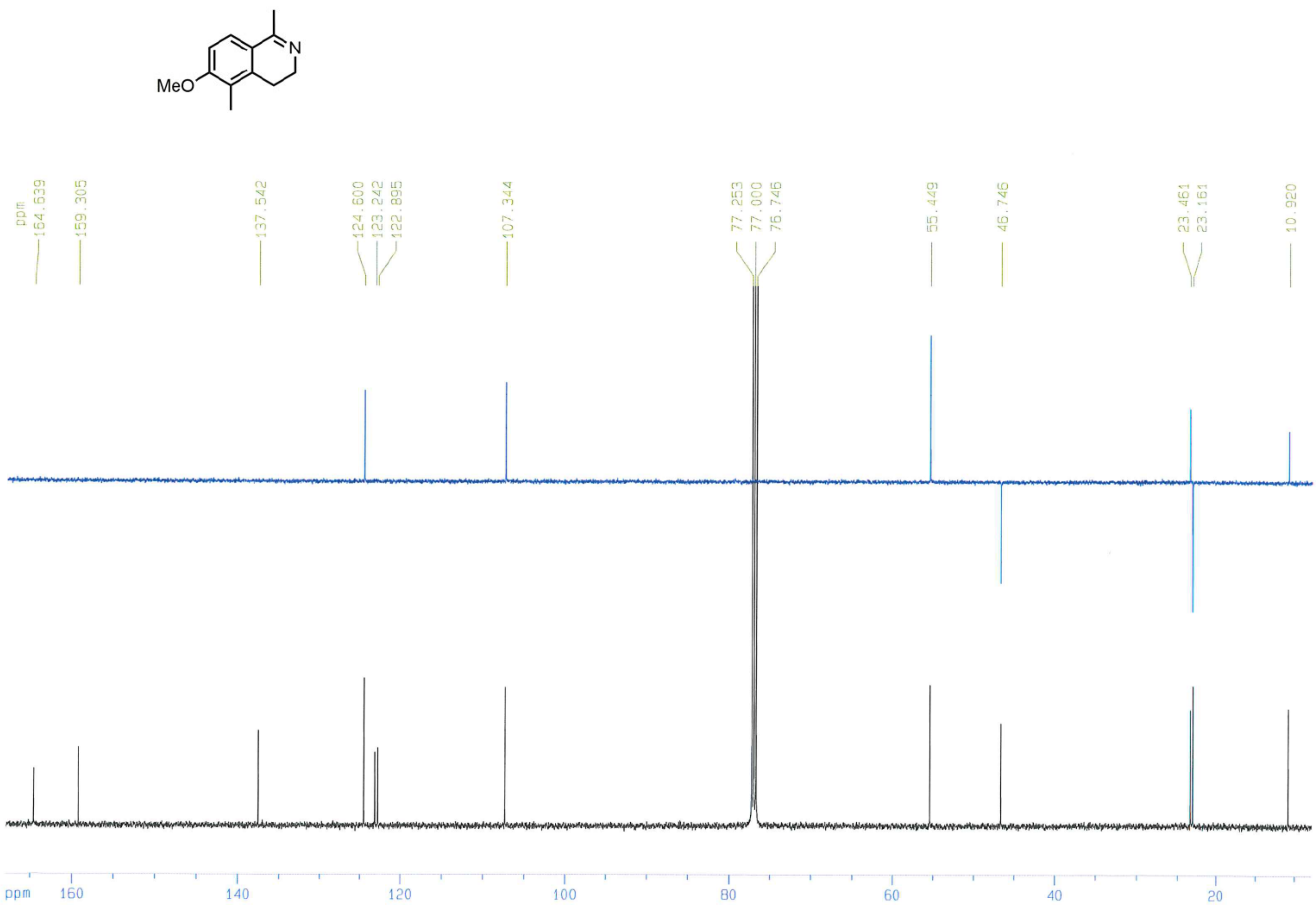

MeO
N
ppm
164.639
159.305
137.542
124.600
123.242
122.895
107.344
77.253
77.000
76.746
55.449
46.746
23.461
23.161
10.920
ppm 160 140 120 100 80 60 40 20

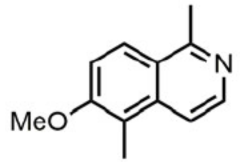

MeO
N

13

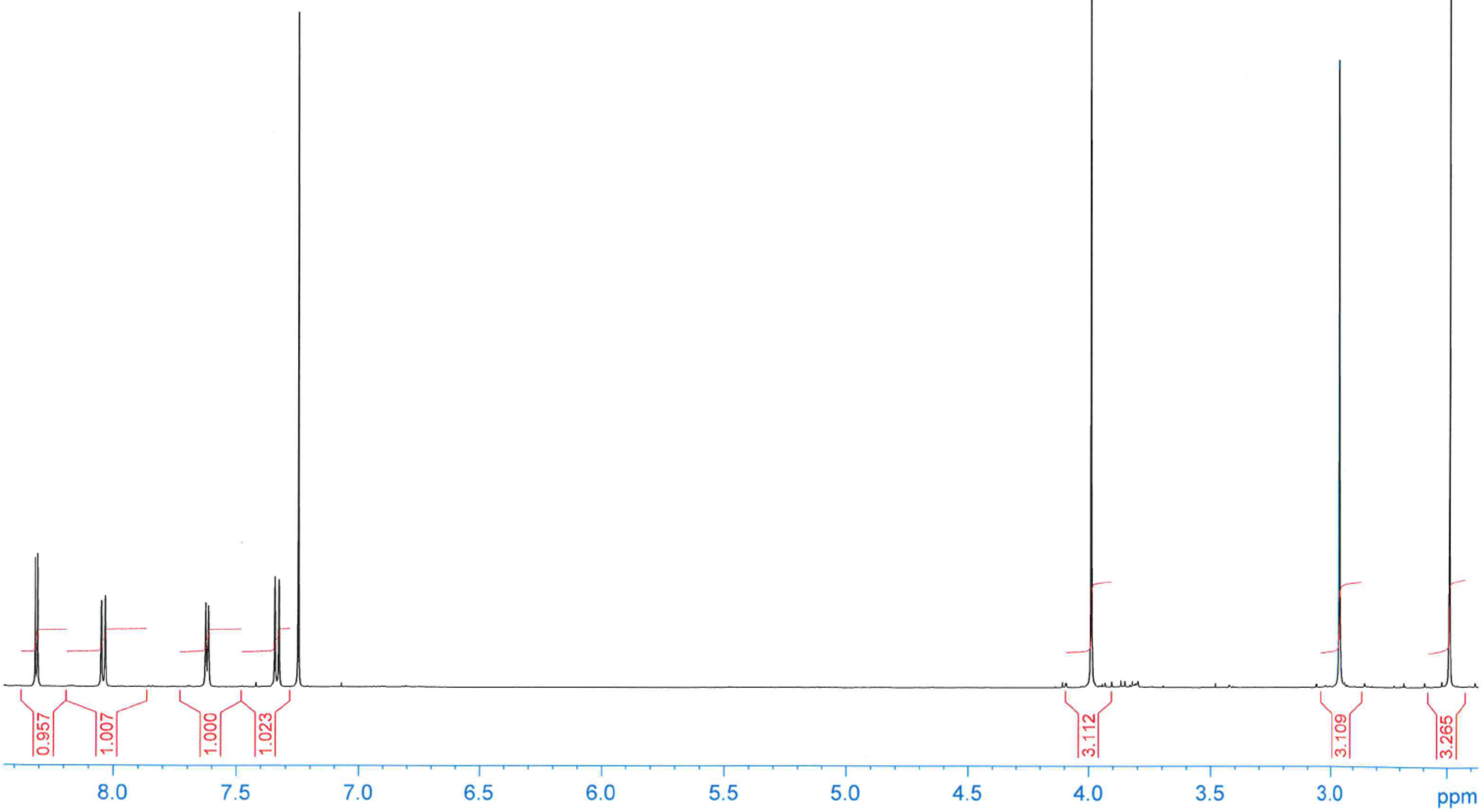

0.957
1.007
1.000
1.023
3.112
3.109
3.265
8.0
7.5
7.0
6.5
6.0
5.5
5.0
4.5
4.0
3.5
3.0
ppm

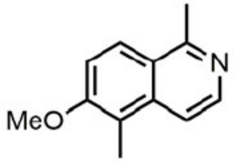

13

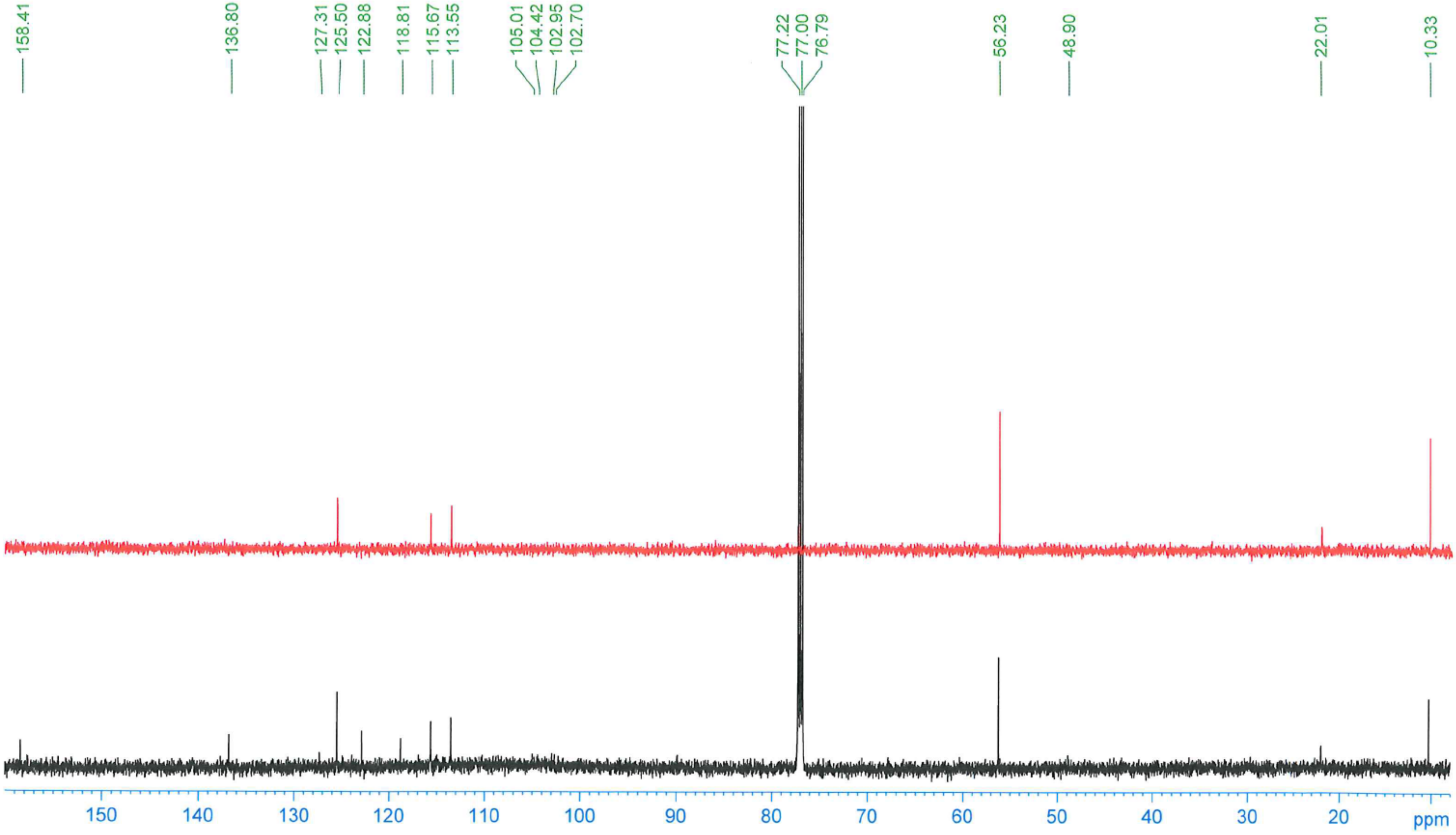

158.41
136.80
127.31
125.50
122.88
118.81
115.67
113.55
105.01
104.42
102.95
102.70
77.22
77.00
76.79
56.23
48.90
22.01
10.33
150
140
130
120
110
100
90
80
70
60
50
40
30
20
ppm

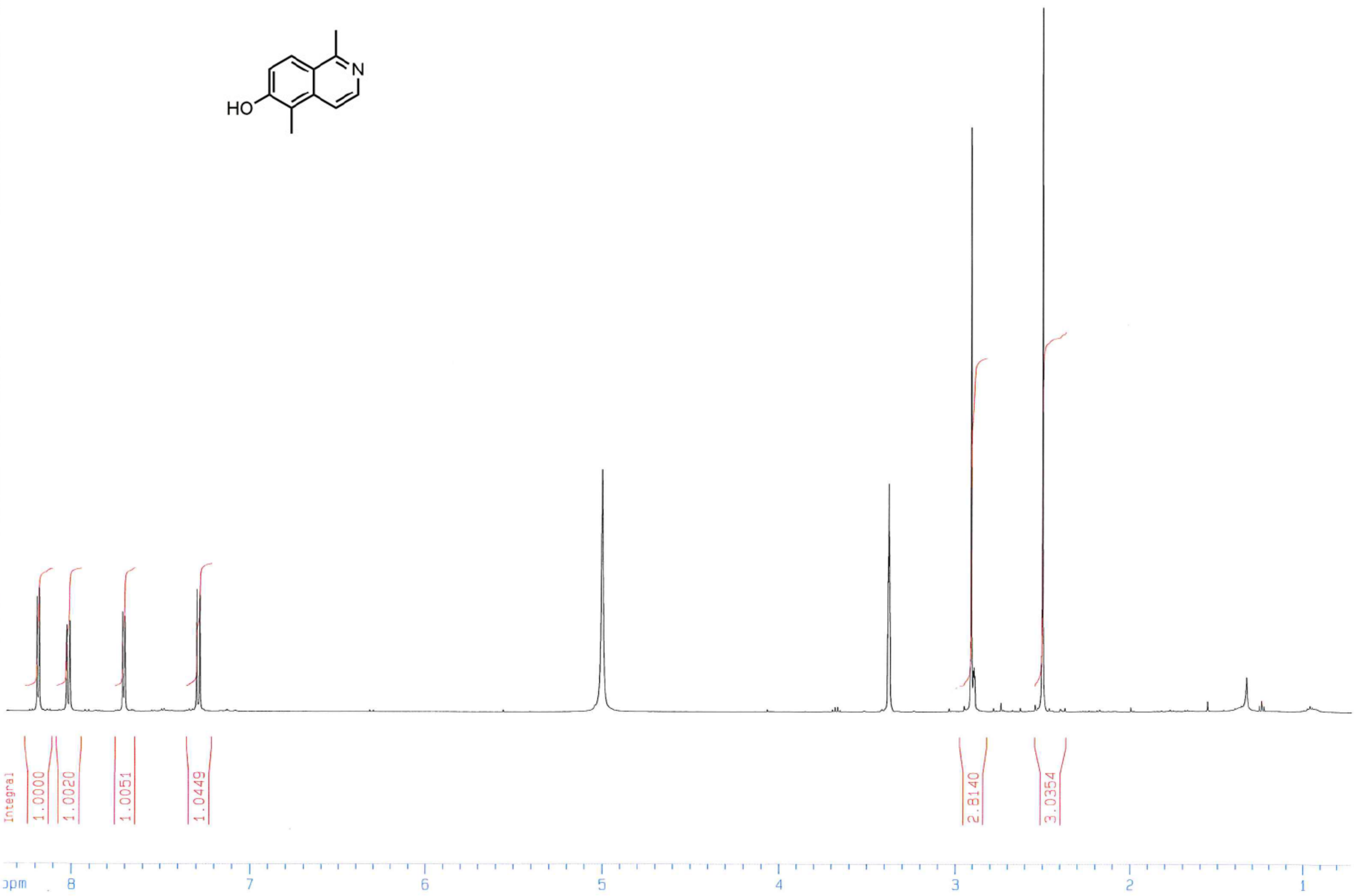

Integral
1.0000
1.0020
1.0051
1.0449
2.8140
3.0354
ppm
8
7
6
5
4
3
2
1

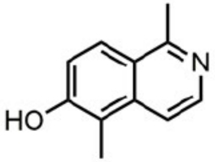
HO
N

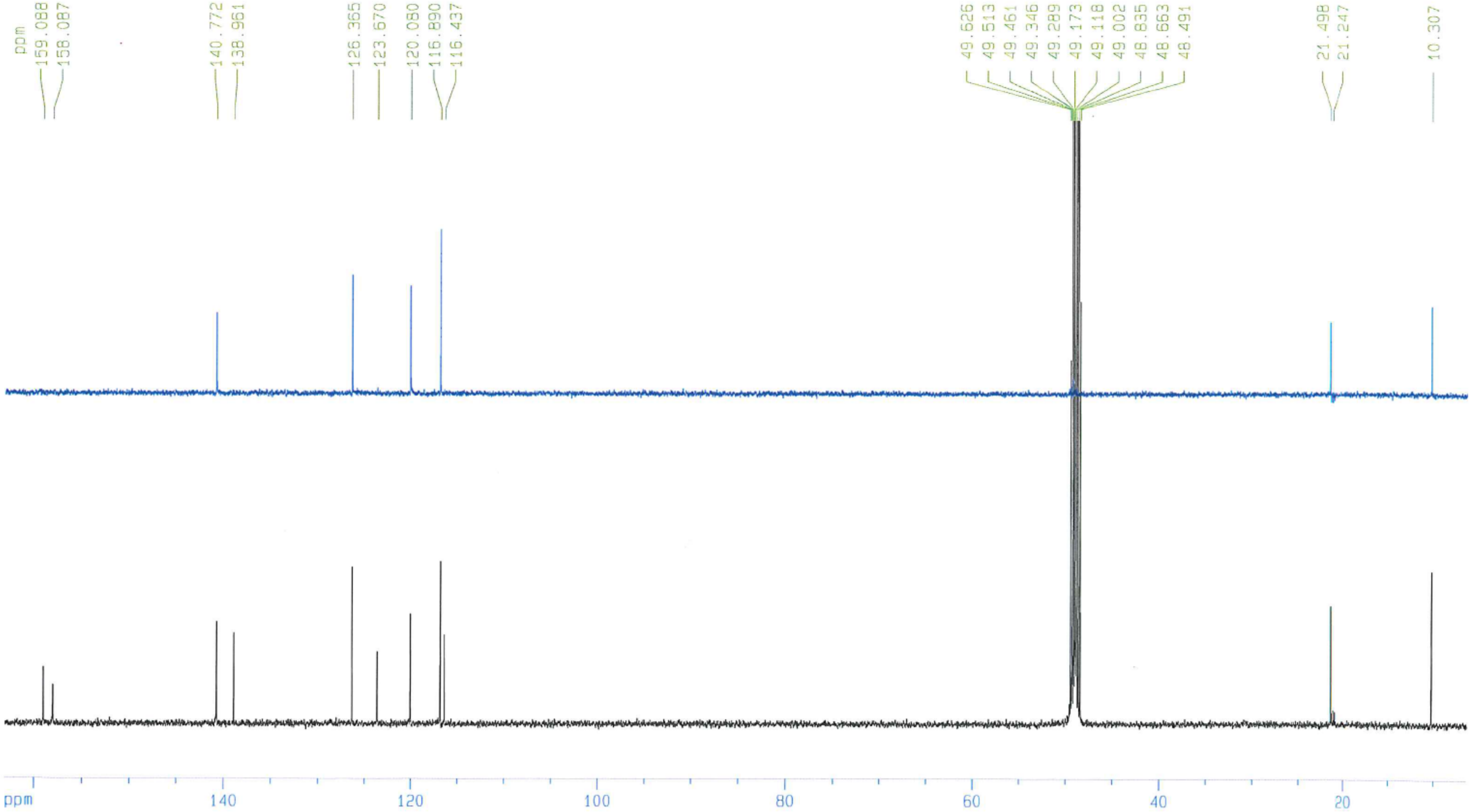
ppm
159.088
158.087
140.772
138.961
126.365
123.670
120.080
116.890
116.437
49.626
49.513
49.461
49.346
49.289
49.173
49.118
49.002
48.835
48.663
48.491
21.498
21.247
10.307
ppm
140
120
100
80
60
40
20

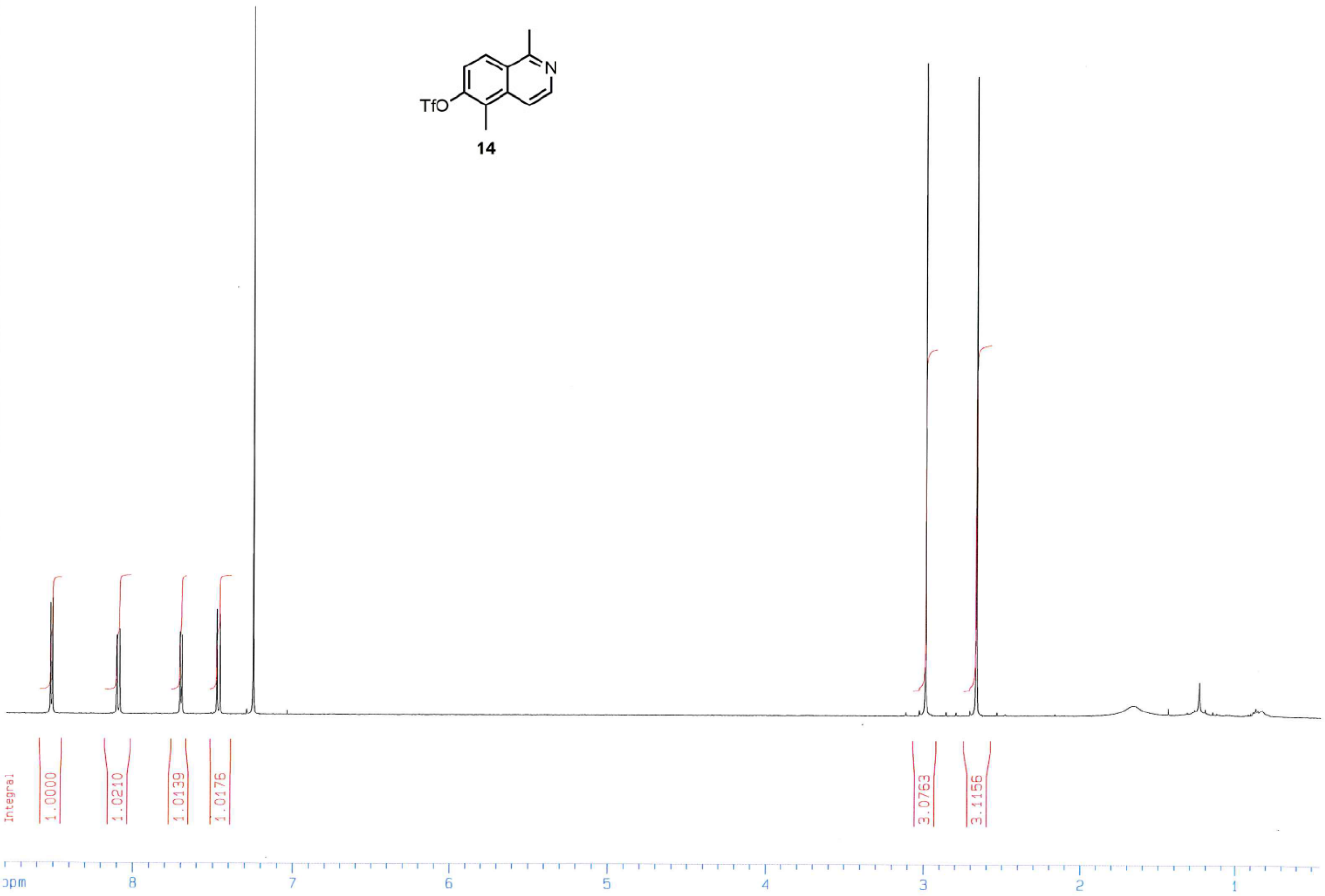

TfO
N
14
Integral
1.0000
1.0210
1.0139
1.0176
3.0763
3.1156
ppm
8
7
6
5
4
3
2
1

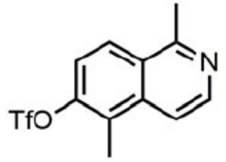
TfO
N

14

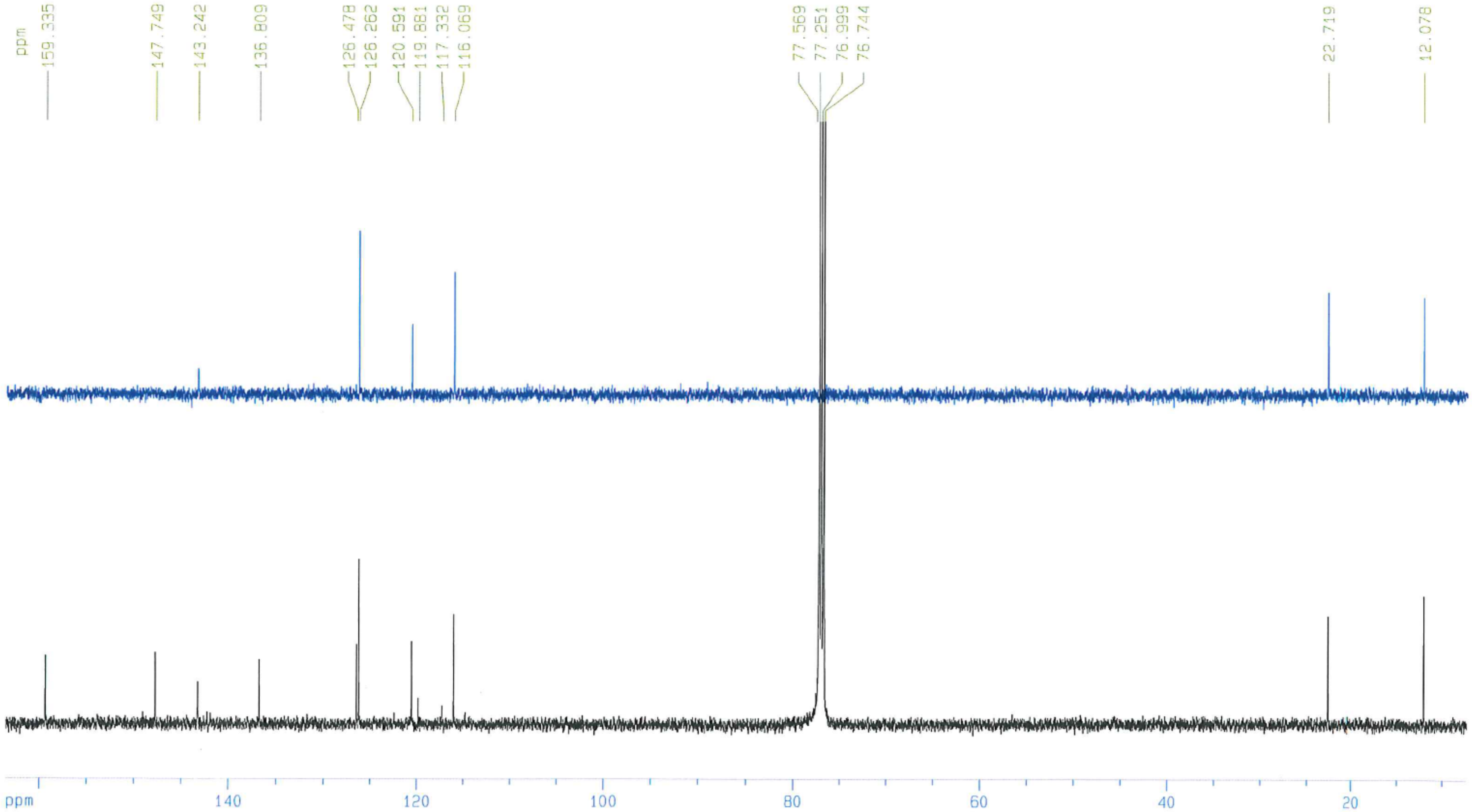
ppm
159.335
147.749
143.242
136.809
126.478
126.262
120.591
119.881
117.332
116.069
77.569
77.251
76.999
76.744
22.719
12.078
ppm
140
120
100
80
60
40
20

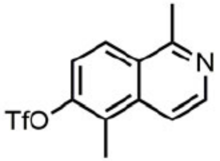

14

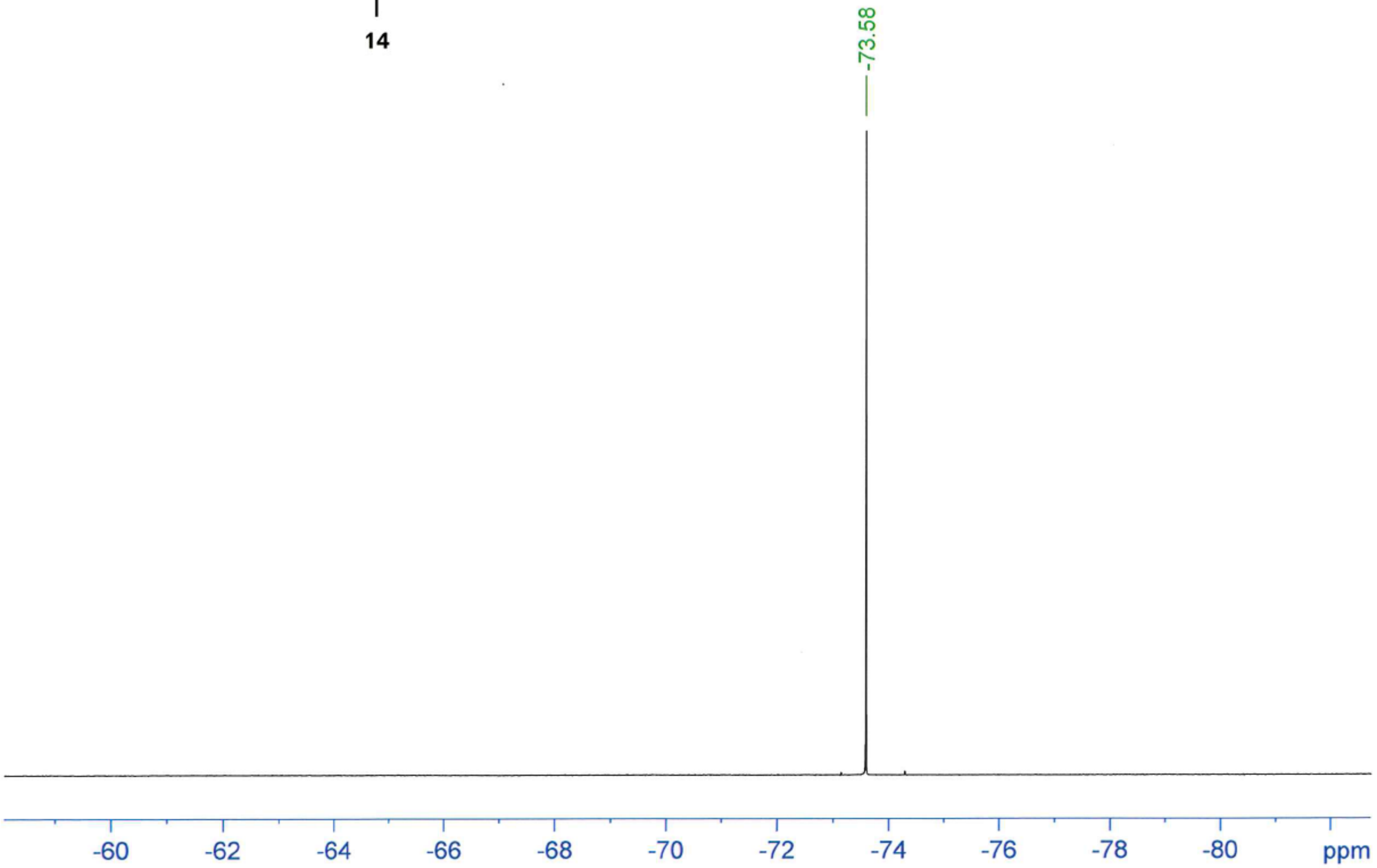

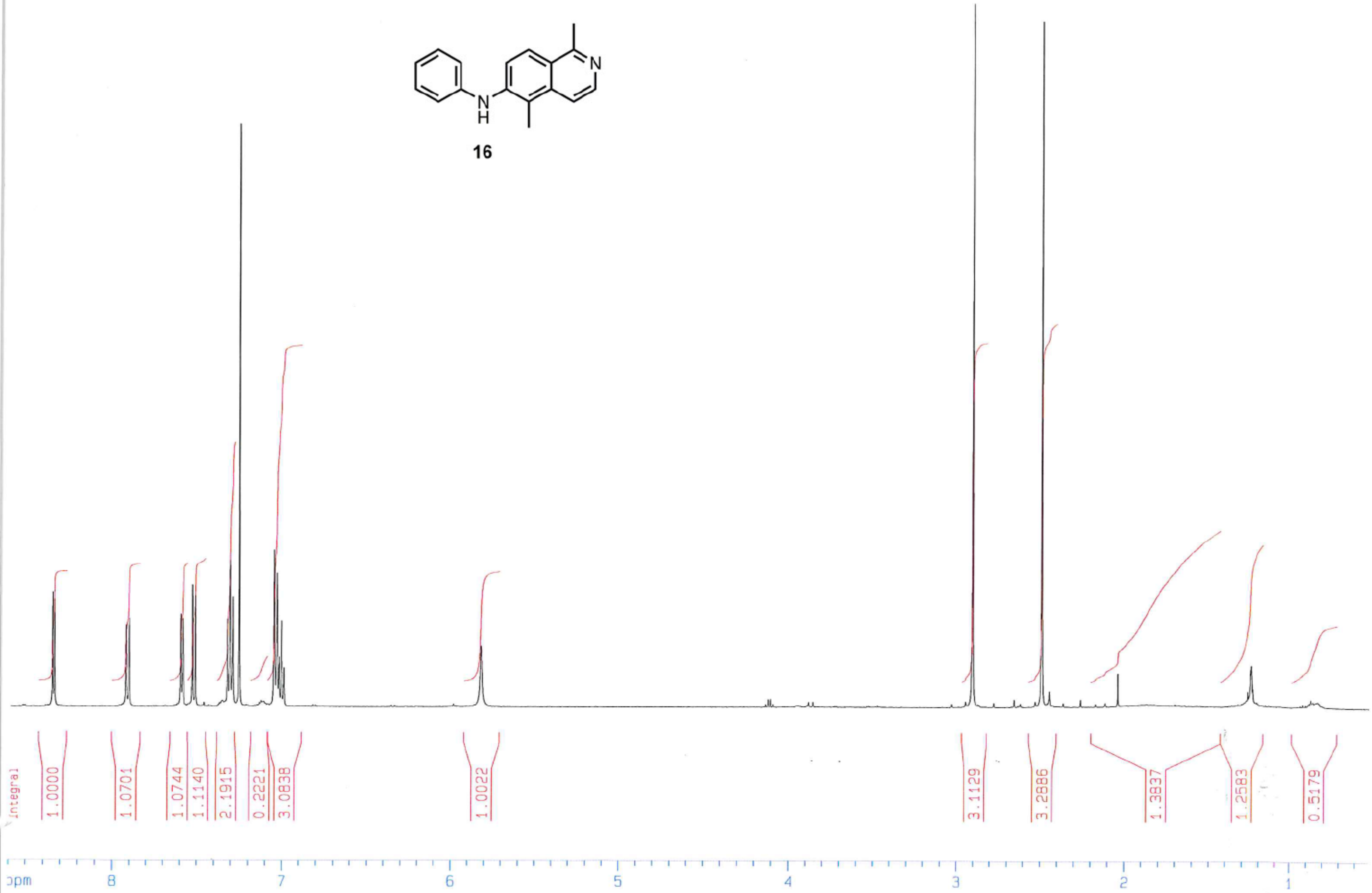
N
H
N
16
Integral
1.0000
1.0701
1.0744
1.1140
2.1915
0.2221
3.0838
1.0022
3.1129
3.2886
1.3837
1.2583
0.5179
ppm
8
7
6
5
4
3
2
1

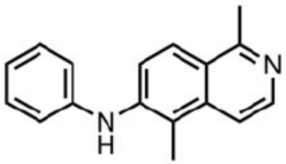
N
H
N

16

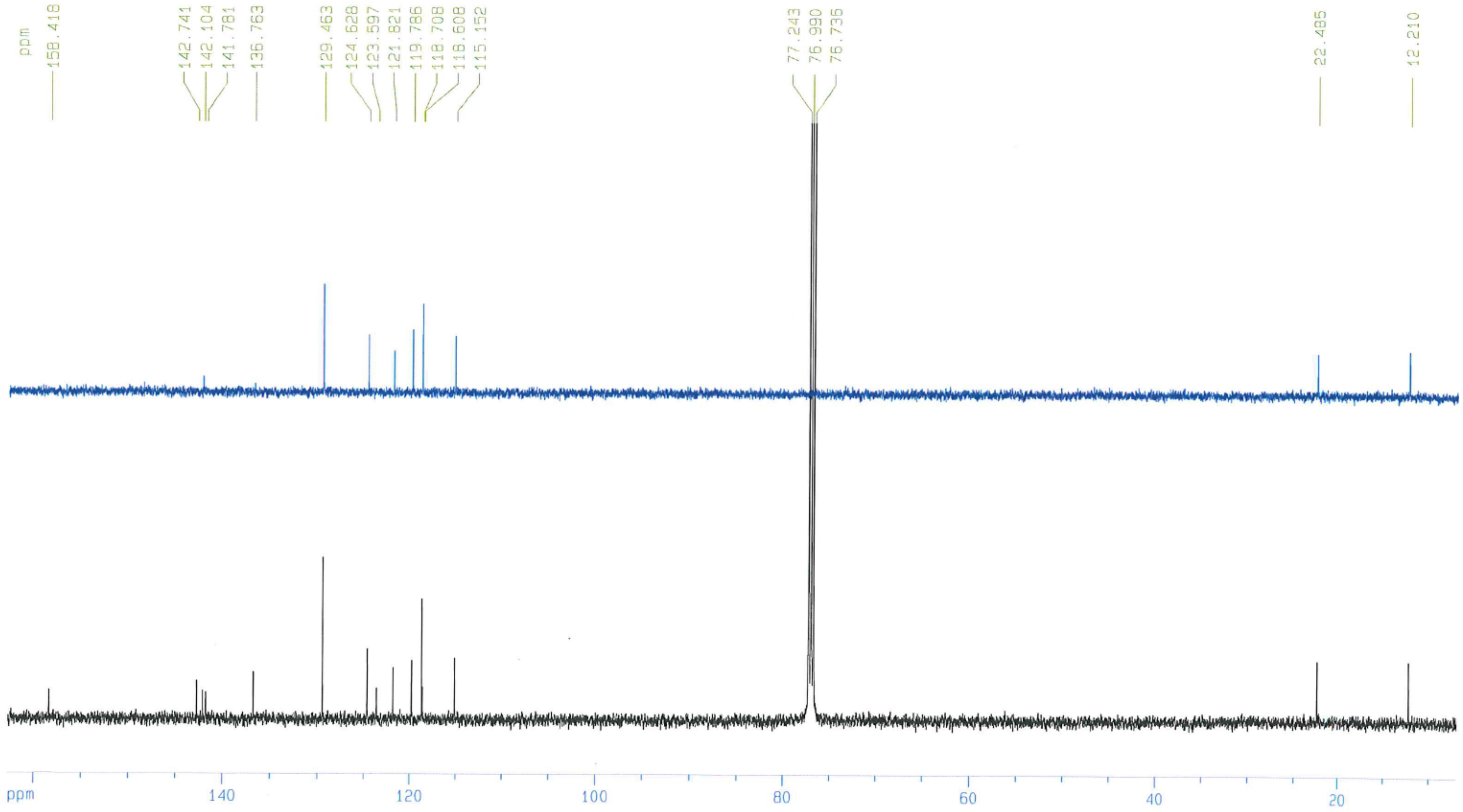
ppm
158.418
142.741
142.104
141.781
136.763
129.463
124.628
123.597
121.821
119.786
118.708
118.608
115.152
77.243
76.990
76.735
22.485
12.210
ppm
140
120
100
80
60
40
20

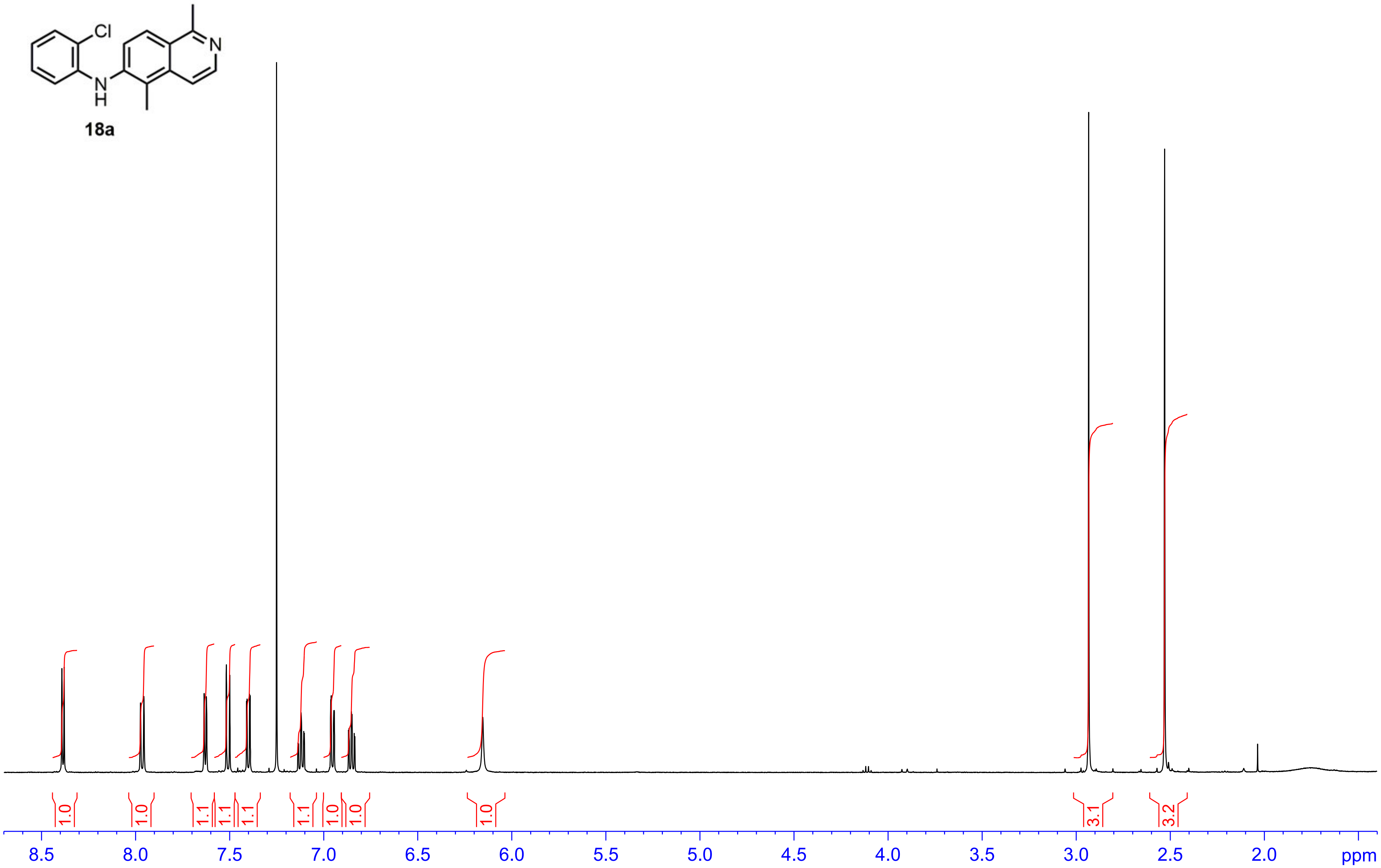

Cl
N
H
N
1.0
1.0
1.1
1.1
1.1
1.1
1.0
1.0
1.0
3.1
3.2
8.5
8.0
7.5
7.0
6.5
6.0
5.5
5.0
4.5
4.0
3.5
3.0
2.5
2.0
ppm

18a

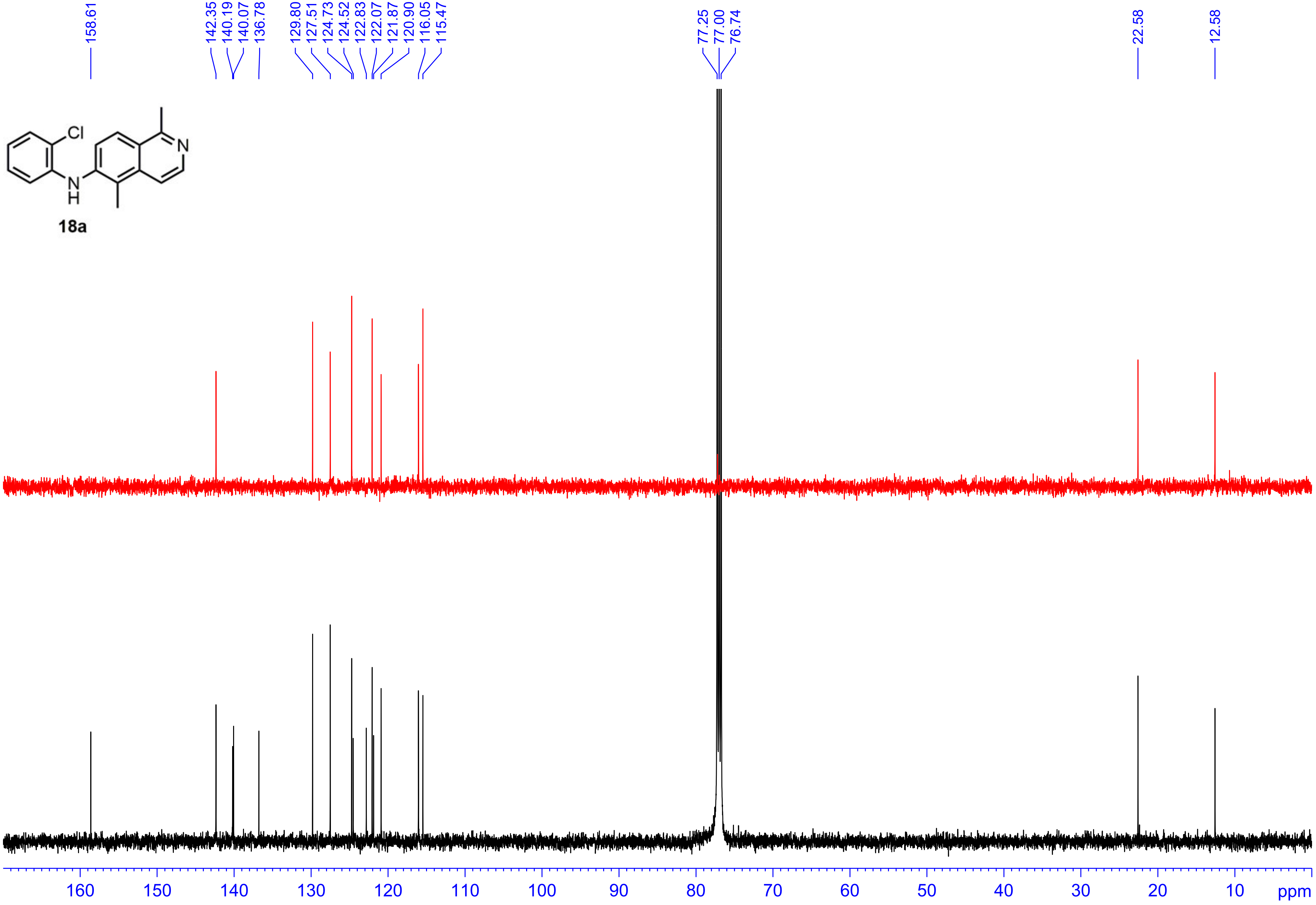
158.61
142.35
140.19
140.07
136.78
129.80
127.51
124.73
124.52
122.83
122.07
121.87
120.90
116.05
115.47
77.25
77.00
76.74
22.58
12.58
Cl
N
N
H
18a
160 150 140 130 120 110 100 90 80 70 60 50 40 30 20 10 ppm

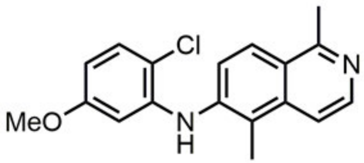
Cl
N
MeO
N
H

18b

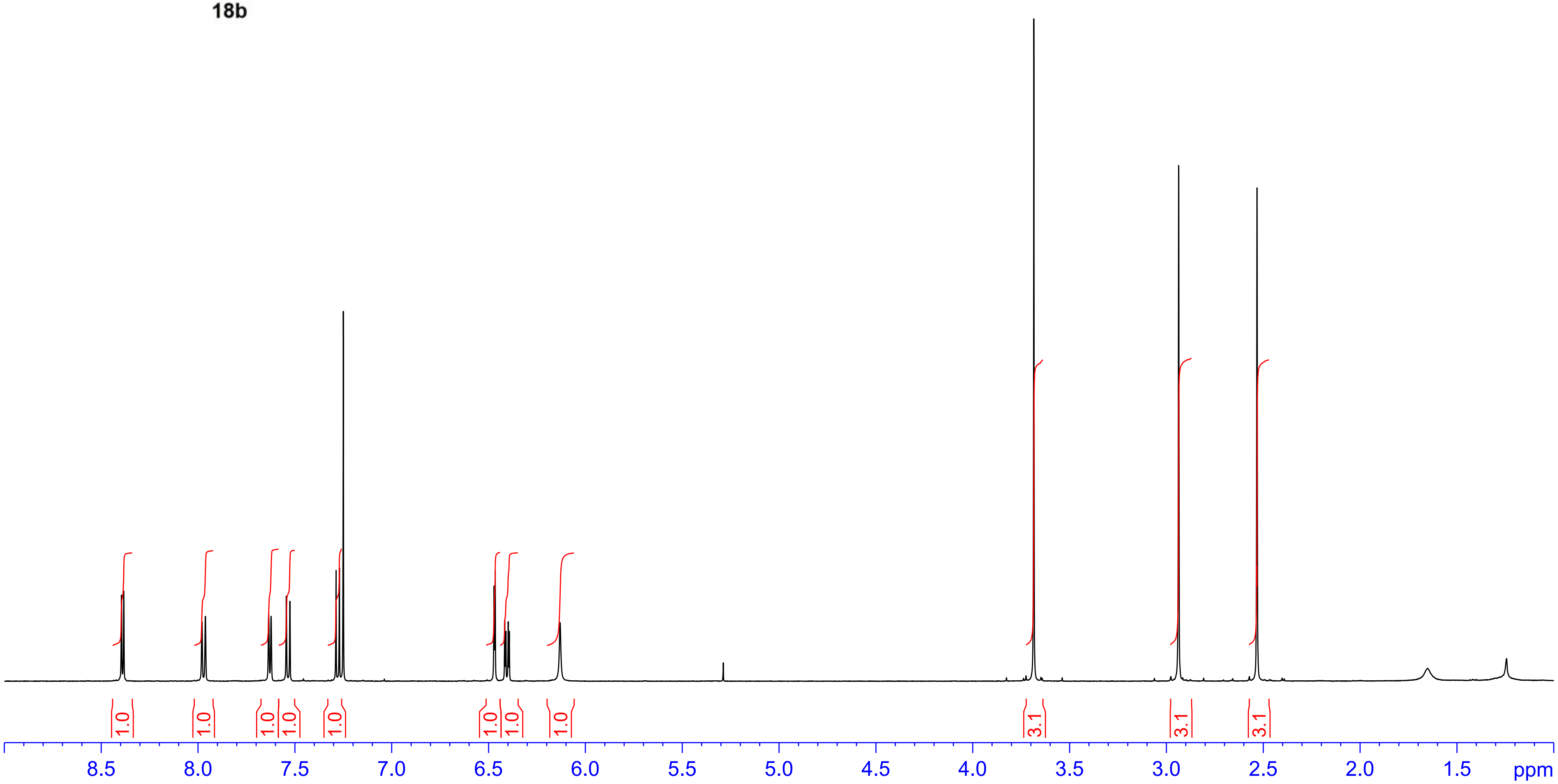
1.0
1.0
1.0
1.0
1.0
1.0
1.0
1.0
3.1
3.1
3.1
8.5
8.0
7.5
7.0
6.5
6.0
5.5
5.0
4.5
4.0
3.5
3.0
2.5
2.0
1.5
ppm

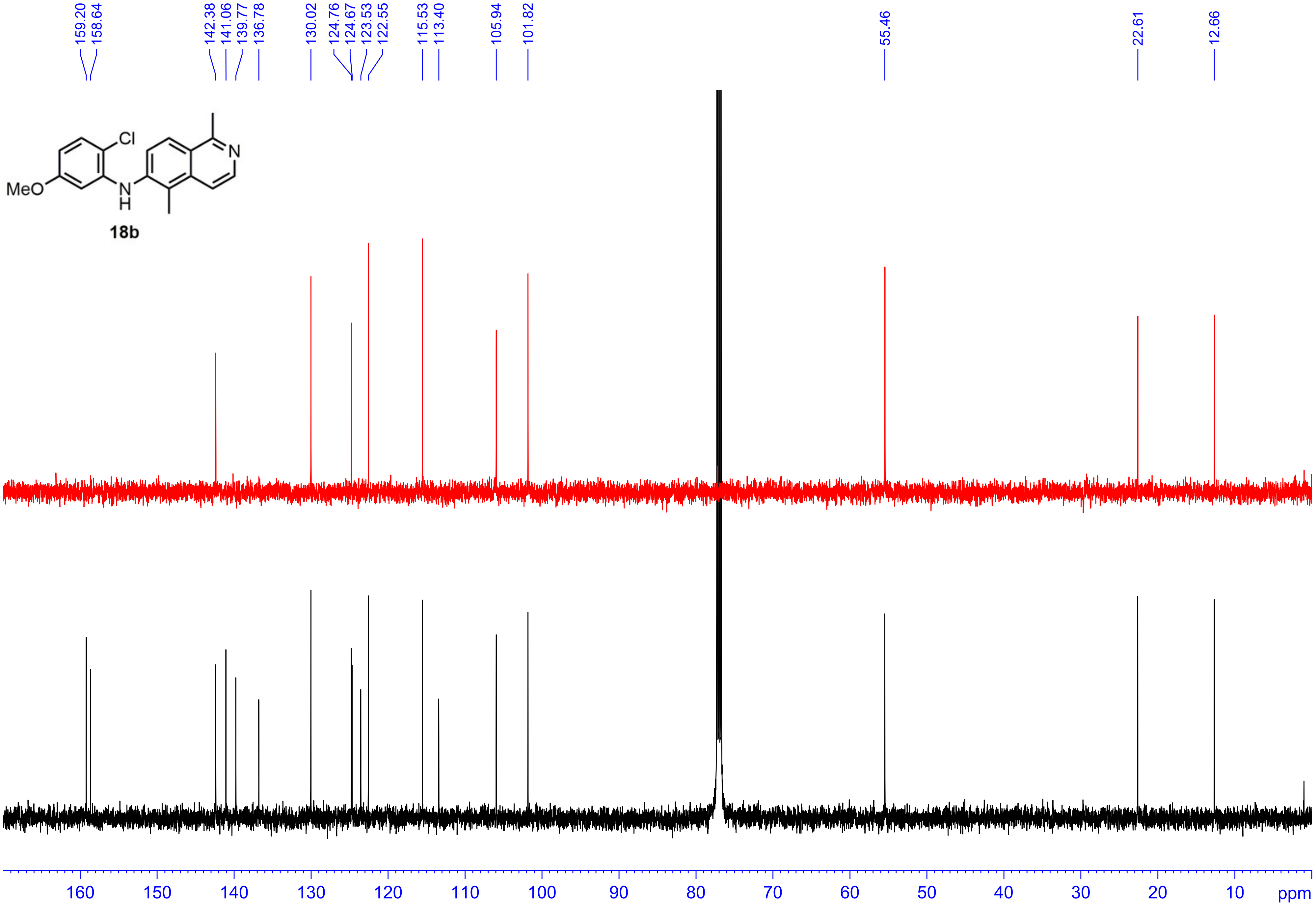
159.20
158.64
142.38
141.06
139.77
136.78
130.02
124.76
124.67
123.53
122.55
115.53
113.40
105.94
101.82
55.46
22.61
12.66
Cl
N
MeO
N
H
18b
160
150
140
130
120
110
100
90
80
70
60
50
40
30
20
10
ppm

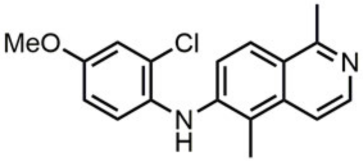
MeO
Cl
N
N
H

18c

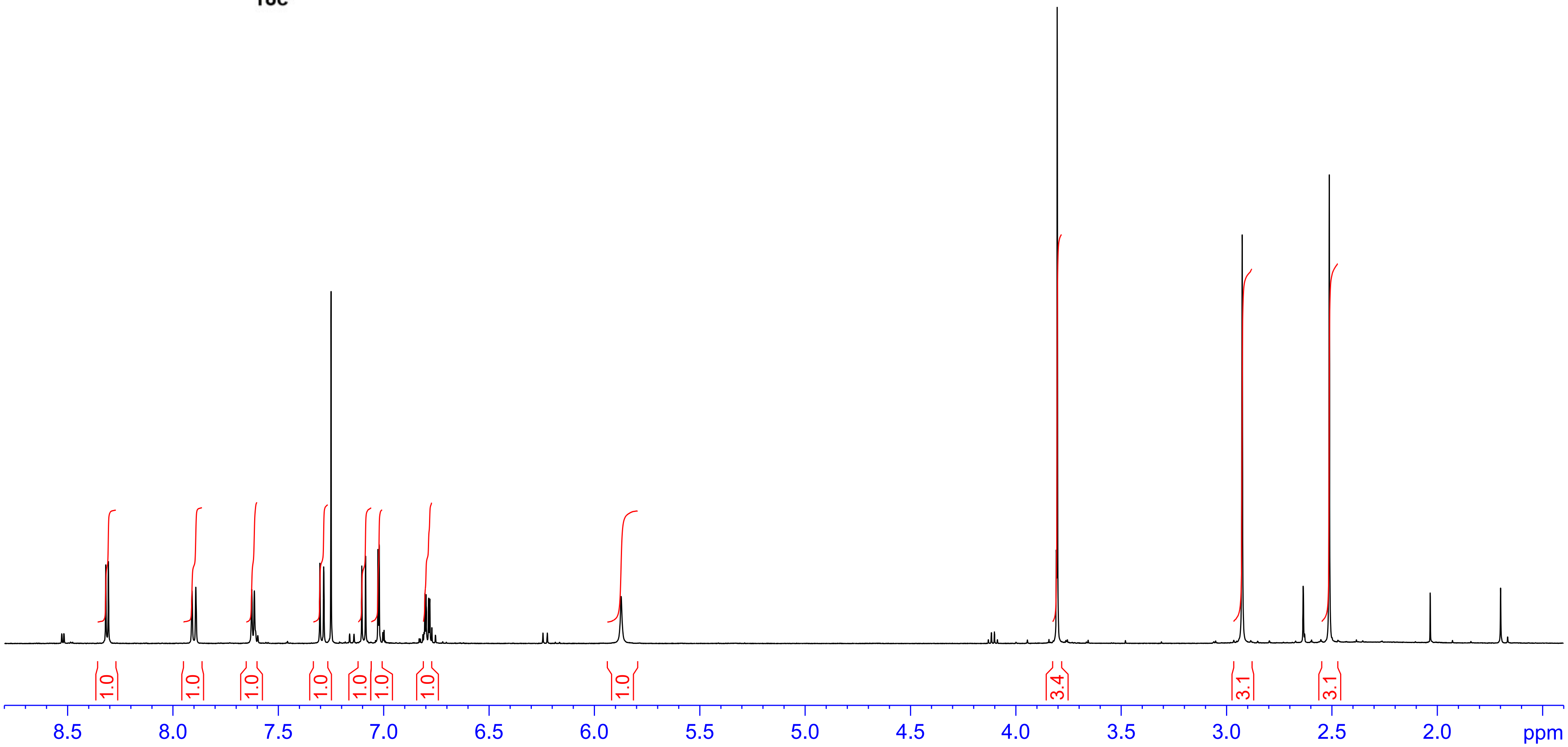
1.0
1.0
1.0
1.0
1.0
1.0
1.0
1.0
3.4
3.1
3.1
8.5
8.0
7.5
7.0
6.5
6.0
5.5
5.0
4.5
4.0
3.5
3.0
2.5
2.0
ppm

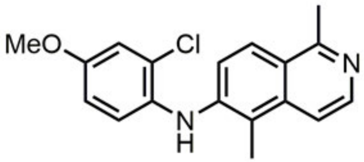
MeO
Cl
N
H
N

18c

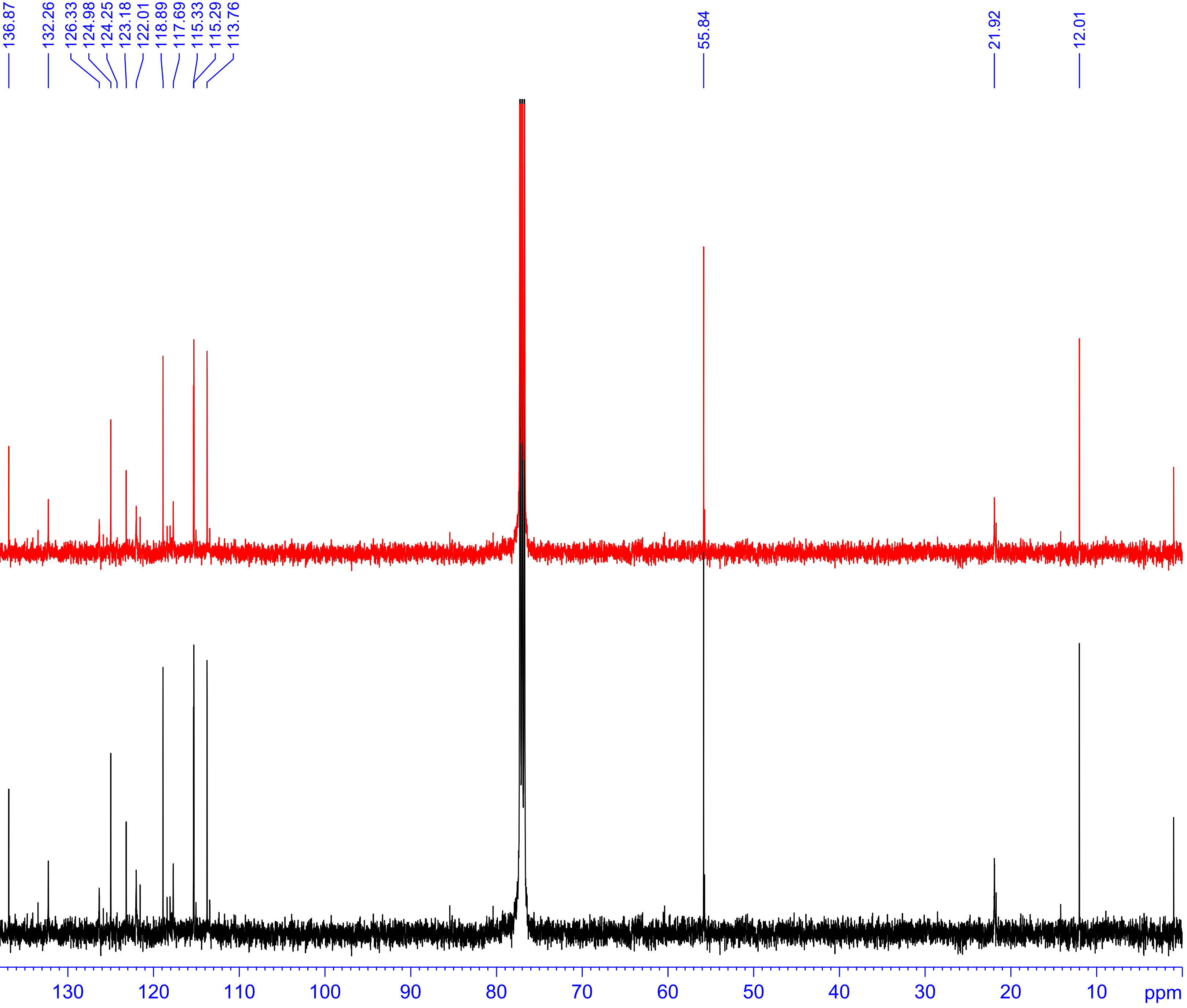
158.06
155.61
142.90
140.79
136.87
132.26
126.33
124.98
124.25
123.18
122.01
118.89
117.69
115.33
115.29
113.76
55.84
21.92
12.01
160
150
140
130
120
110
100
90
80
70
60
50
40
30
20
10
ppm

18c HSQC

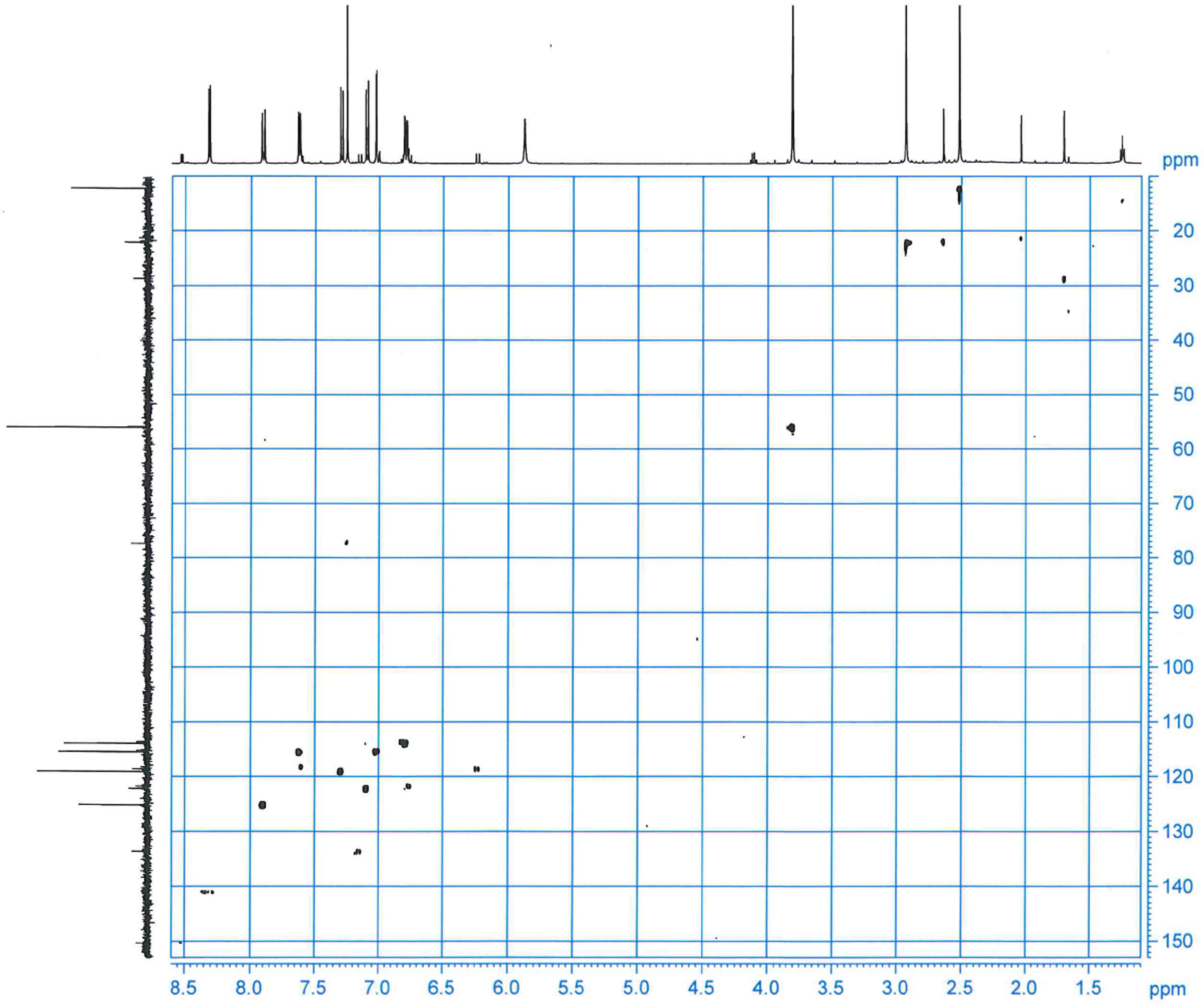

ppm
20
30
40
50
60
70
80
90
100
110
120
130
140
150
8.5
8.0
7.5
7.0
6.5
6.0
5.5
5.0
4.5
4.0
3.5
3.0
2.5
2.0
1.5
ppm

18c HSQC

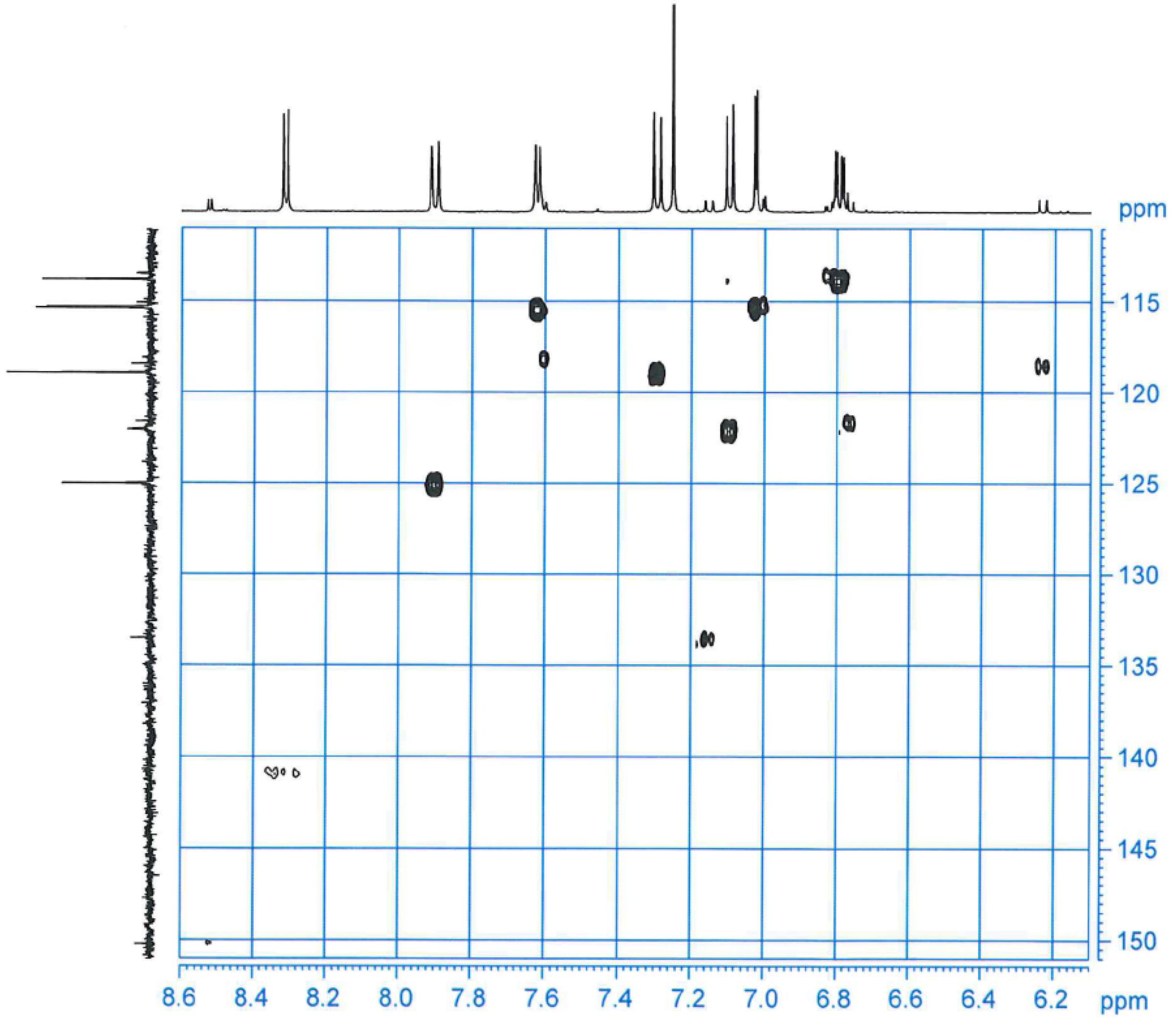

ppm
115
120
125
130
135
140
145
150
8.6
8.4
8.2
8.0
7.8
7.6
7.4
7.2
7.0
6.8
6.6
6.4
6.2
ppm

18c HMBC

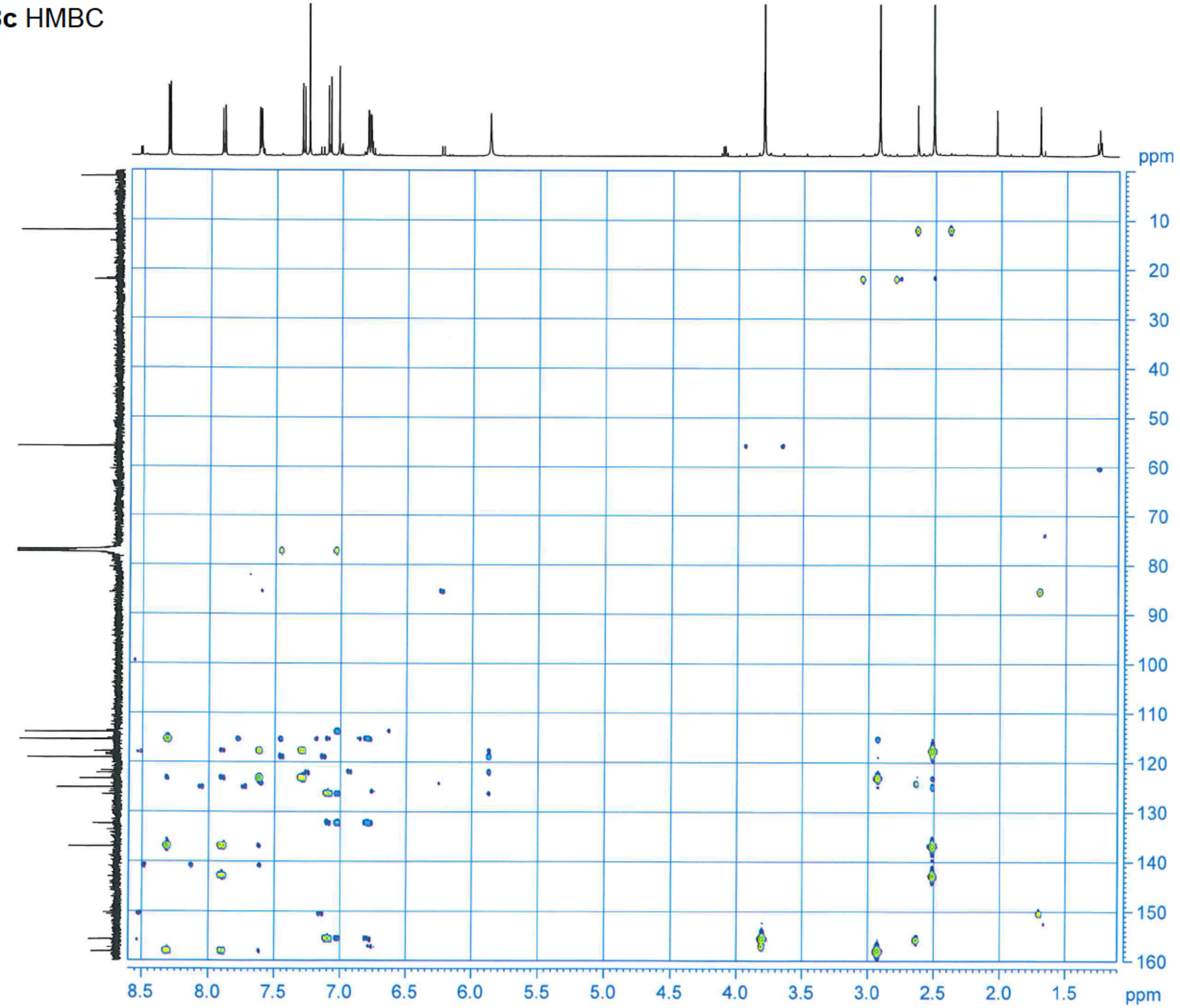
ppm
10
20
30
40
50
60
70
80
90
100
110
120
130
140
150
160
8.5 8.0 7.5 7.0 6.5 6.0 5.5 5.0 4.5 4.0 3.5 3.0 2.5 2.0 1.5
ppm

18c HMBC

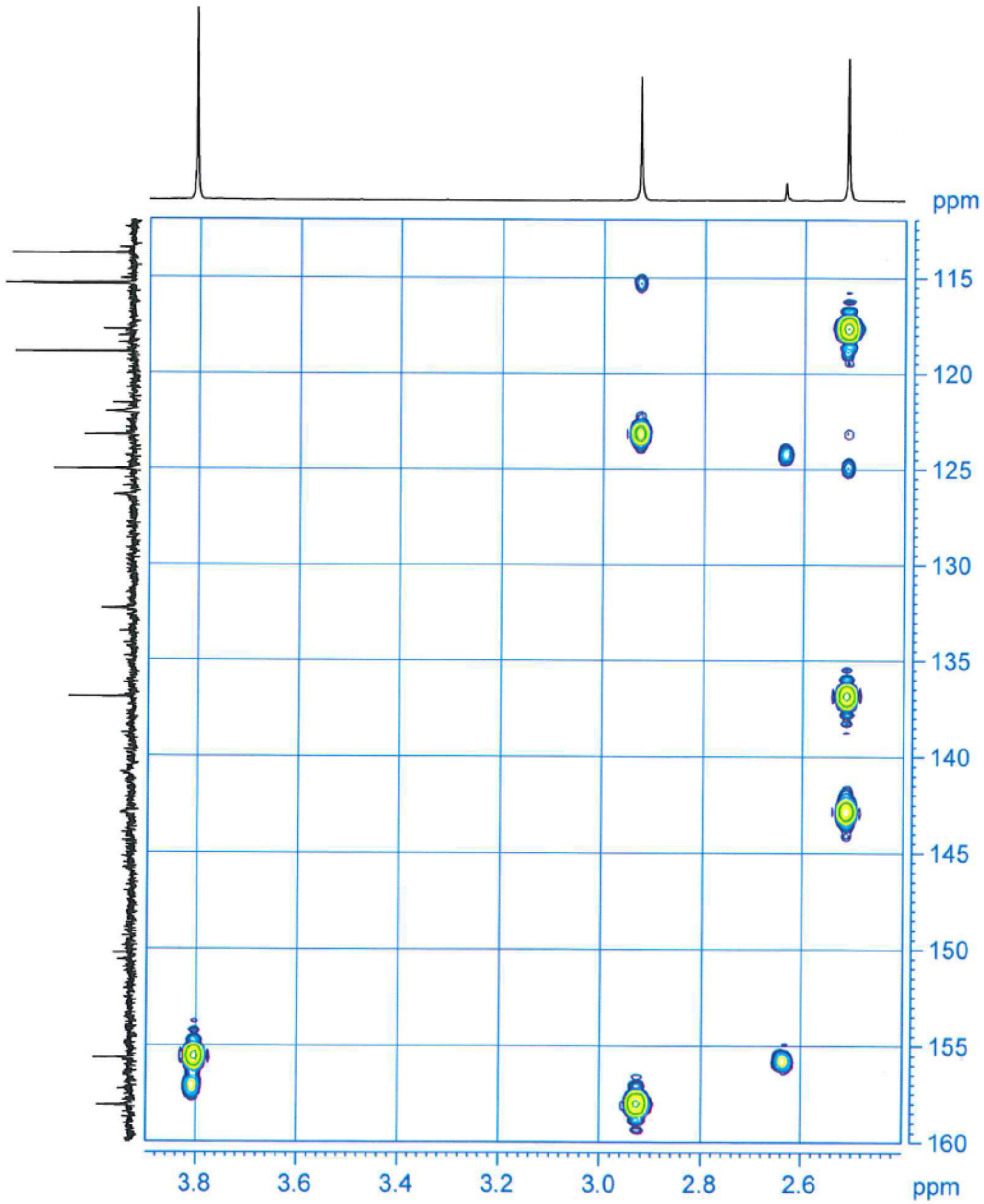

ppm
115
120
125
130
135
140
145
150
155
160
3.8
3.6
3.4
3.2
3.0
2.8
2.6
ppm

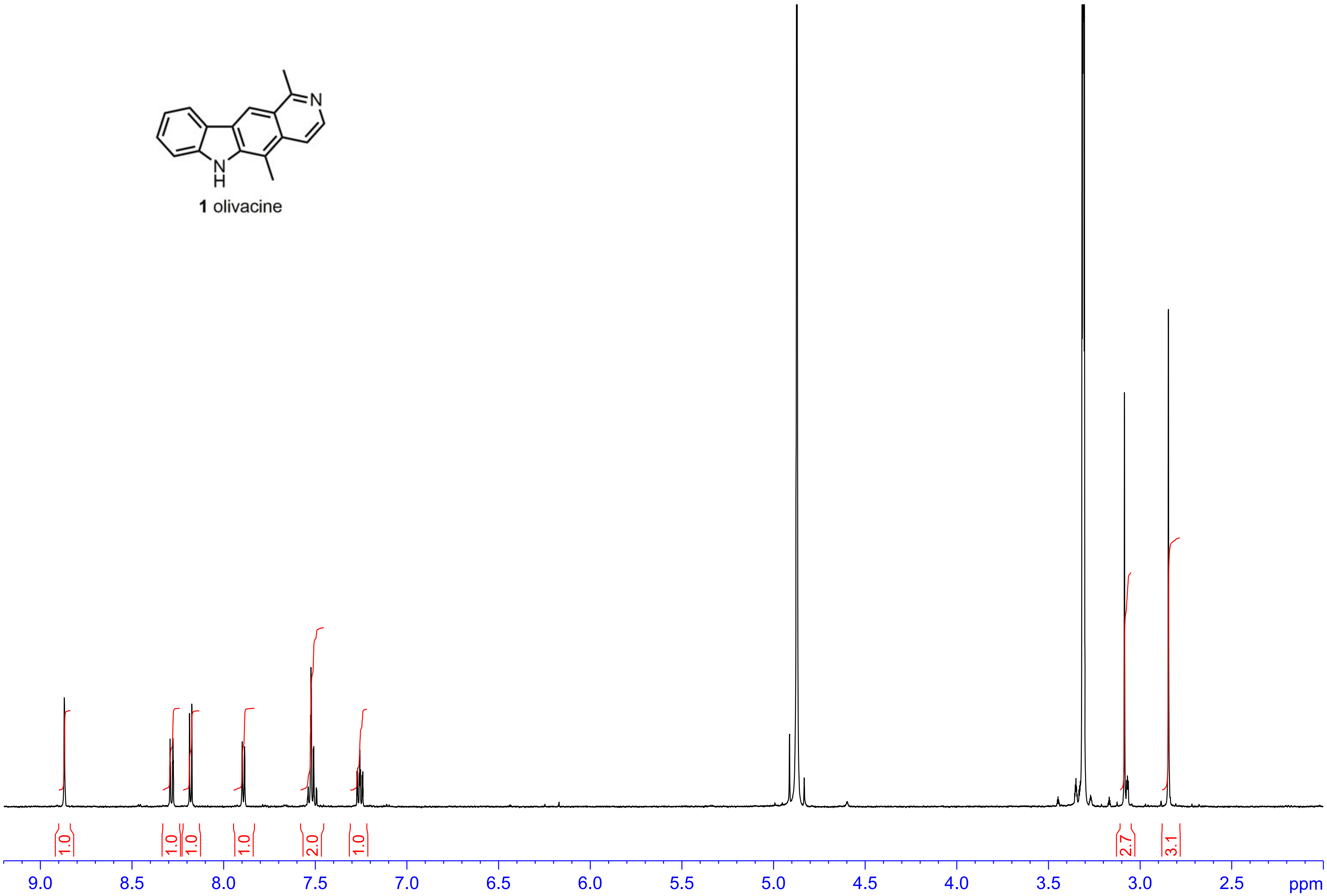
N
N
H
1.0
1.0
1.0
1.0
2.0
1.0
2.7
3.1
9.0
8.5
8.0
7.5
7.0
6.5
6.0
5.5
5.0
4.5
4.0
3.5
3.0
2.5
ppm

1 olivacine

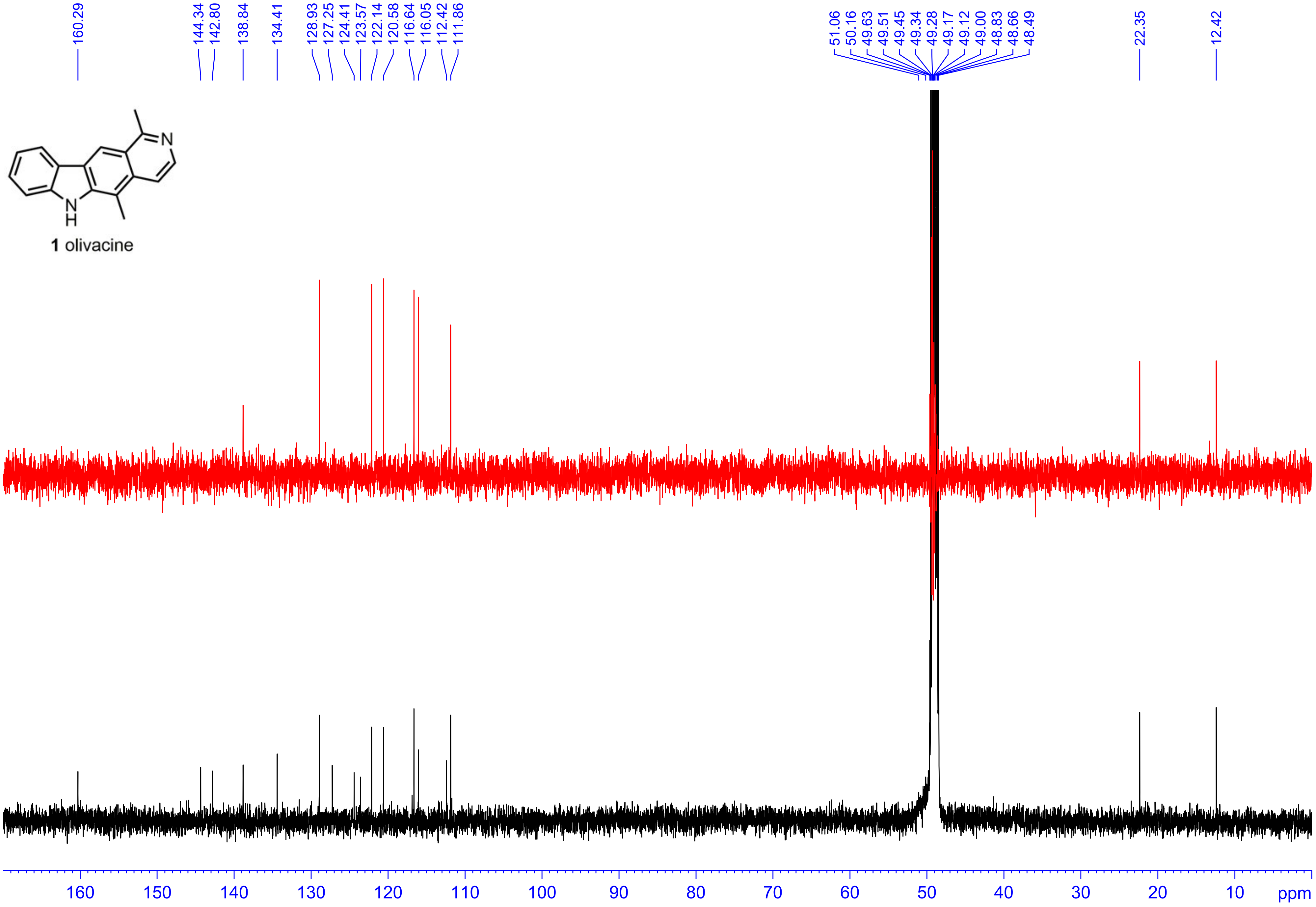
160.29
144.34
142.80
138.84
134.41
128.93
127.25
124.41
123.57
122.14
120.58
116.64
116.05
112.42
111.86
51.06
50.16
49.63
49.51
49.45
49.34
49.28
49.17
49.12
49.00
48.83
48.66
48.49
22.35
12.42
N
N
H
1 olivacine
160
150
140
130
120
110
100
90
80
70
60
50
40
30
20
10
ppm

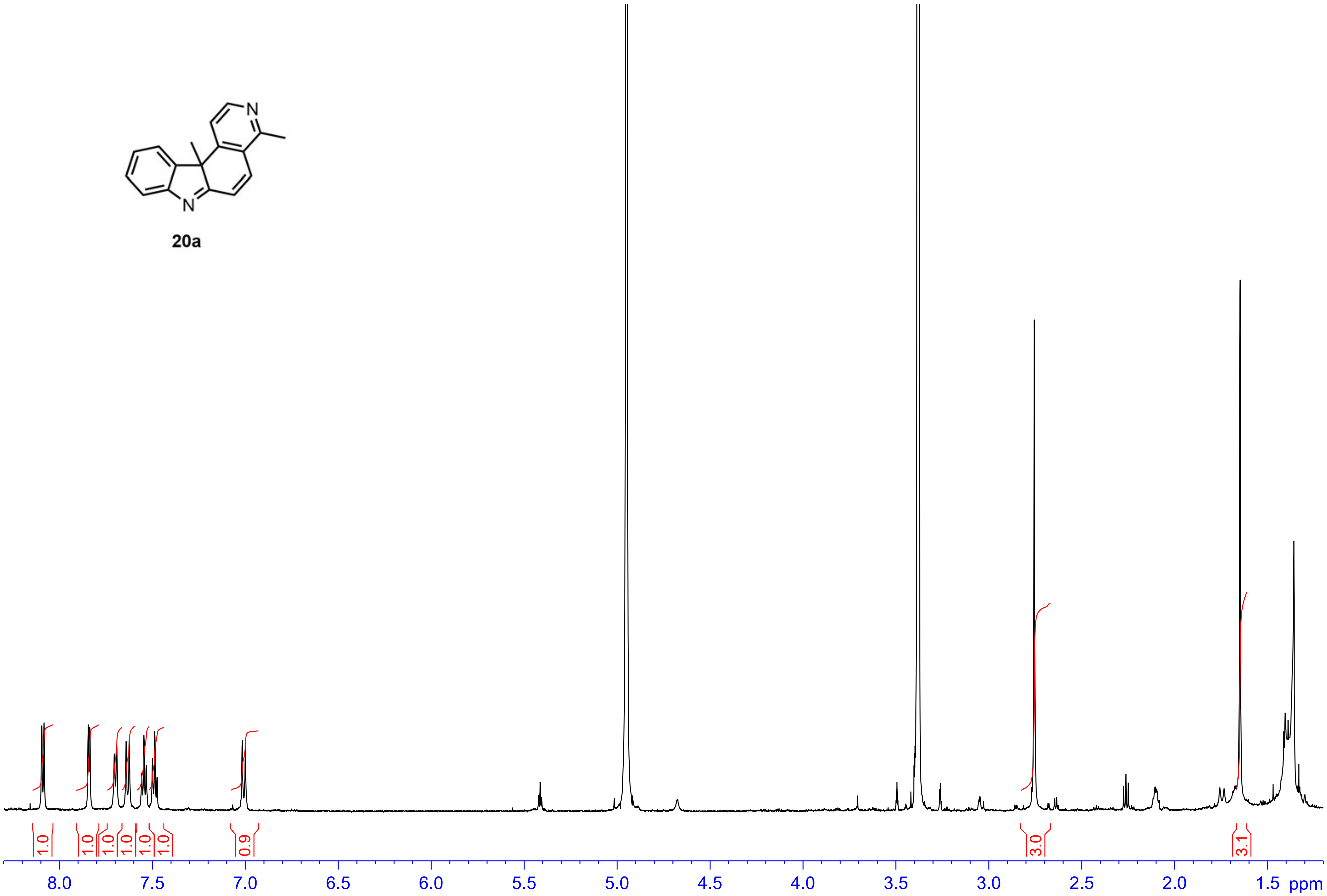

20a

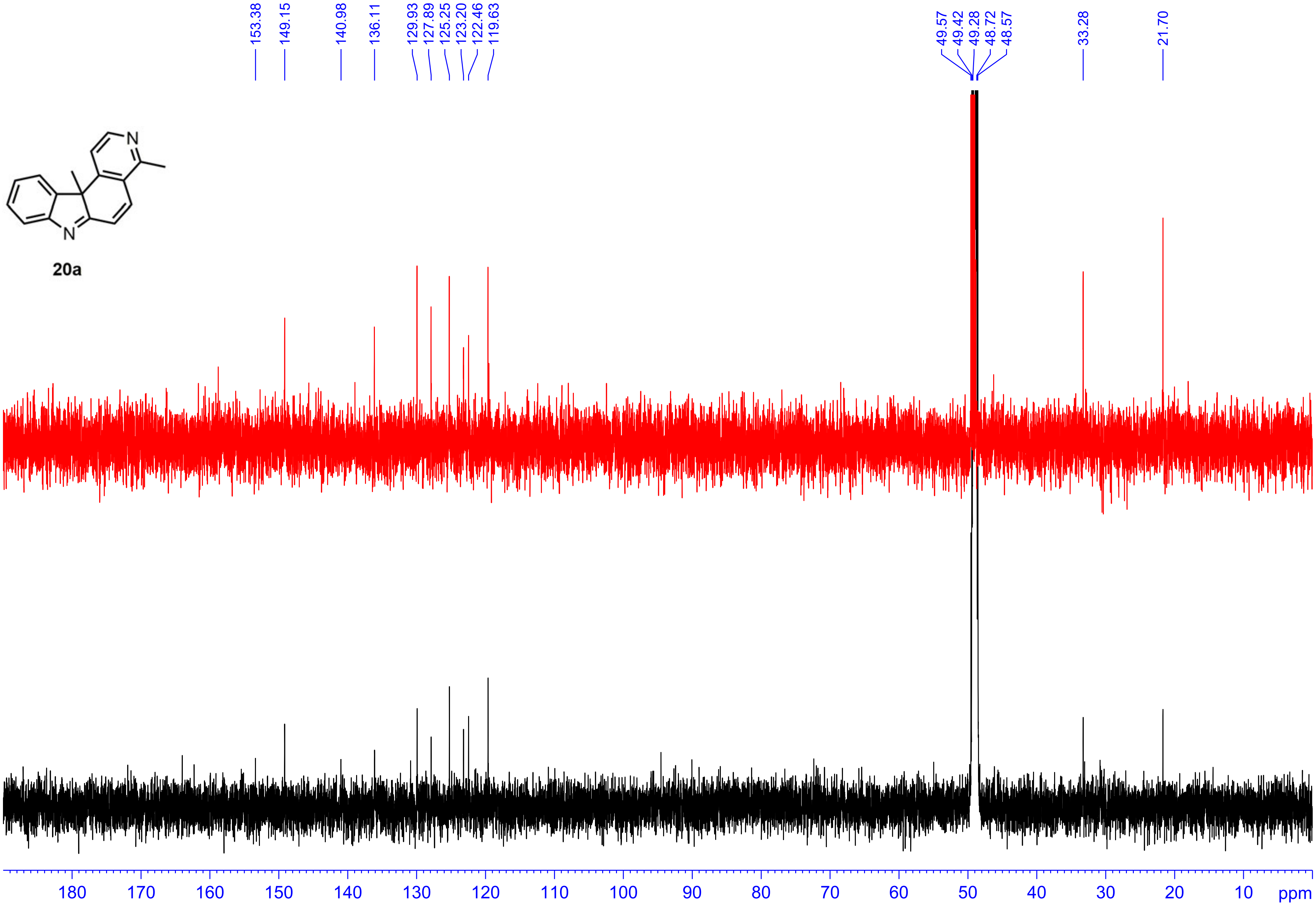
20a
153.38
149.15
140.98
136.11
129.93
127.89
125.25
123.20
122.46
119.63
49.57
49.42
49.28
48.72
48.57
33.28
21.70
180
170
160
150
140
130
120
110
100
90
80
70
60
50
40
30
20
10
ppm

20a COSY

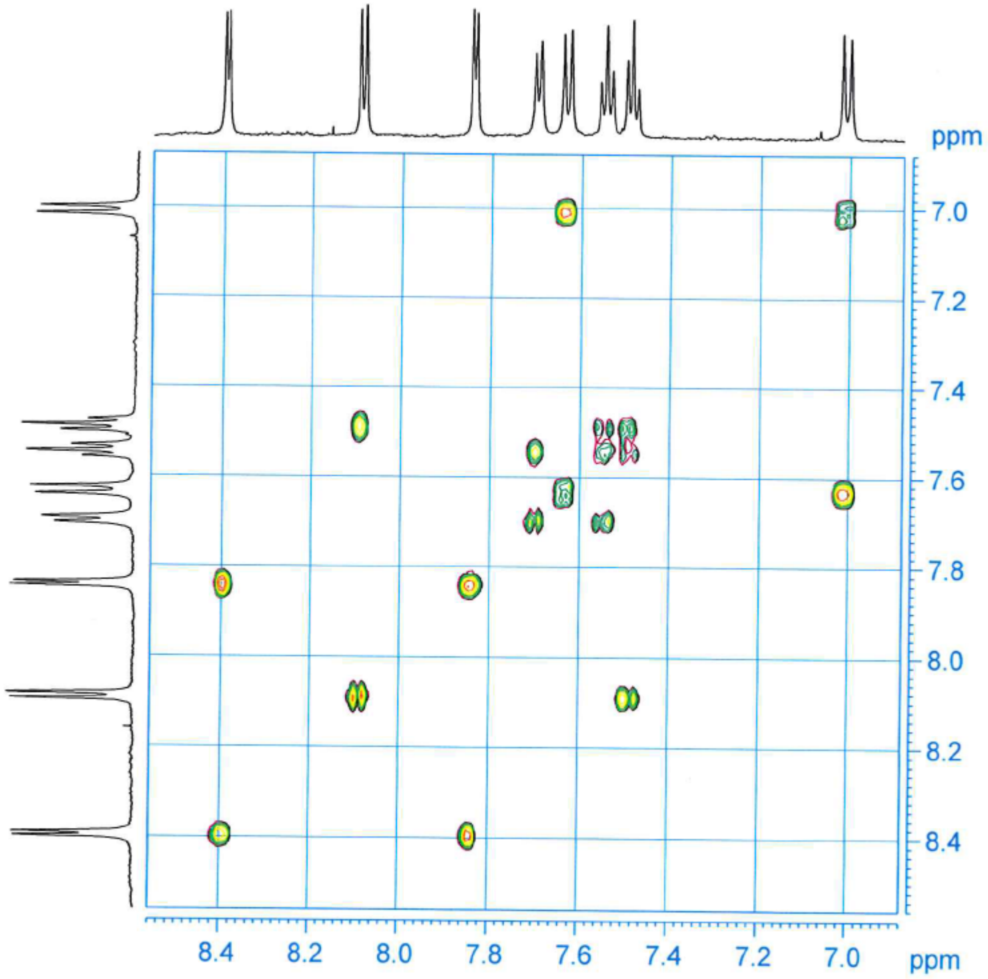

20a HSQC

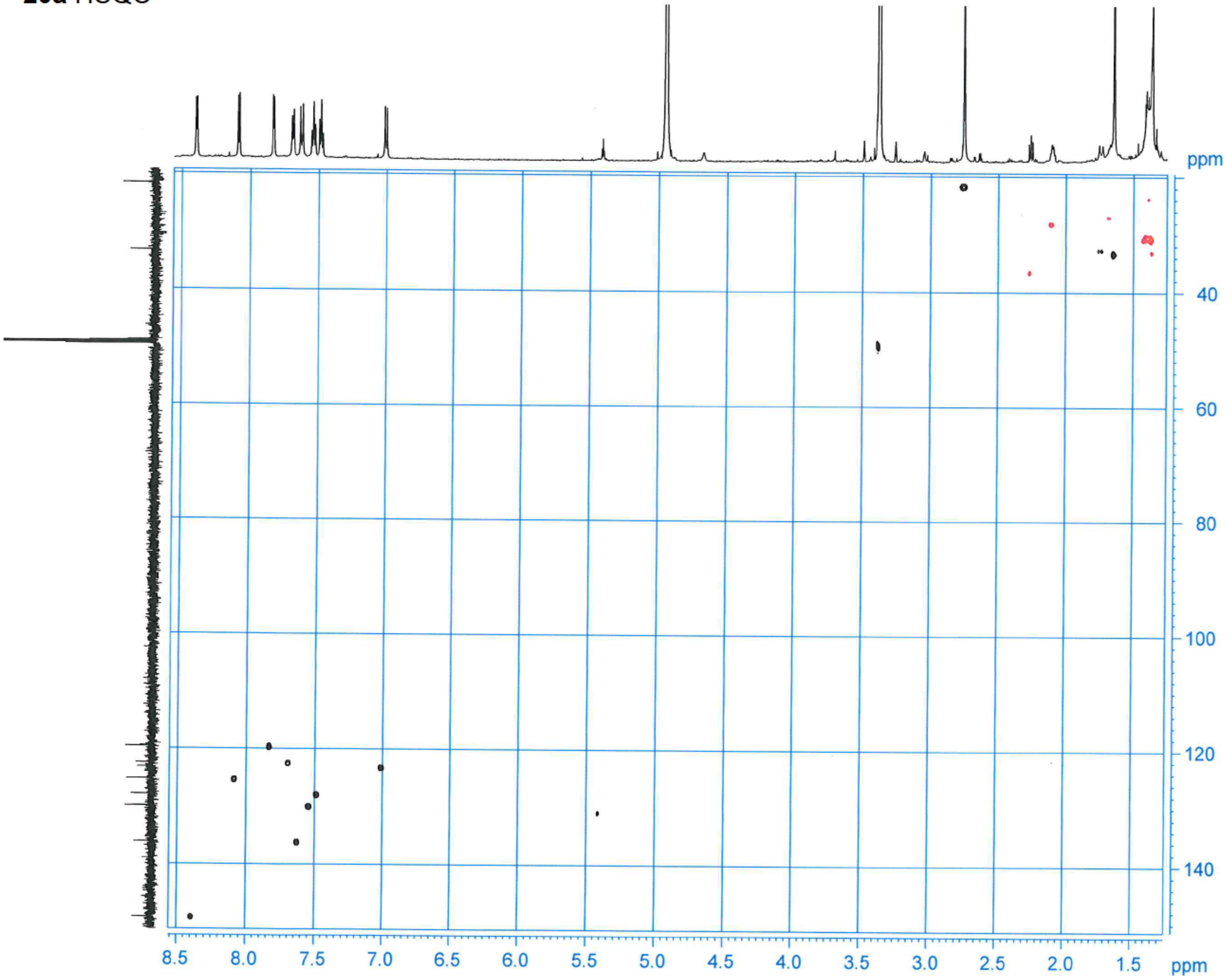

ppm
40
60
80
100
120
140
8.5
8.0
7.5
7.0
6.5
6.0
5.5
5.0
4.5
4.0
3.5
3.0
2.5
2.0
1.5
ppm

20a HMBC

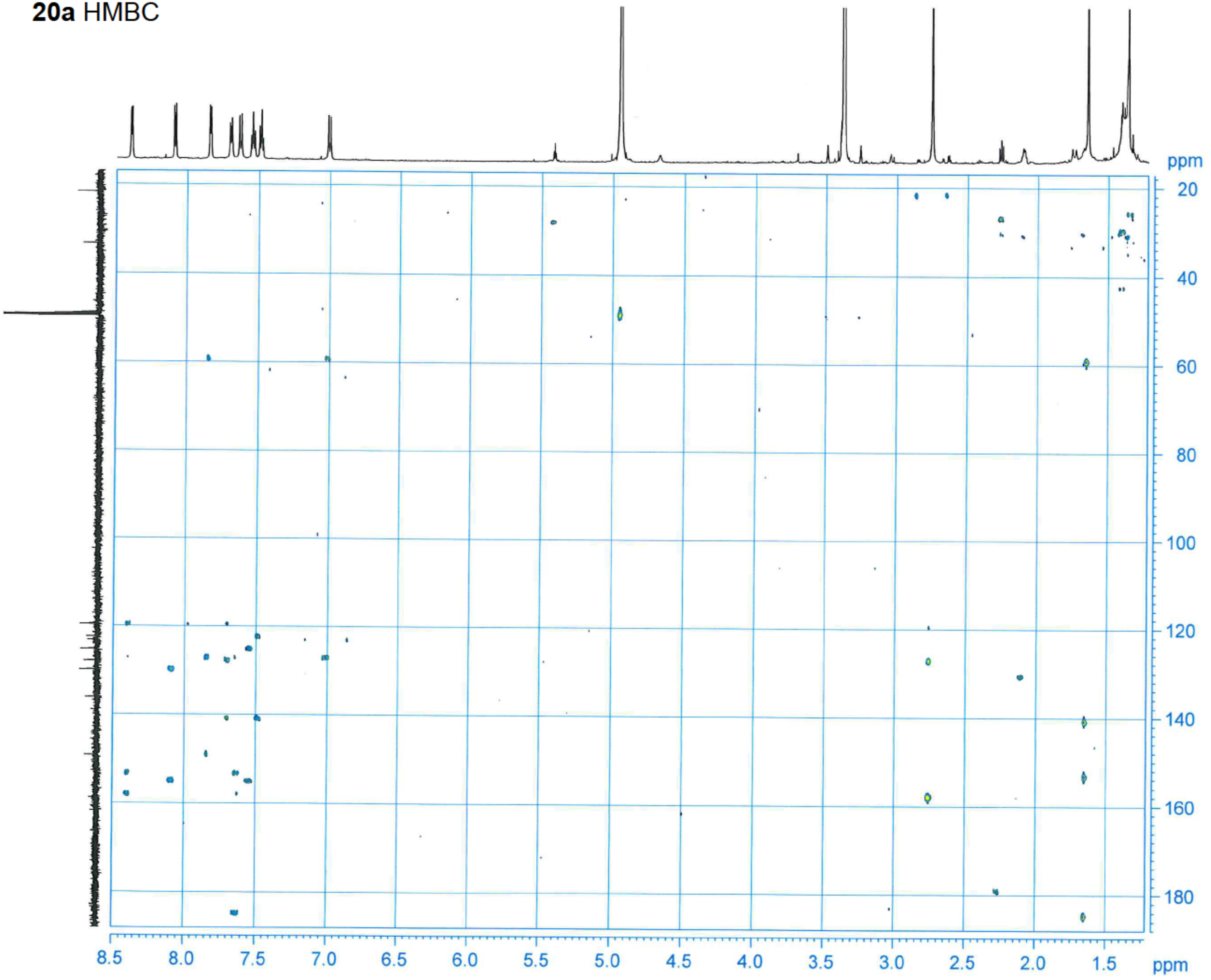

ppm
20
40
60
80
100
120
140
160
180
8.5
8.0
7.5
7.0
6.5
6.0
5.5
5.0
4.5
4.0
3.5
3.0
2.5
2.0
1.5
ppm

20a NOESY

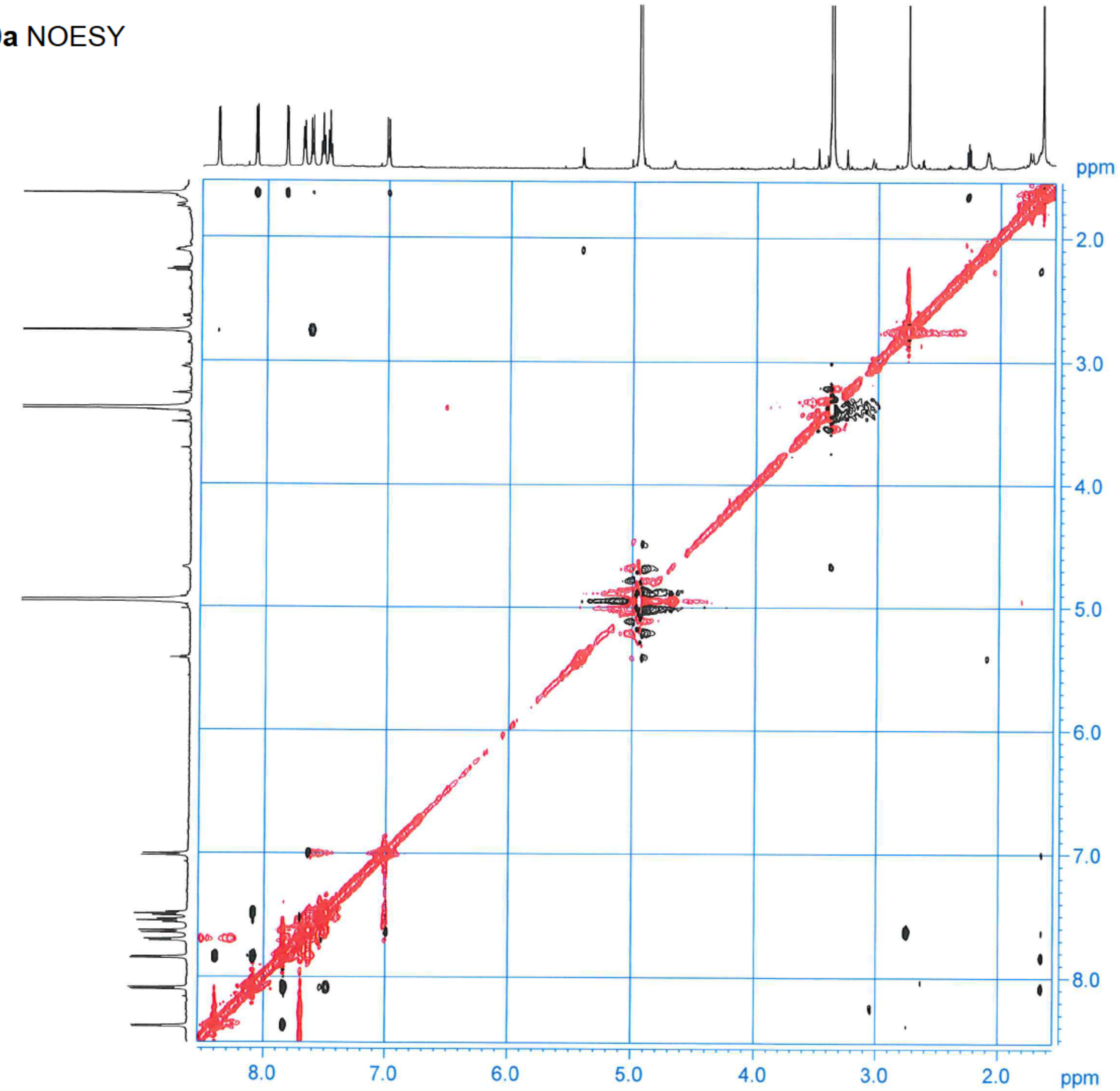

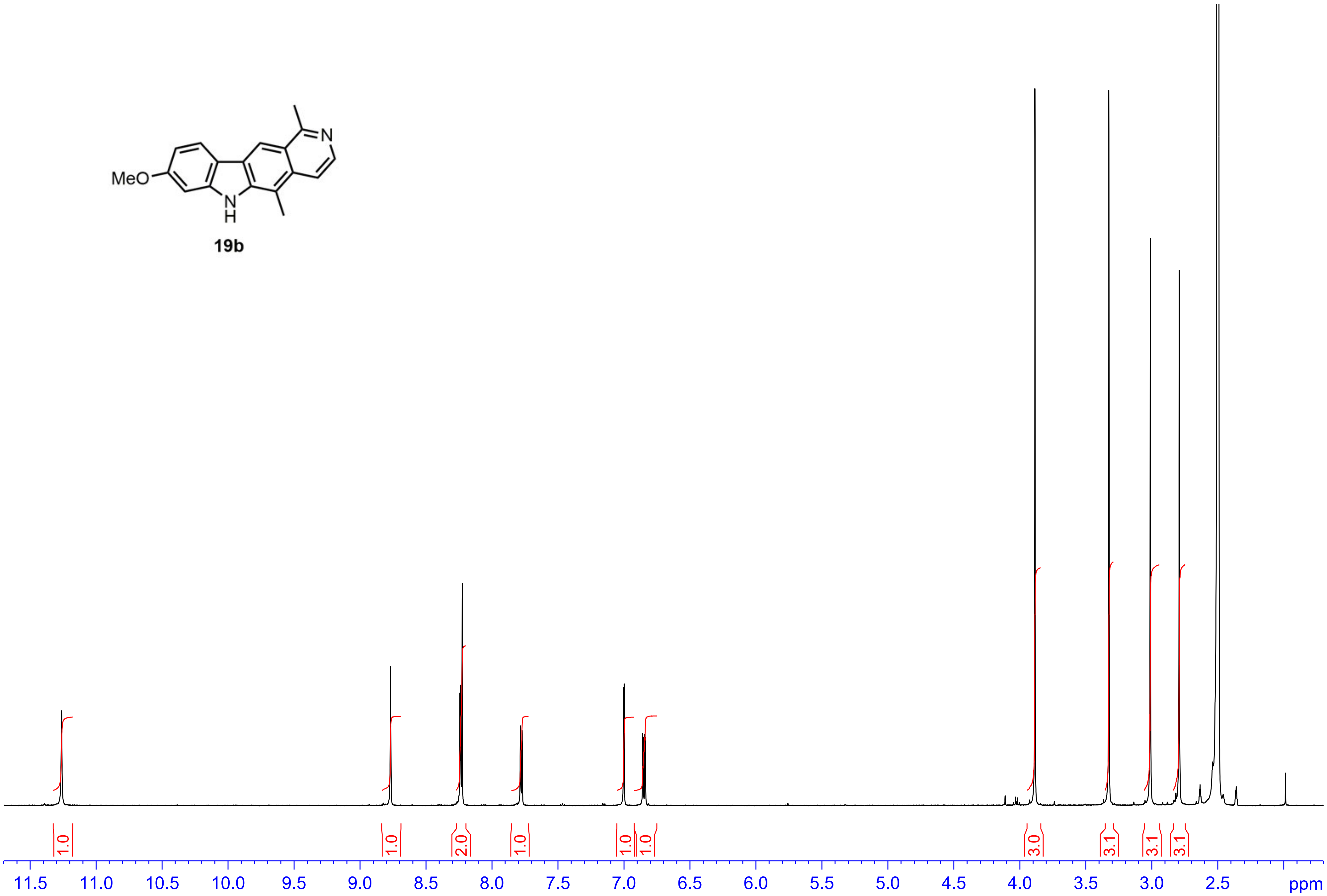
MeO
N
H
N
1.0
1.0
2.0
1.0
1.0
1.0
3.0
3.1
3.1
3.1
11.5
11.0
10.5
10.0
9.5
9.0
8.5
8.0
7.5
7.0
6.5
6.0
5.5
5.0
4.5
4.0
3.5
3.0
2.5
ppm

19b

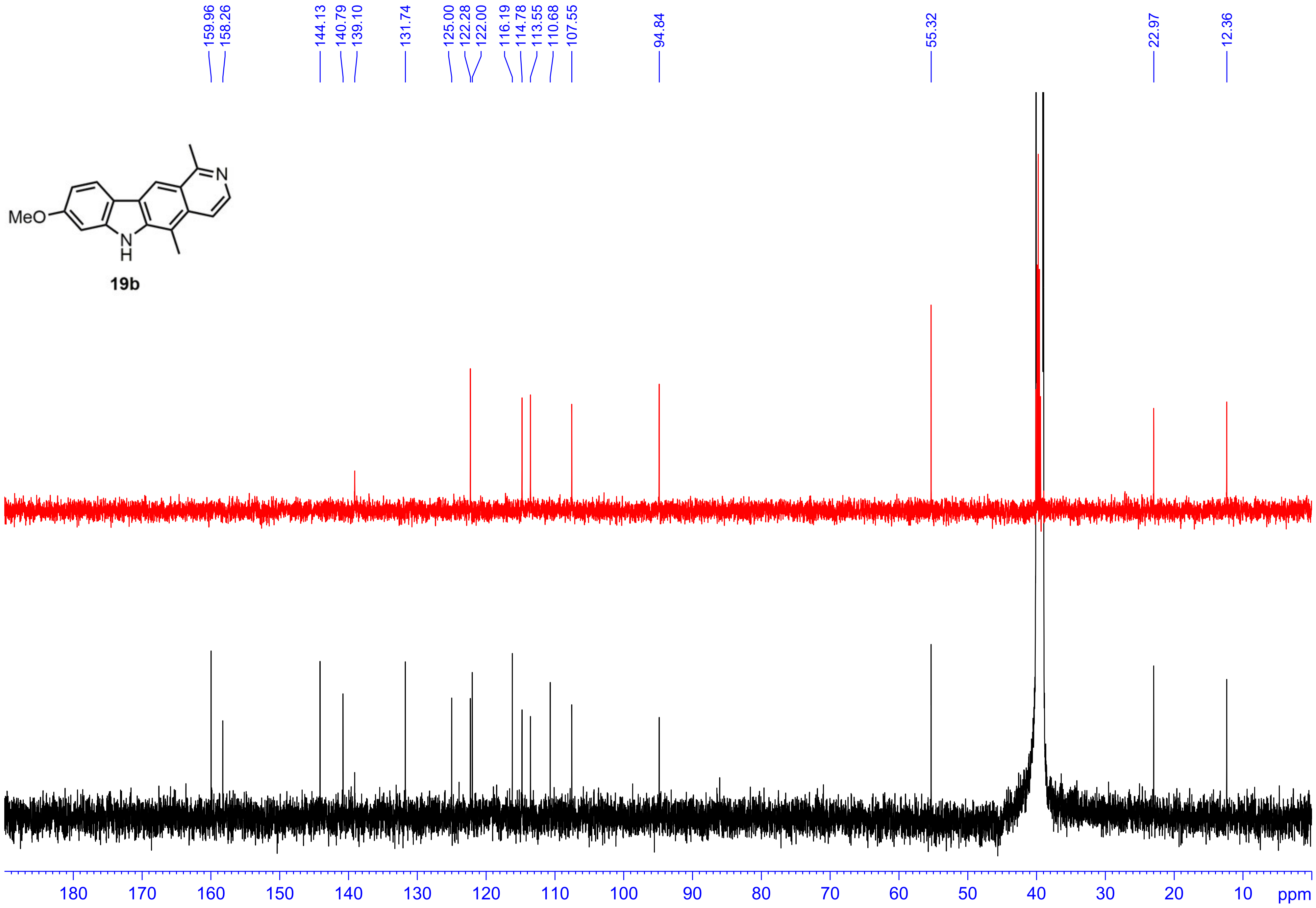
159.96
158.26
144.13
140.79
139.10
131.74
125.00
122.28
122.00
116.19
114.78
113.55
110.68
107.55
94.84
55.32
22.97
12.36
MeO
N
N
H
180
170
160
150
140
130
120
110
100
90
80
70
60
50
40
30
20
10
ppm

19b

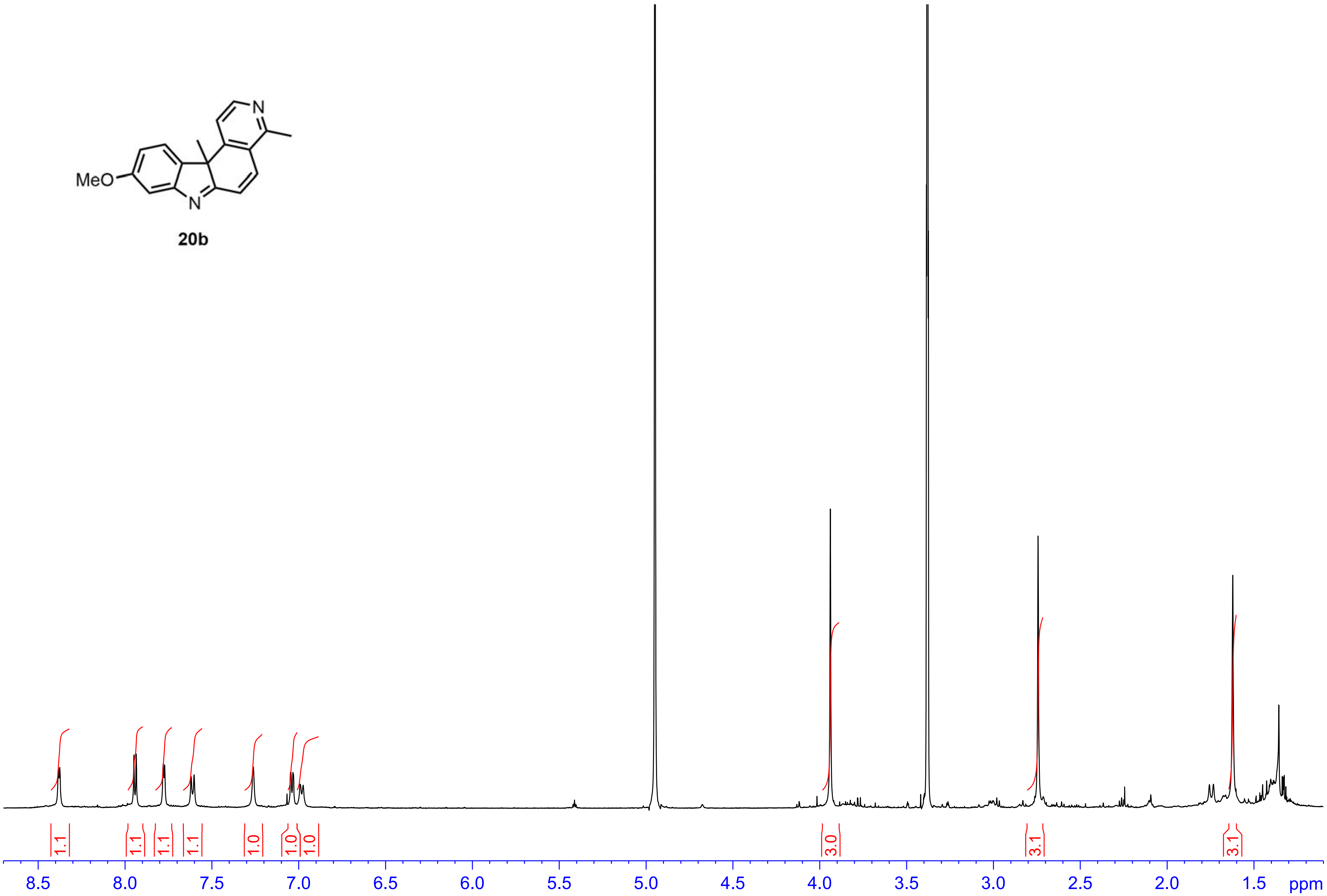
MeO
N
N
20b
1.1
1.1
1.1
1.1
1.0
1.0
1.0
3.0
3.1
3.1
8.5
8.0
7.5
7.0
6.5
6.0
5.5
5.0
4.5
4.0
3.5
3.0
2.5
2.0
1.5
ppm

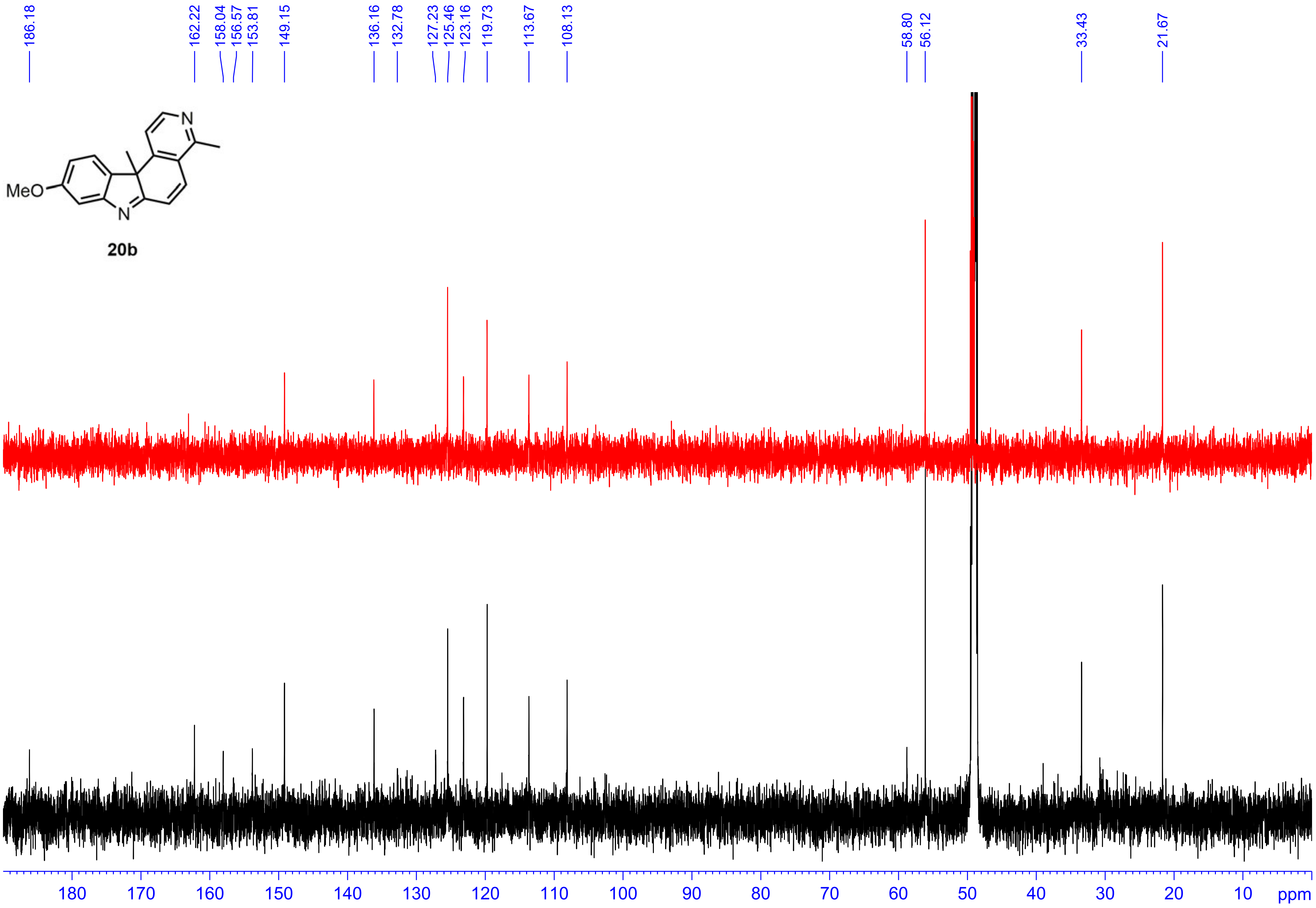
186.18
162.22
158.04
156.57
153.81
149.15
136.16
132.78
127.23
125.46
123.16
119.73
113.67
108.13
58.80
56.12
33.43
21.67
MeO
N
N
20b
180
170
160
150
140
130
120
110
100
90
80
70
60
50
40
30
20
10
ppm

20b COSY

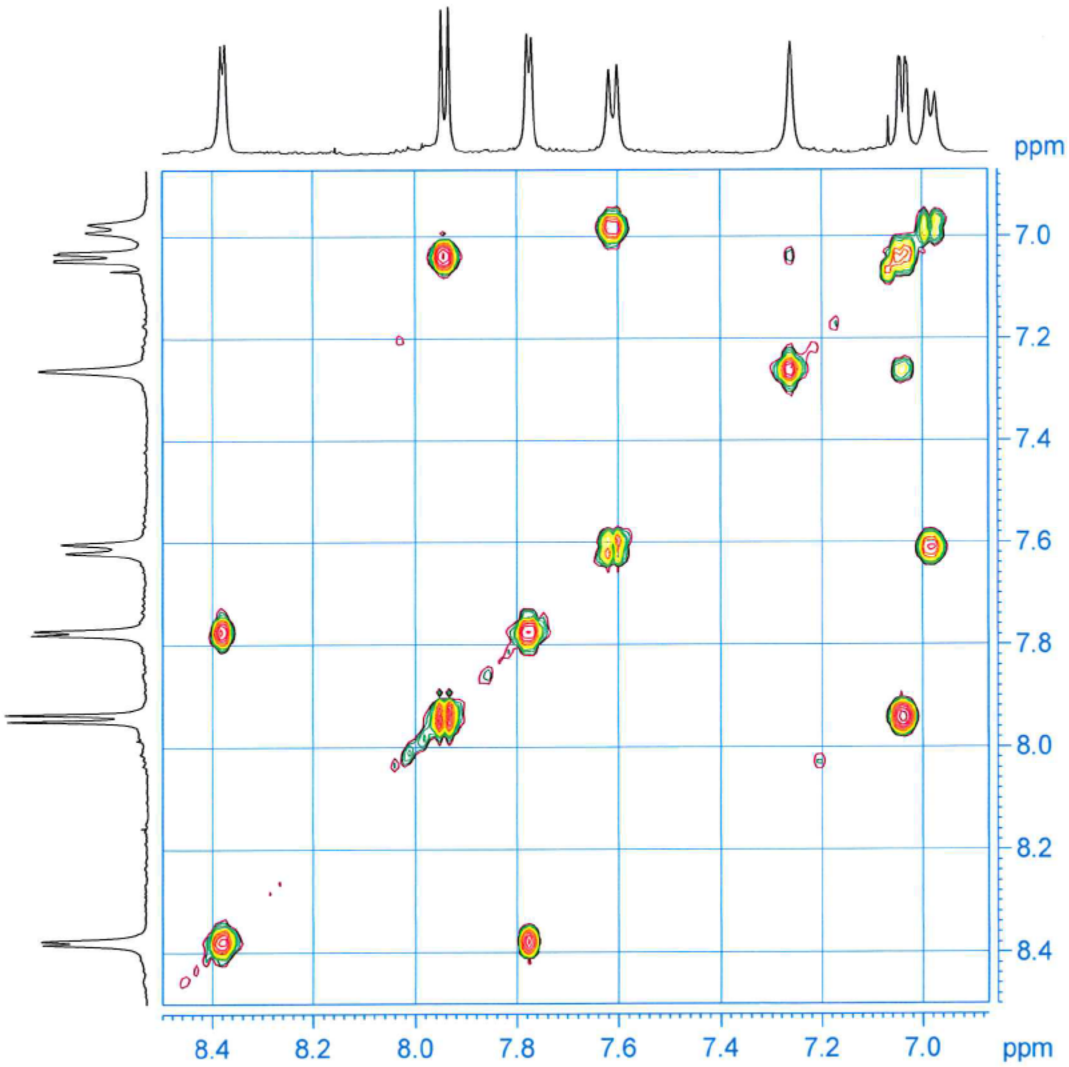

20b HSQC

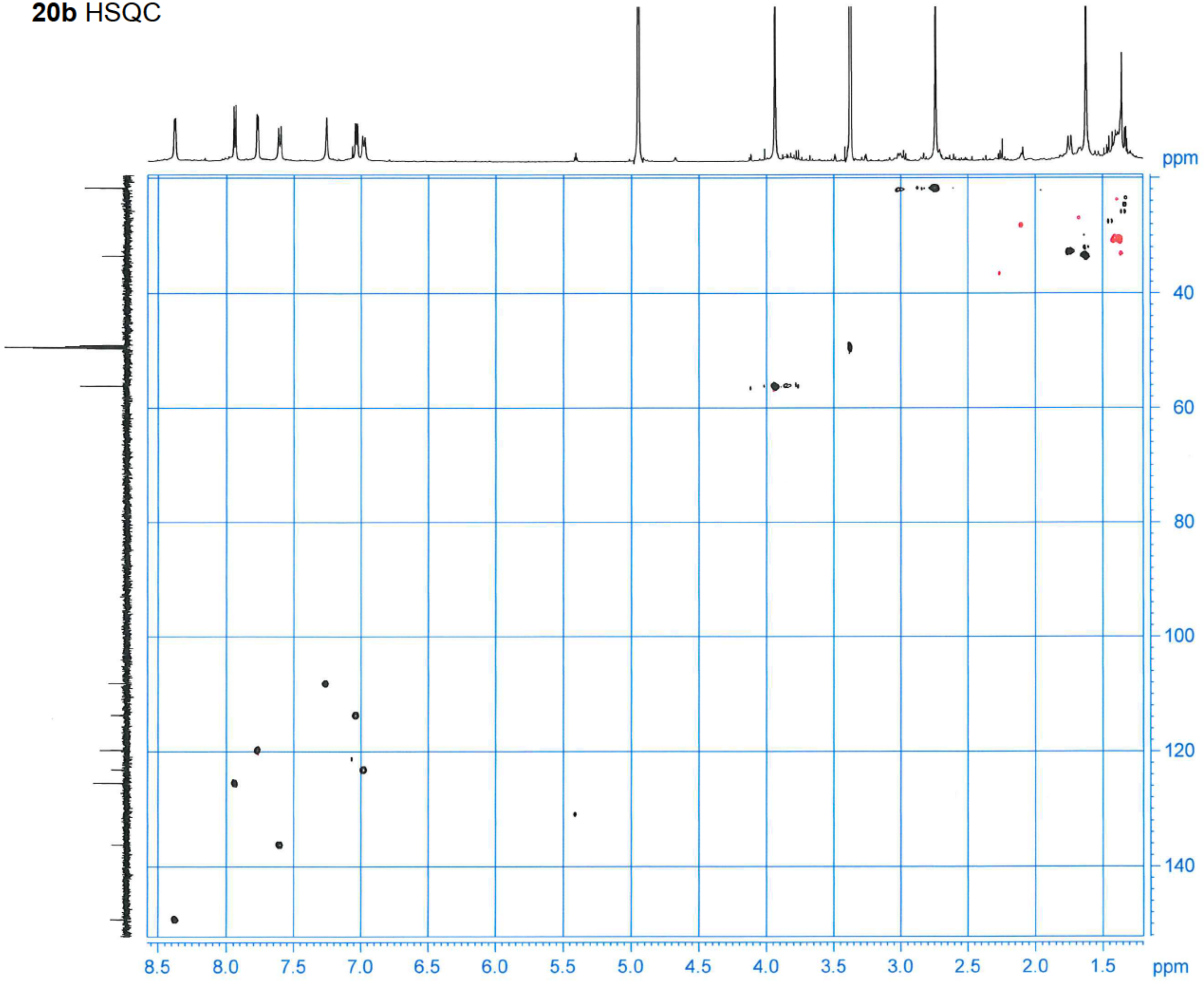
ppm
40
60
80
100
120
140
8.5 8.0 7.5 7.0 6.5 6.0 5.5 5.0 4.5 4.0 3.5 3.0 2.5 2.0 1.5
ppm

20b HMBC

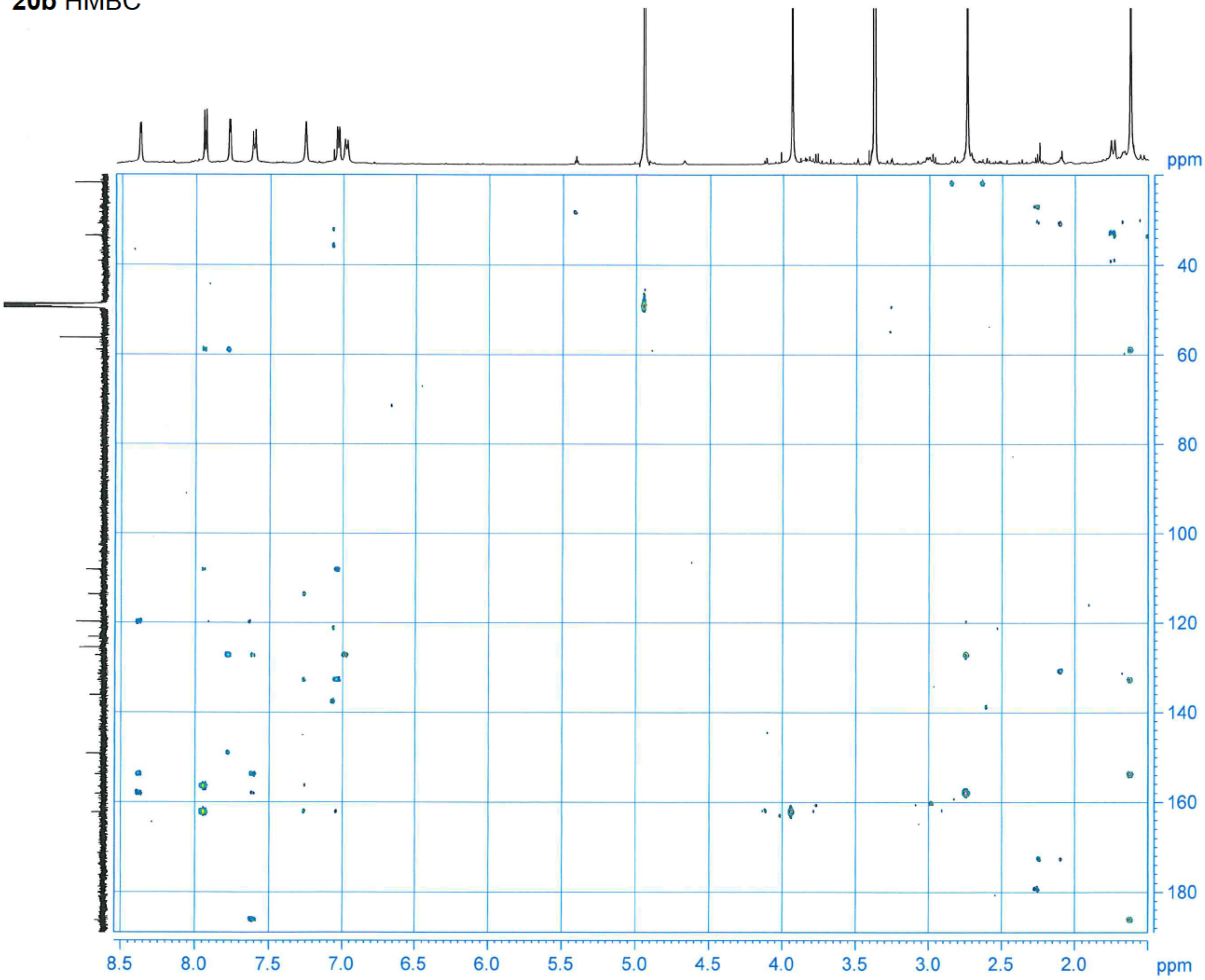
ppm
40
60
80
100
120
140
160
180
8.5
8.0
7.5
7.0
6.5
6.0
5.5
5.0
4.5
4.0
3.5
3.0
2.5
2.0
ppm

20b NOESY

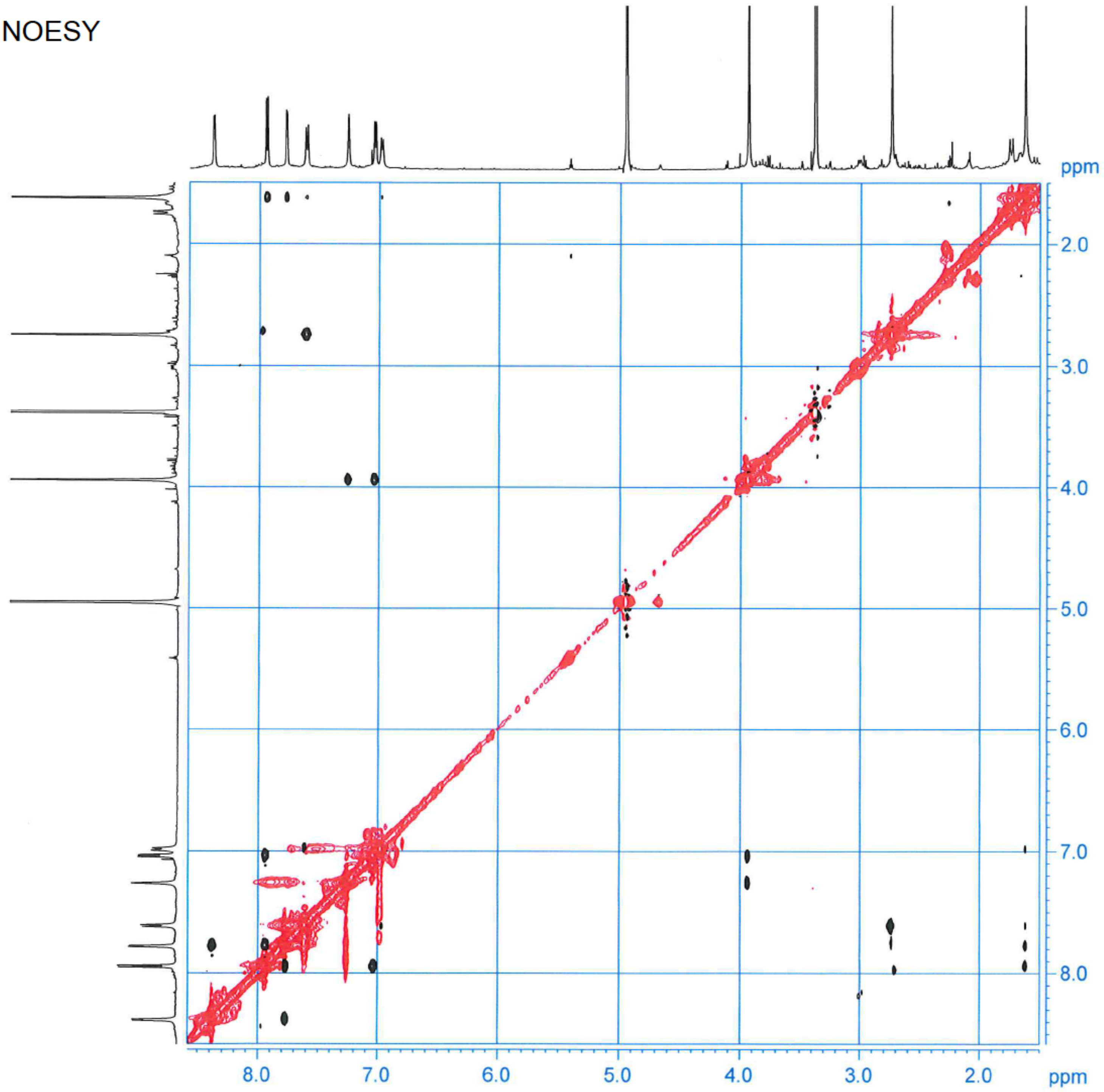

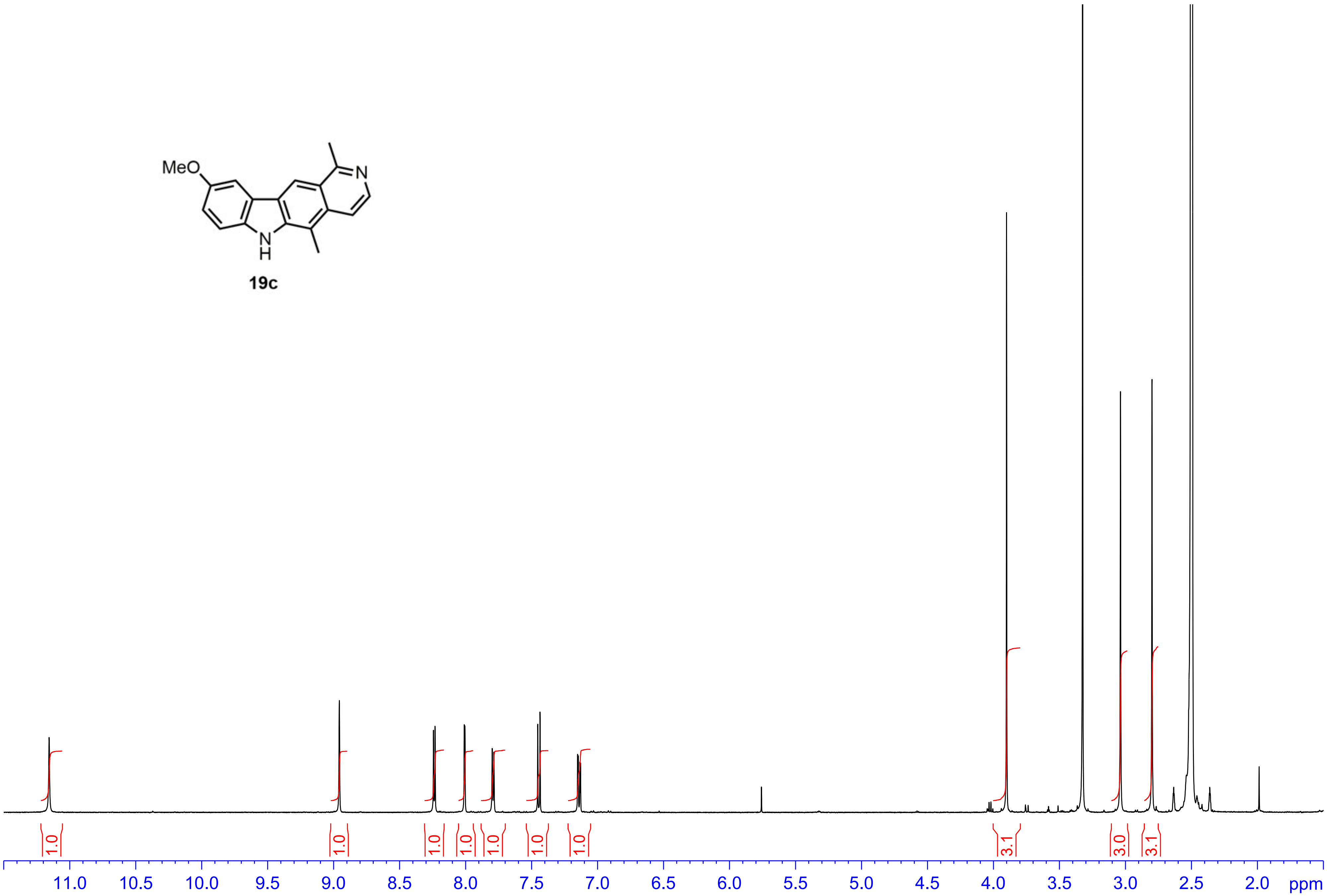
MeO
N
N
H
1.0
1.0
1.0
1.0
1.0
1.0
1.0
3.1
3.0
3.1
11.0
10.5
10.0
9.5
9.0
8.5
8.0
7.5
7.0
6.5
6.0
5.5
5.0
4.5
4.0
3.5
3.0
2.5
2.0
ppm

19c

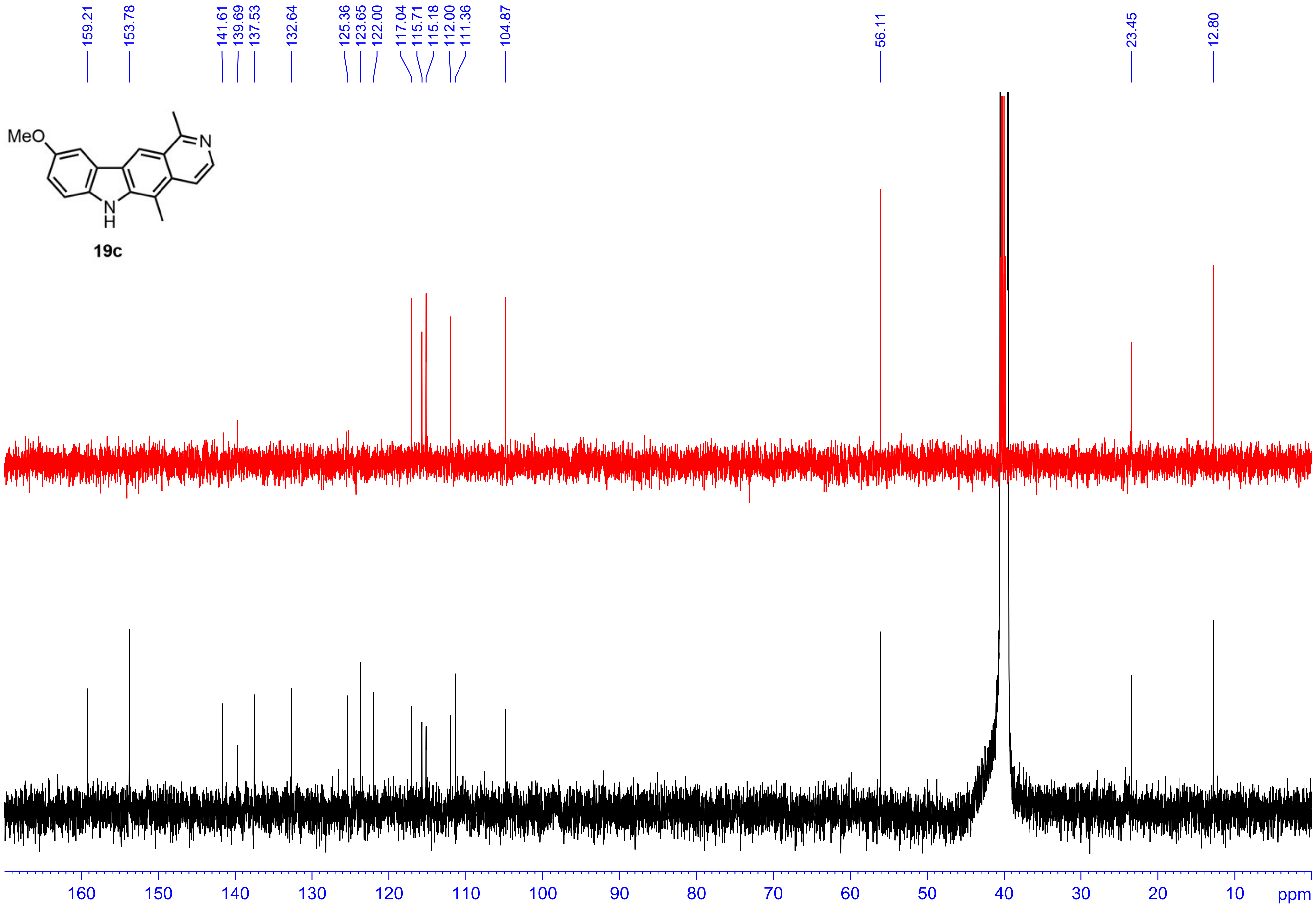

159.21
153.78
141.61
139.69
137.53
132.64
125.36
123.65
122.00
117.04
115.71
115.18
112.00
111.36
104.87
56.11
23.45
12.80
MeO
N
N
H
19c
160
150
140
130
120
110
100
90
80
70
60
50
40
30
20
10
ppm

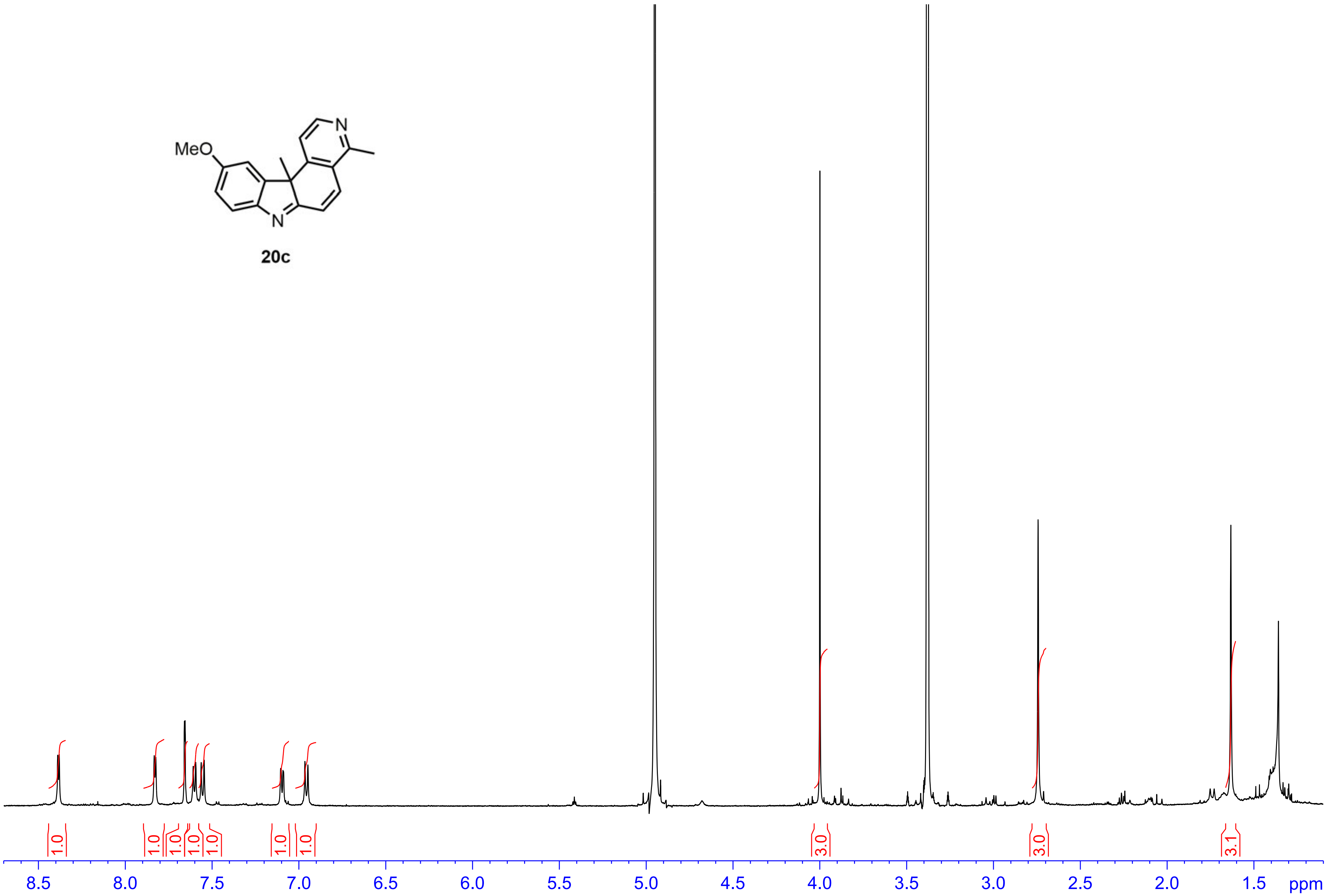
MeO
N
N
1.0
1.0
1.0
1.0
1.0
1.0
1.0
3.0
3.0
3.1
8.5
8.0
7.5
7.0
6.5
6.0
5.5
5.0
4.5
4.0
3.5
3.0
2.5
2.0
1.5
ppm

20c

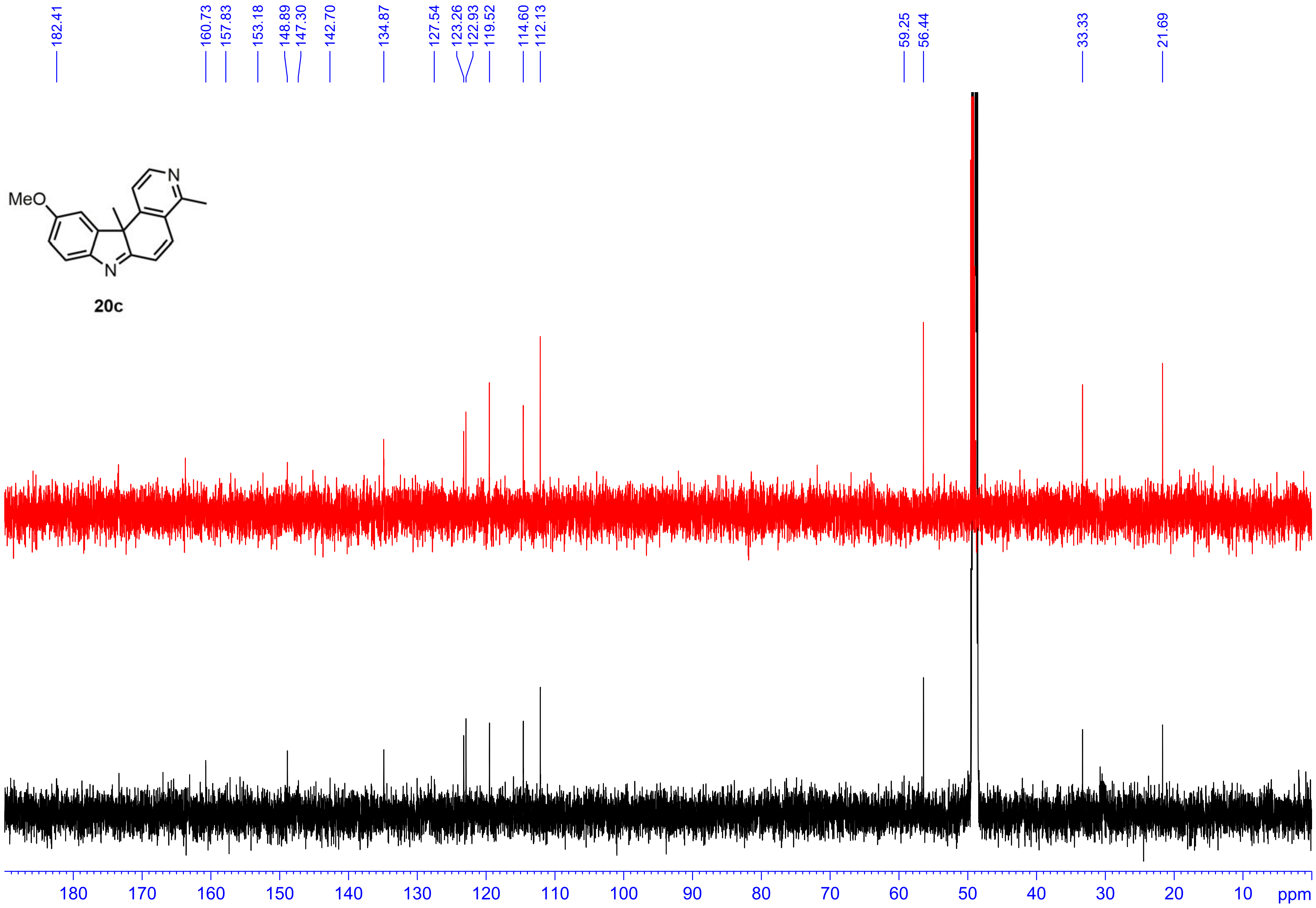
182.41
160.73
157.83
153.18
148.89
147.30
142.70
134.87
127.54
123.26
122.93
119.52
114.60
112.13
59.25
56.44
33.33
21.69
MeO
N
N
180 170 160 150 140 130 120 110 100 90 80 70 60 50 40 30 20 10 ppm

20c

20c COSY

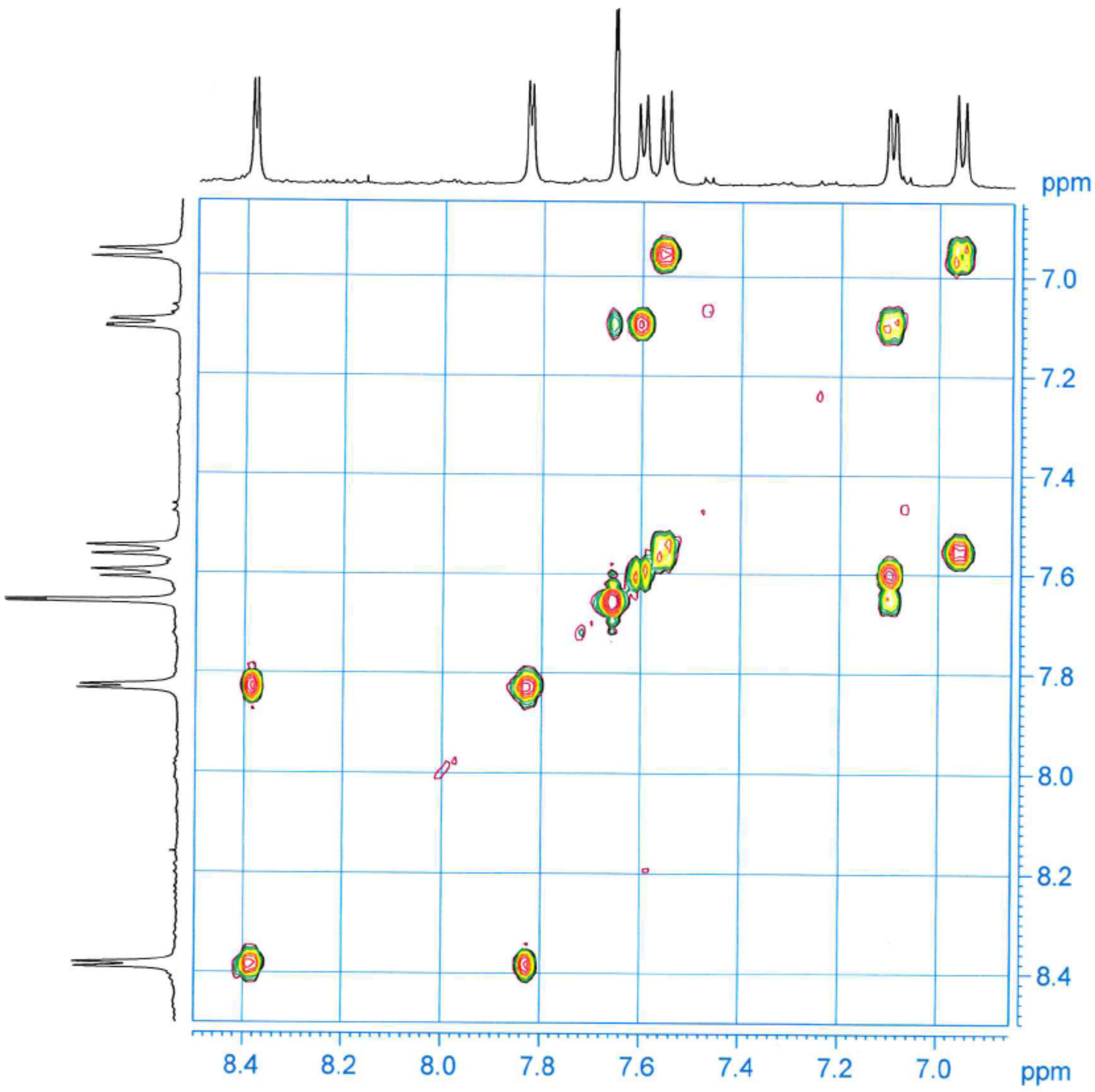

20c HSQC

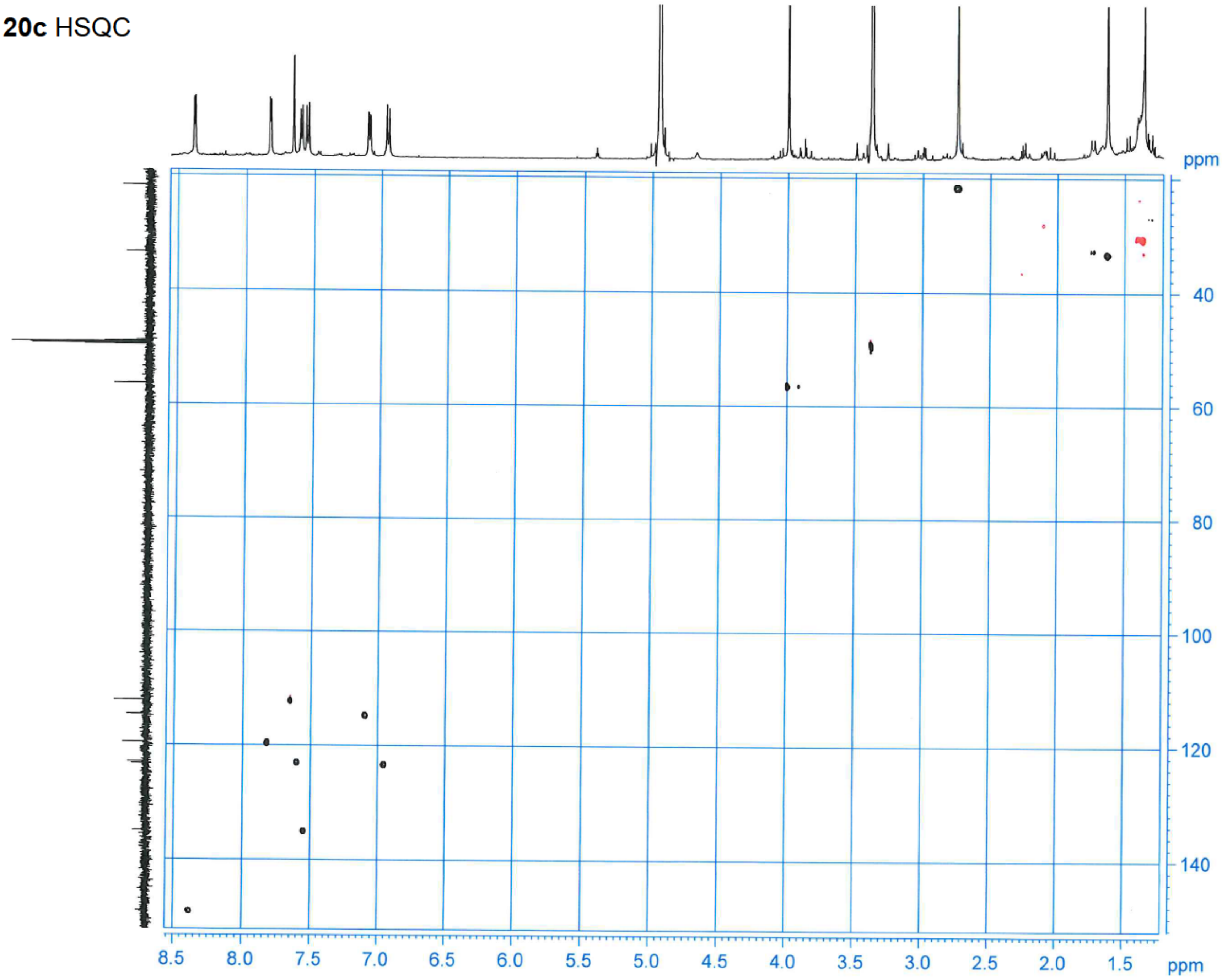
ppm
40
60
80
100
120
140
8.5 8.0 7.5 7.0 6.5 6.0 5.5 5.0 4.5 4.0 3.5 3.0 2.5 2.0 1.5 ppm

20c HMBC

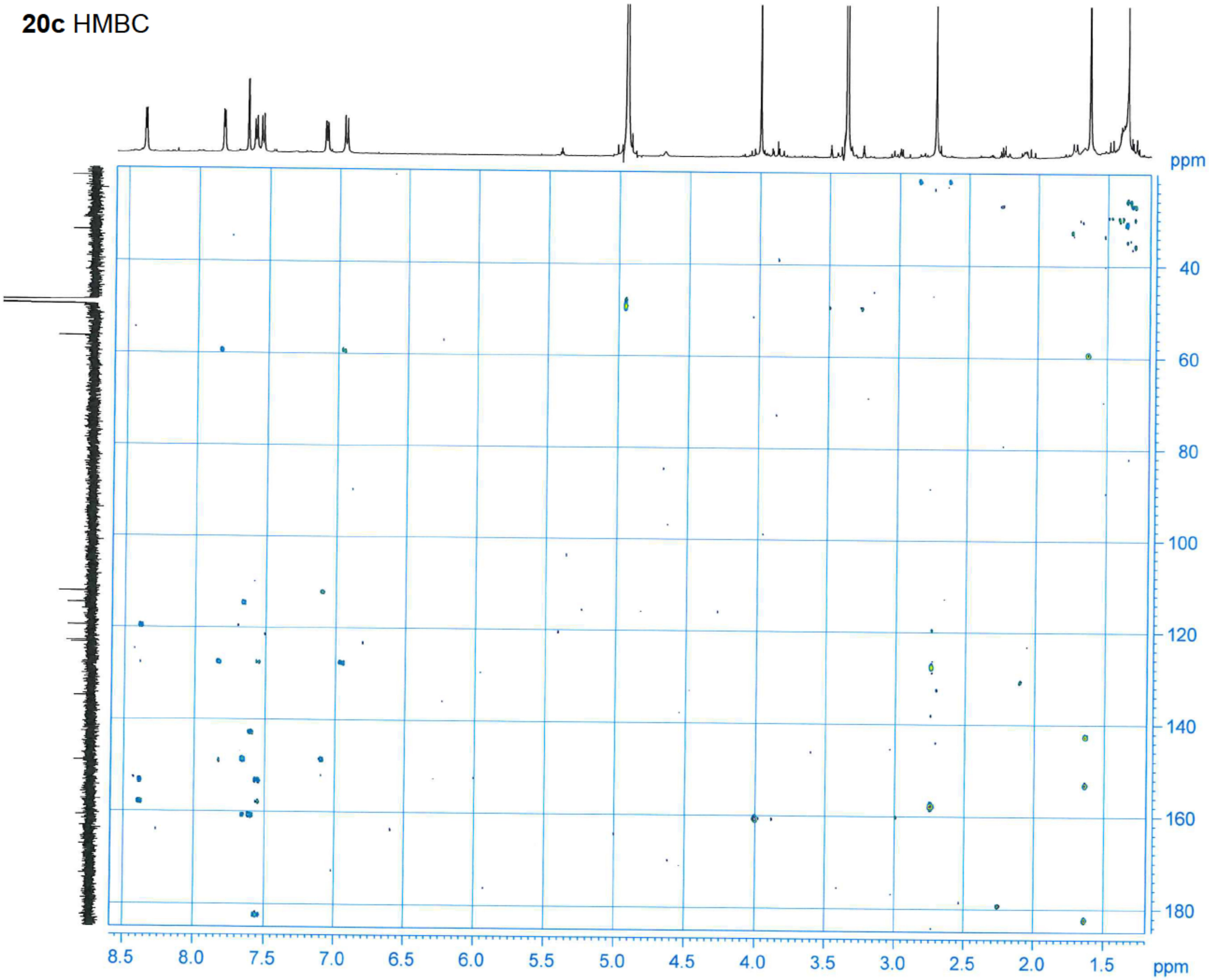

ppm
40
60
80
100
120
140
160
180
8.5
8.0
7.5
7.0
6.5
6.0
5.5
5.0
4.5
4.0
3.5
3.0
2.5
2.0
1.5
ppm

20c NOESY

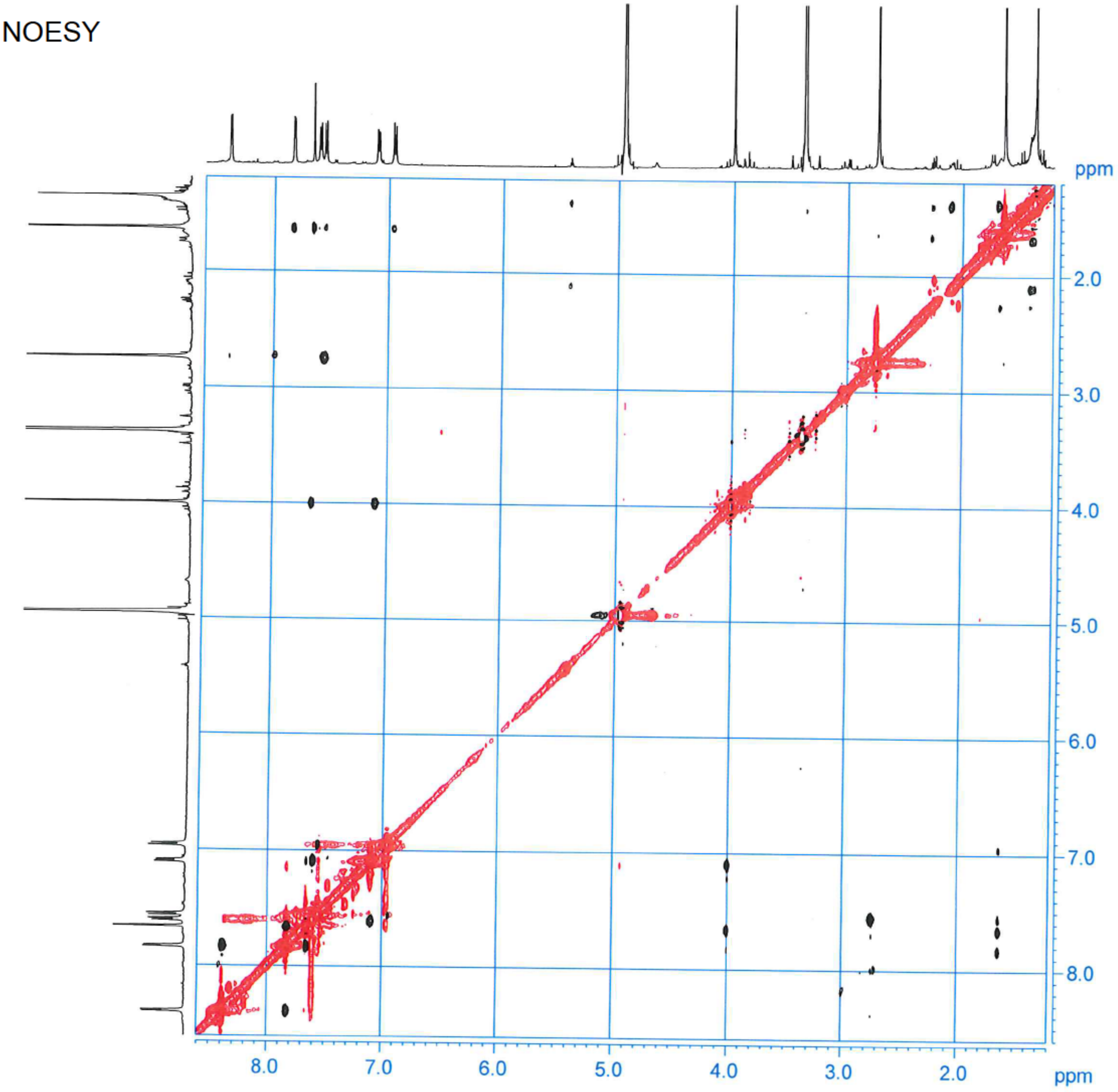

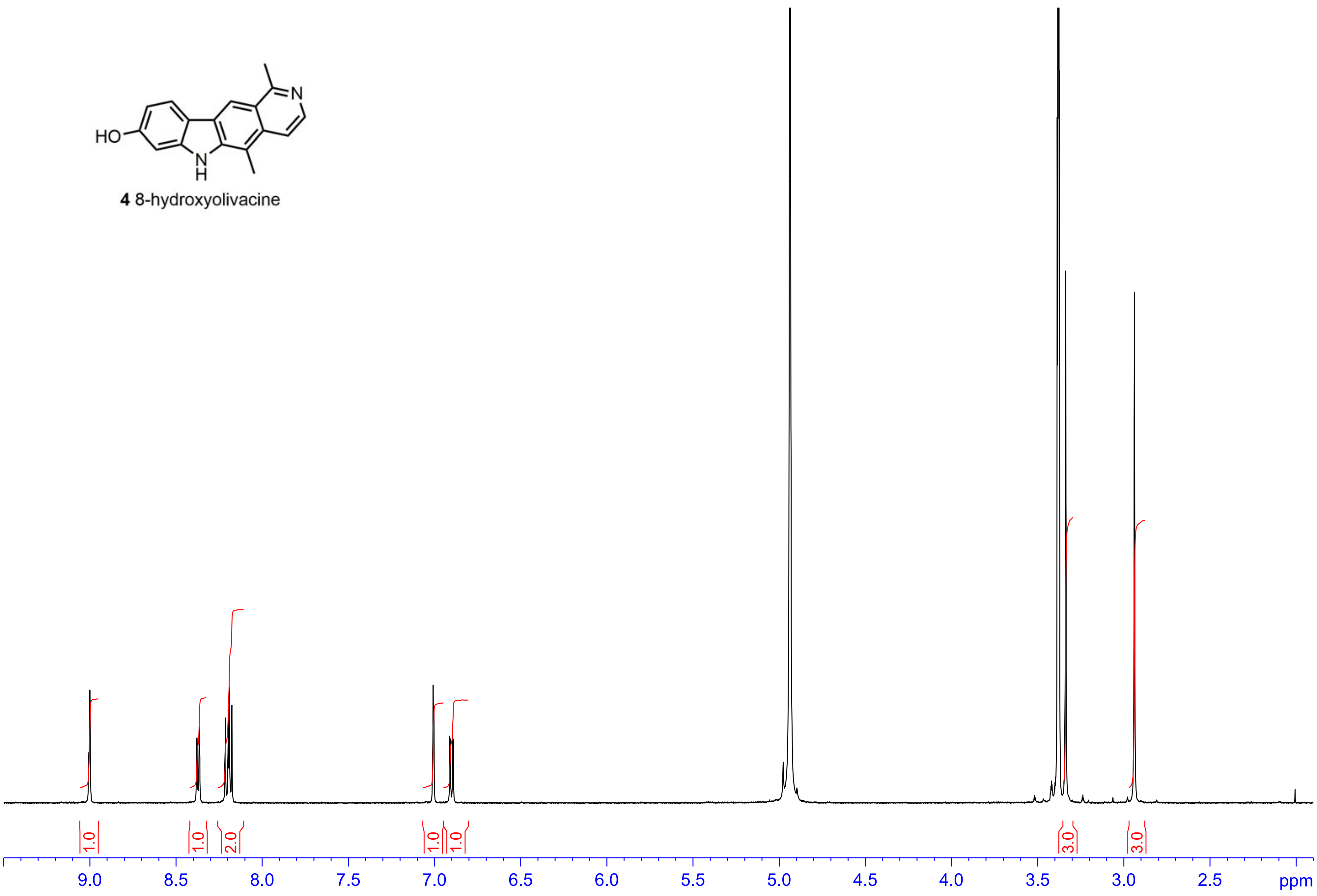
HO
N
N
H
4 8-hydroxyolivacine
1.0
1.0
2.0
1.0
1.0
3.0
3.0
9.0
8.5
8.0
7.5
7.0
6.5
6.0
5.5
5.0
4.5
4.0
3.5
3.0
2.5
ppm

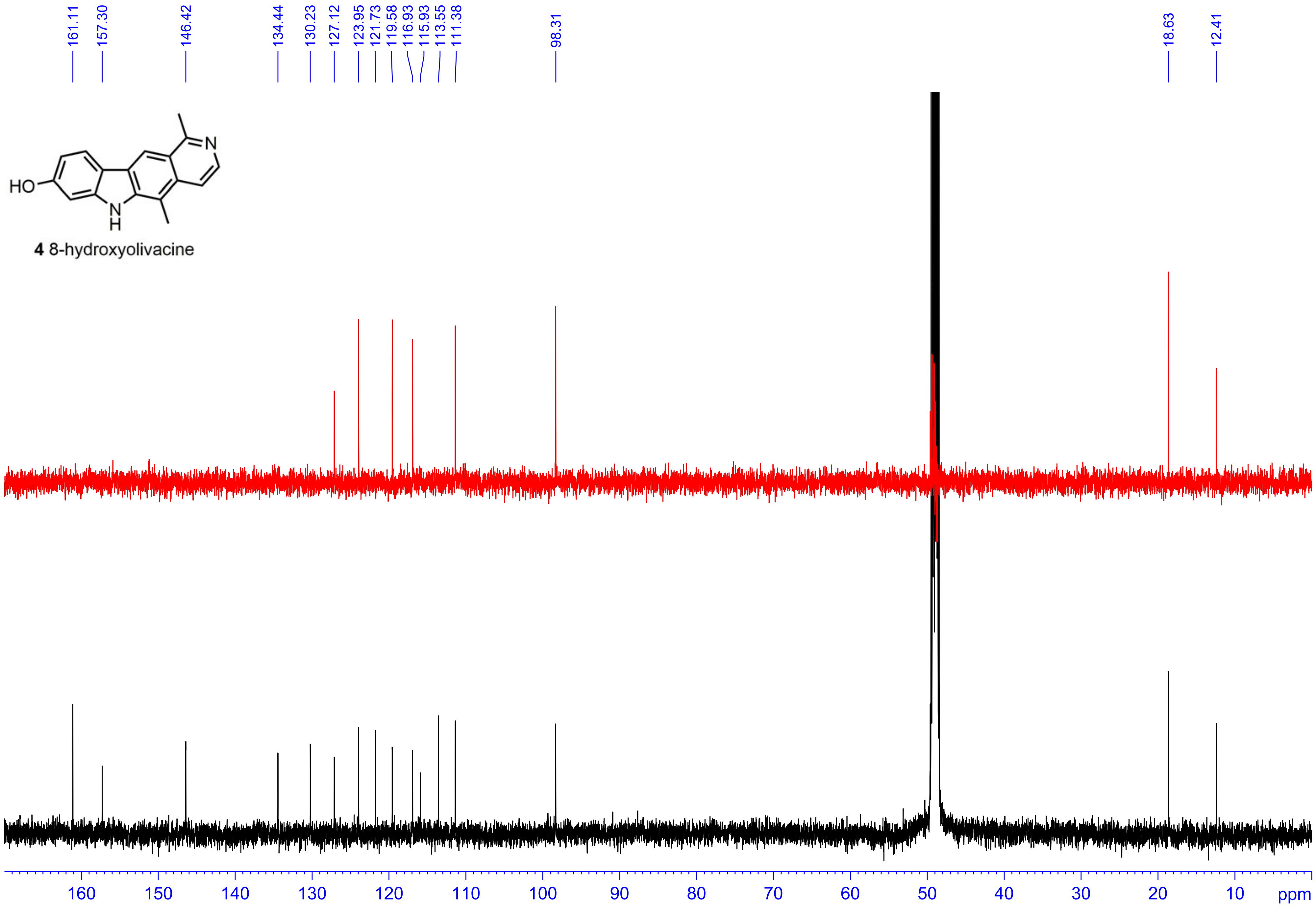

4 8-hydroxyolivacine

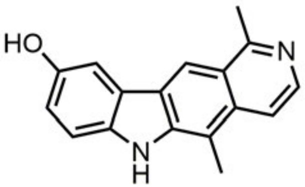

5 9-hydroxyolivacine

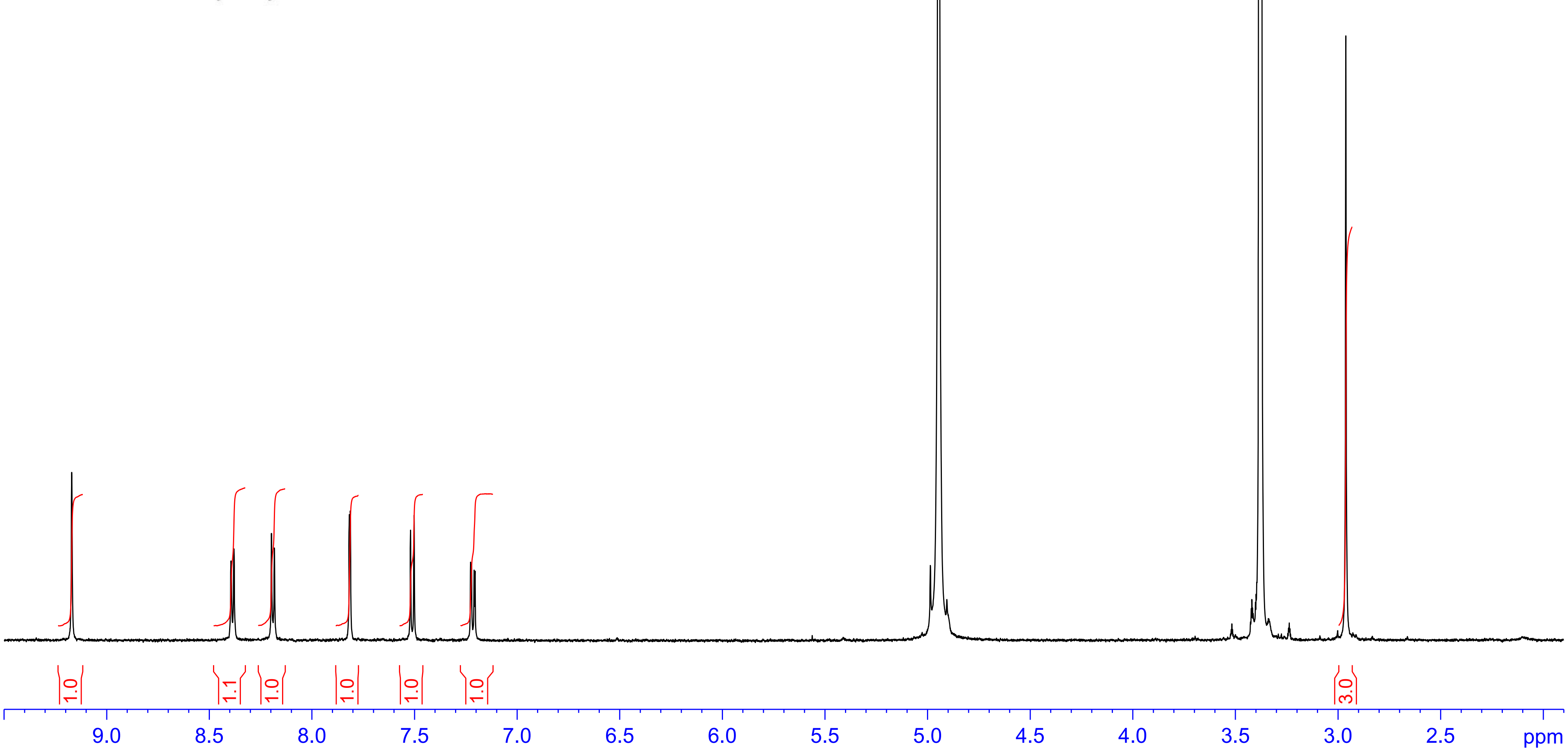

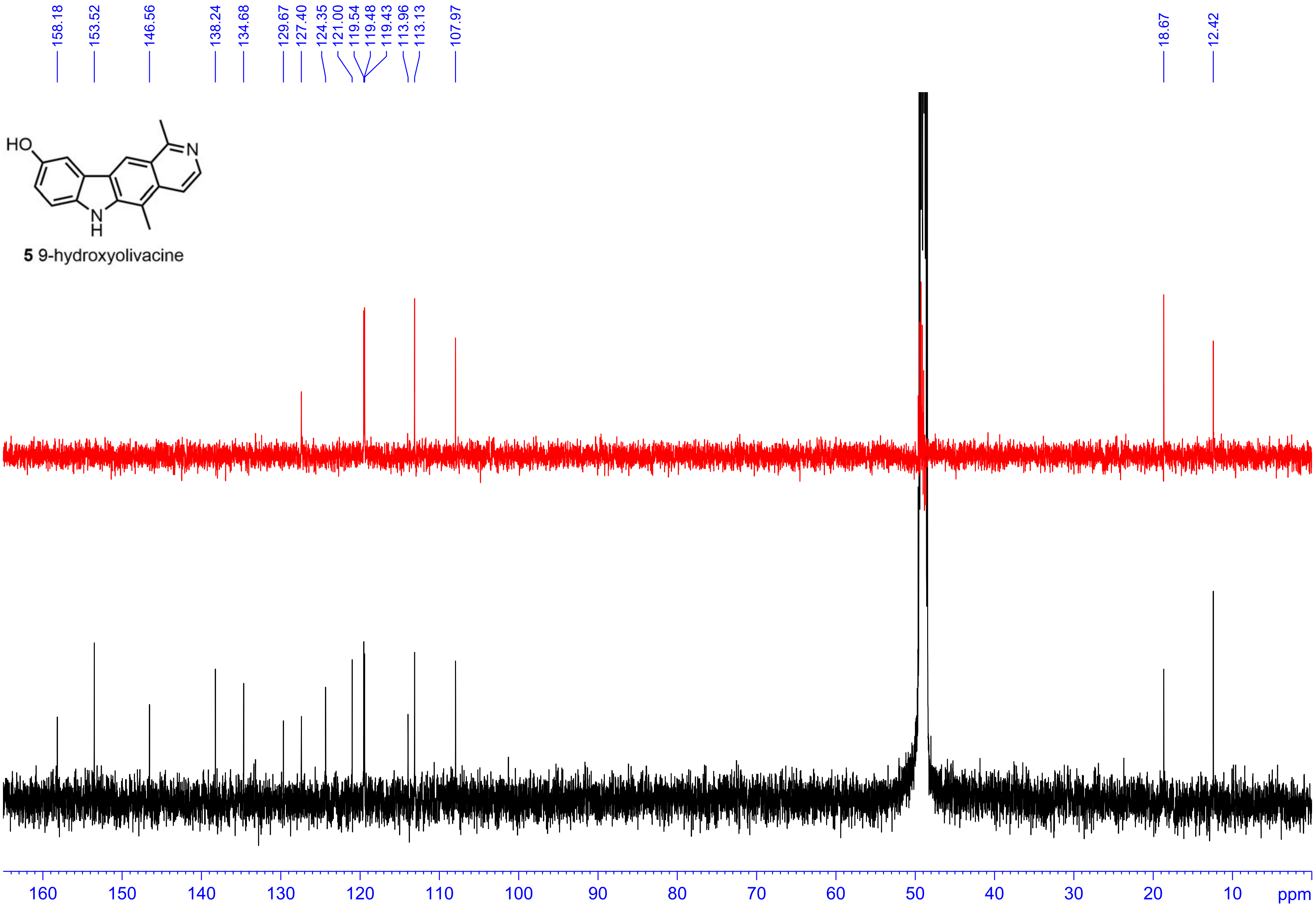

5 9-hydroxyolivacine
